# MAdCAM-1 costimulation in the presence of retinoic acid and TGF-β promotes HIV infection and differentiation of CD4^+^ T cells into CCR5^+^ T_RM_-like cells

**DOI:** 10.1371/journal.ppat.1011209

**Published:** 2023-03-10

**Authors:** Sinmanus Vimonpatranon, Livia R. Goes, Amanda Chan, Isabella Licavoli, Jordan McMurry, Samuel R. Wertz, Anush Arakelyan, Dawei Huang, Andrew Jiang, Cindy Huang, Joyce Zhou, Jason Yolitz, Alexandre Girard, Donald Van Ryk, Danlan Wei, Il Young Hwang, Craig Martens, Kishore Kanakabandi, Kimmo Virtaneva, Stacy Ricklefs, Benjamin P. Darwitz, Marcelo A. Soares, Kovit Pattanapanyasat, Anthony S. Fauci, James Arthos, Claudia Cicala

**Affiliations:** 1 Laboratory of Immunoregulation, National Institute of Allergy and Infectious Diseases, Bethesda, Maryland, United States of America; 2 Graduate Program in Immunology, Department of Immunology, Faculty of Medicine Siriraj Hospital, Mahidol University, Bangkok, Thailand; 3 Center of Excellence for Microparticle and Exosome in Diseases, Department of Research and Development, Faculty of Medicine Siriraj Hospital, Mahidol University, Bangkok, Thailand; 4 Oncovirology Program, Instituto Nacional de Câncer, Rio de Janeiro, Brazil; 5 Eunice Kennedy-Shriver National Institute of Child Health and Human Development, Bethesda, Maryland, United States of America; 6 Georgiamune, Gaithersburg, Maryland, United States of America; 7 Lymphoid Malignancies Branch, National Cancer Institute, Bethesda, Maryland, United States of America; 8 Bioinformatics Program, St. Bonaventure University, St. Bonaventure, New York, United States of America; 9 Research Technologies Section, Genomics Unit, Rocky Mountain Laboratory, National Institutes of Allergy and Infectious Diseases, Hamilton, Montana, United States of America; 10 Department of Genetics, Universidade Federal do Rio de Janeiro, Rio de Janeiro, Brazil; Emory University, UNITED STATES

## Abstract

CD4^+^ tissue resident memory T cells (T_RM_s) are implicated in the formation of persistent HIV reservoirs that are established during the very early stages of infection. The tissue-specific factors that direct T cells to establish tissue residency are not well defined, nor are the factors that establish viral latency. We report that costimulation via MAdCAM-1 and retinoic acid (RA), two constituents of gut tissues, together with TGF-β, promote the differentiation of CD4^+^ T cells into a distinct subset α_4_β_7_^+^CD69^+^CD103^+^ T_RM_-like cells. Among the costimulatory ligands we evaluated, MAdCAM-1 was unique in its capacity to upregulate both CCR5 and CCR9. MAdCAM-1 costimulation rendered cells susceptible to HIV infection. Differentiation of T_RM_-like cells was reduced by MAdCAM-1 antagonists developed to treat inflammatory bowel diseases. These finding provide a framework to better understand the contribution of CD4^+^ T_RM_s to persistent viral reservoirs and HIV pathogenesis.

## Author summary

Although antiretroviral drugs strongly suppress viral replication in HIV infected subjects, viral reservoirs persist. To achieve a cure, these reservoirs need to be eliminated. However, the location and identity of persistently infected cells are not well understood. Tissue resident memory CD4^+^ T cells (T_RM_s) are a cell type that may play an important role. These cells reside in tissues for extended periods of time and circulate infrequently. To increase our understanding of the role of T_RM_s in HIV reservoirs this study identifies MAdCAM-1 and retinoic acid (RA) as two key components of the gut immune system involved in the generation of T_RM_s. Treatment of primary CD4^+^ T cells with MAdCAM-1 and RA renders cells susceptible to HIV infection and primes them to adopt a T_RM_-like phenotype. Addition of a TGF-β, an anti-proliferative cytokine, induces them to adopt a more complete T_RM_-like phenotype. This study provides a new approach toward studying the persistent reservoirs that are a barrier to an HIV cure.

## Introduction

In the early stages of HIV infection, gut inductive lymphoid sites, including Peyer’s patches, are preferentially targeted. The consequent damage to gut-associated lymphoid tissues (GALT) plays a significant role in HIV pathogenesis [1–5]. Trafficking of naïve CD4^+^ T cells to these inductive sites is mediated by a series of interactions involving T cell homing receptors and their cognate ligands [6,7]. Integrin α_4_β_7_ (α_4_β_7_) and MAdCAM-1 play a central role in this process. MAdCAM-1 binding to α_4_β_7_ delivers a costimulatory signal to α_4_β_7_-expressing CD4^+^ T cells, including naïve CD4^+^ T cells [8,9]. We previously reported that, unlike memory CD4^+^ T cells, MAdCAM-1-dependent proliferation of naïve CD4^+^ T cells requires the addition of retinoic acid (RA), a vitamin A metabolite that is generated by dendritic cells in Peyer’s patches [9].

The combination of MAdCAM-1 and RA induces a distinct differentiation program in naïve CD4^+^ T cells that leads to the generation of CD4^+^ T cells with an α_4_β_7_^high^ phenotype that supports viral replication [9]. We suggested that the combination of MAdCAM-1 and RA, which is primarily localized to GALT and genital mucosa, might contribute to the gut-tropic nature of HIV in the acute phase of infection. Like MAdCAM-1, the V2 domain of the gp120 HIV envelope protein binds to and signals through α_4_β_7_ [10–12]. This activity may also contribute to HIV gut tropism.

What remained unclear from the above-mentioned studies was the manner in which naïve CD4^+^ T cells differentiated following MAdCAM-1 + RA costimulation. Depending upon various factors, such costimulation can drive CD4^+^ T cells toward a central memory (T_CM_) or effector memory (T_EM_) phenotype. In the past decade, a third memory T cell subset, identified as tissue resident memory T cells (T_RM_), has been described [13–16]. Because these cells reside primarily in tissues and circulate infrequently, they are difficult to harvest, and their role in HIV pathogenesis is less well defined. Several studies have suggested that T_RM_s may contribute to the formation of persistent viral reservoirs [17–20]. In this study we determined that MAdCAM-1 + RA can, in combination with TGF-β, promote the formation of CCR5^+^ cells bearing a T_RM_-like phenotype, similar to the CCR5^+^ T_RM_s that Prilc and colleagues recently identified in human rectal mucosa [21]. By demonstrating that factors associated with the gut tissue milieu can drive CD4^+^ T cells to adopt a T_RM_-like cell phenotype, this study provides a basis to better define the role of T_RM_s in HIV pathogenesis.

## Results

### RNA transcription profile in MAdCAM-1 and gp120 V2 costimulated CD4^+^ T cells

**Fig 1:**
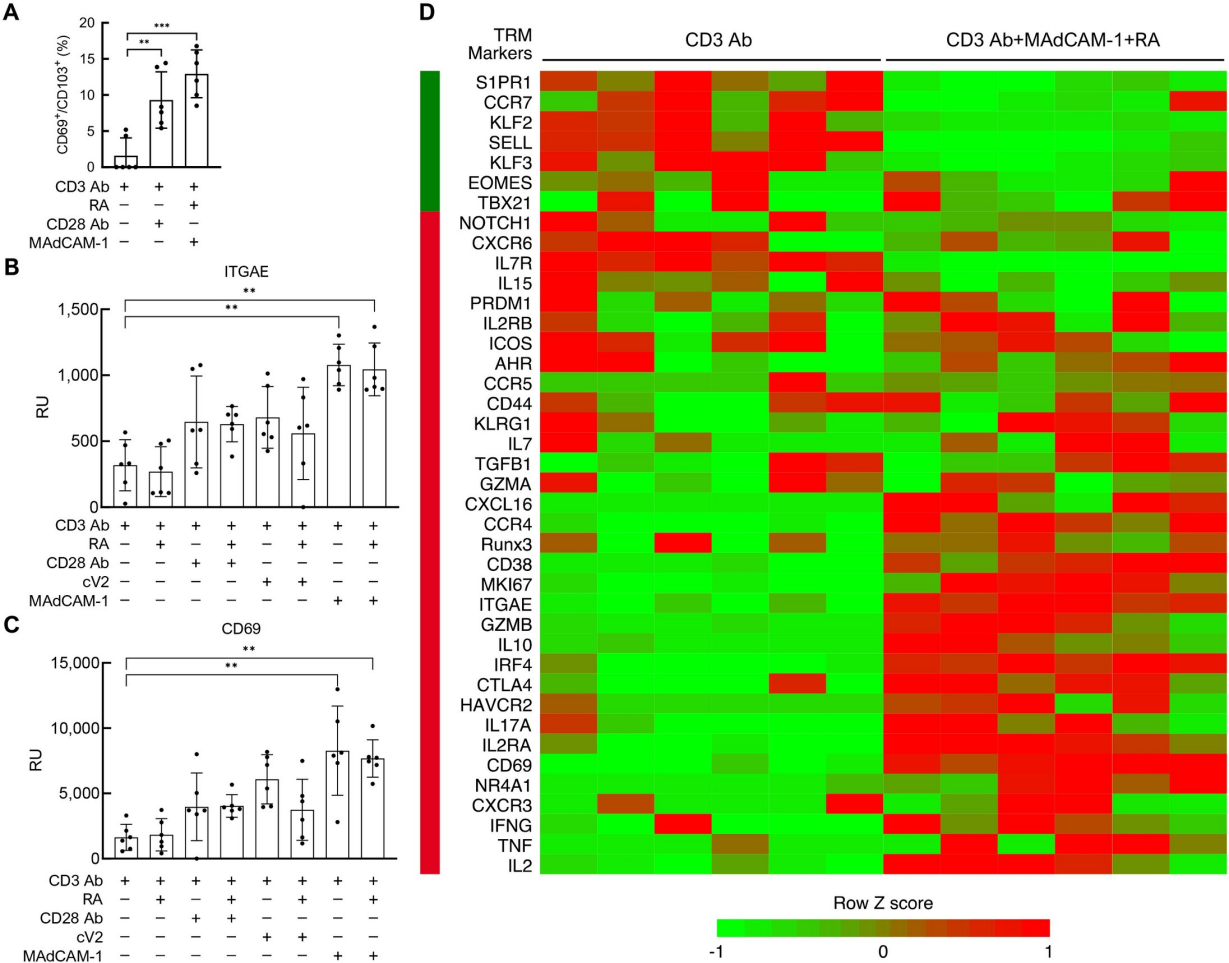
Transcription profile of MAdCAM-1 costimulated CD4^+^ T cells. (**A**) % CD69^+^/CD103^+^composition in bulk RNA-Seq samples stimulated with CD3 Ab, CD3 Ab + CD28 Ab, and CD3 Ab + MAdCAM-1 + RA as estimated by CIBERSORT. Average (**B**) ITGAE and (**C**) CD69 RNA levels from CD4^+^ T cells of 6 donors costimulated with CD3 Ab alone, CD3 Ab + cV2, CD3 Ab + MAdCAM-1, and CD3 Ab + CD28 Ab in the presence or absence of RA, detected by RNA-Seq. Y-axis represents relative units. Error bars indicate SD. (**D**) Heatmap comparing average RNA expression levels from 6 donors for 40 selected genes associated with T_RM_ cells following stimulation with CD3 Ab vs CD3 Ab + MAdCAM-1 + RA. Each column represents one donor, each row represents a selected gene. Side bar indicates direction of gene modulation (green: decrease, red: increase) in T_RM_s, as previously reported. (*: P < 0.05, **: P < 0.01, ***: P < 0.001 two-tailed t test).

In previous studies we reported that purified CD4^+^ T cell cultures derived from healthy donor PBMCs proliferated when costimulated with a CD3 antibody (Ab) and MAdCAM-1-Ig (MAdCAM-1) [9]. Costimulation with a cyclic peptide derived from the V2 loop of gp120 (cV2) also drove CD4^+^ T cell proliferation [11]. Starting cultures were typically comprised of ~40–60% naïve T cells, with the remainder comprised of memory cell subsets. Of note, purified naïve CD4^+^ T cell cultures could also proliferate, but only if RA was added to culture supernatants [9]. To better understand the CD4^+^ T cell differentiation program driven by MAdCAM-1 + RA or cV2 + RA, we carried out gene expression profiling by RNA-Seq. Primary CD4^+^ T cells were isolated from the PBMCs of 6 healthy donors. Cultures were stimulated with a CD3 Ab in the presence of either MAdCAM-1 or cV2 as previously described [9,11]. For comparison, we also stimulated cells with CD3 Ab alone and CD3 Ab in combination with a CD28 Ab. All treatments were performed in the presence or absence of RA. Cells were harvested and RNA was isolated at 24 and 48 hrs. Bulk RNA-Seq results and an RNA-Seq analysis pipeline were used to generate genome-wide gene expression profiles. The RNA-Seq gene expression values we obtained represent the combined signals from all cell subsets. We then analyzed this gene-expression data set with CIBERSORT, a recently improved bioinformatics tool, in conjunction with a customized reference matrix of leukocyte cell type signature genes derived from previously published gene expression profiles [22,23]. In our customized matrix, ITGAE was employed as the defining maker of T_RM_ cells. Additional details are included in the methods section. CIBERSORT provided an estimate of the T cell subset composition ratios in each of our bulk RNA-Seq samples. We noted a marked enrichment of T_RM_ cell ratios in the MAdCAM-1 + RA treatment group (Fig 1A and S1 Table). In an unsupervised genome-wide gene set enrichment analysis (GSEA) [24], the term T_RM_ ranked at the top of biological terms in the MAdCAM-1 + RA treatment group, but not in the CD28 Ab treatment group (S2 Table). To validate these findings, we examined the transcription of the two markers most frequently used to define T_RM_ cells: CD103 and CD69 [25,26]. CD103 (integrin α_E_) forms a heterodimer exclusively with integrin β_7_ [27], and its expression is indicative of α_E_β_7_ positivity. α_E_β_7_ facilitates retention of T cells in gut tissues [26,28]. Transcription of ITGAE (CD103 gene) was upregulated ~3-fold by MAdCAM-1, in either the presence or absence of RA, while CD28 Ab and cV2 showed a ~2-fold increase (Fig 1B). The 2^nd^ marker, CD69, is a C-type lectin that is considered an early but transient marker of T cell activation. However, prolonged expression of CD69 is a hallmark of T_RM_ cells [25]. At 24 hrs after MAdCAM-1 stimulation, CD69 transcription showed a ~5-fold increase, in either the presence or absence of RA (Fig 1C). Stimulation with either CD28 Ab or cV2 showed a more modest (~2-4-fold) increase in CD69 transcription. These findings prompted us to interrogate our RNA-Seq results with an extended panel of genes associated with T_RM_ cells. From 53 recent publications we composed a database of 99 genes associated with T_RM_s (S3 Table). Of these, 45 were strongly associated with T_RM_s (see methods section). We next generated a gene expression heatmap for 40 of these genes that were modulated by CD3 Ab + MAdCAM-1 + RA stimulation relative to CD3 Ab stimulation alone (Fig 1D). In most instances, all six donors either increased or decreased transcription of these genes in a manner consistent with T_RM_ cell gene expression. Three genes whose expression level appeared to be modulated were selected and independently confirmed by RT-qPCR (S1A Fig).

T cell differentiation is regulated, in part, by transcription factors (TFs). Several TF genes linked to T_RM_ differentiation were modulated in our RNA-seq data set in a way that is consistent with differentiation toward a T_RM_ phenotype. A schematic of TF gene modulation is presented in S1B Fig. T-Bet, Nurr77, and Ahr were upregulated, while Eomes, TCF1, and KLF2 were downregulated [29–38].

A subset of T_RM_ cells in mucosal tissues exhibits a Th17 phenotype that includes the production of IL-17, IFN-γ, IL-21 and IL-22 [21,39,40]. We observed increased transcription of IL17A in 5 of 6 donors (Fig 1D). This result prompted us to carry out a multiplex cytokine analysis of culture supernatants. We compared cytokine secretion following MAdCAM-1 or cV2 costimulation in the absence vs. presence of RA. Both induced low-level secretion of IL-17F and IFN-γ in the absence of RA. These levels were increased markedly in the presence of RA (S2 and S3 Figs). MAdCAM-1 induced TNF-α secretion ~10-fold in the presence of RA. IL-2, which fell below the limit of detection (4 pg/ml) in cells stimulated with MAdCAM-1 in the absence of RA, increased secretion to ~900 pg/ml in the presence of RA. Similarly, MAdCAM-1 induced IL-21 in the presence of RA.

From these analyses we conclude that both MAdCAM-1 and cV2 costimulation mediate a generally similar response, in agreement with our previous studies [9,11]. MAdCAM-1 appeared to provide a stronger stimulus. Treatment with MAdCAM-1, and particularly the combination of MAdCAM-1 + RA, induced cells to adopt a T_RM_-like transcription profile. Gene expression analyses can be error-prone. We therefore tentatively concluded that MAdCAM-1 + RA might be priming cells to adopt a gene expression program consistent with T_RM_ differentiation. To strengthen this conclusion we next asked whether the canonical signatures of T_RM_ cells were present on MAdCAM-1 + RA costimulated cultures of primary CD4^+^ T cells.

### Induction of CD69, CD103, CCR9 and CCR5

**Fig 2:**
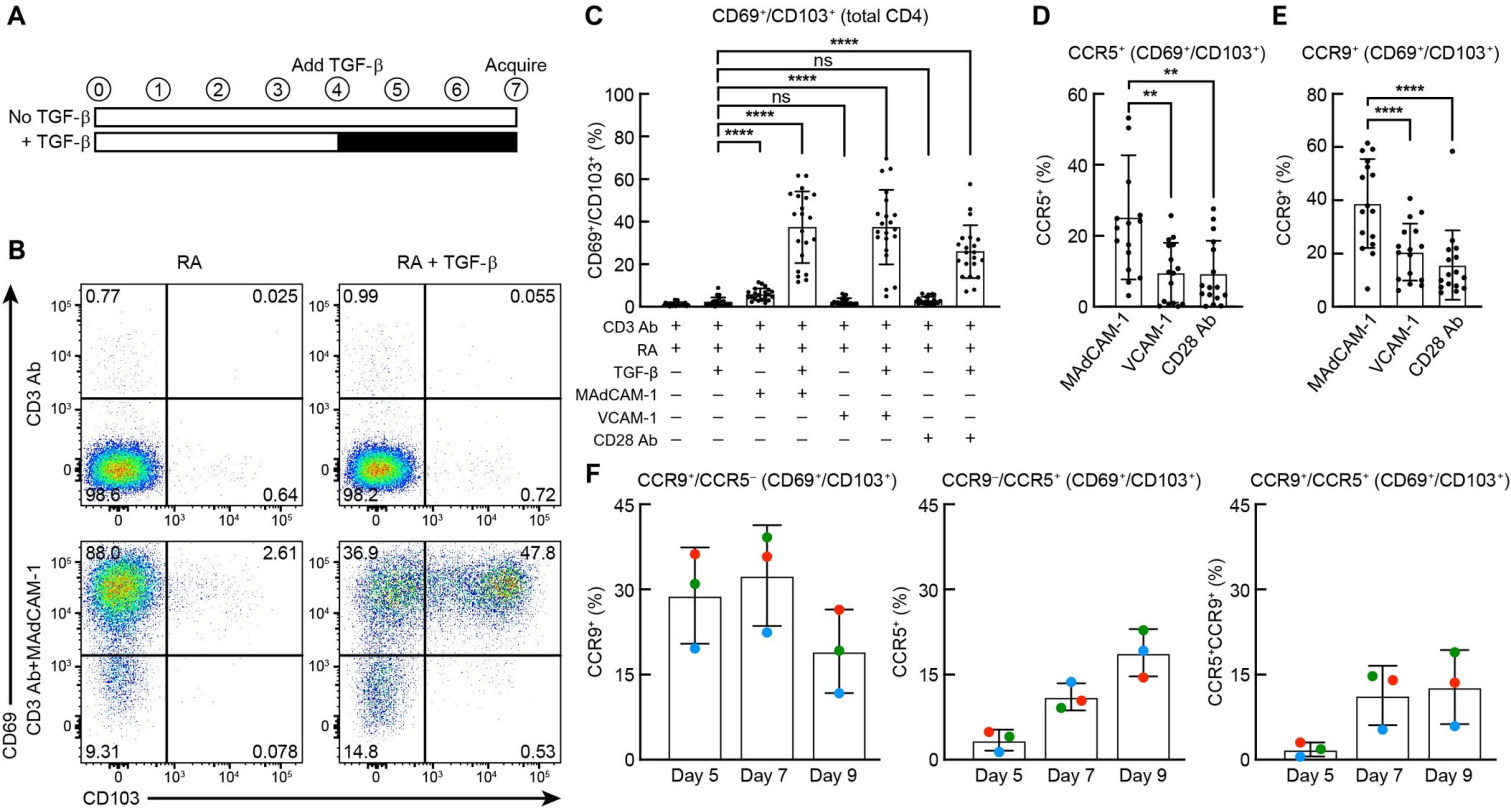
Induction of CD69, CD103, CCR9 and CCR5. (**A**) Schematic of treatment time course. (**B**) Representative flow cytometry dot plot of CD4^+^ T cells stimulated with CD3 Ab, and CD3 Ab + MAdCAM-1 in presence of RA or RA+TGF-β as indicated. Y-axis: CD69, X-axis: CD103. Percent total cells in each quadrant, as indicated. (**C)** Percent expression of CD69^+^/CD103^+^ cells within total CD4^+^ T cell cultures of 21 donors stimulated with CD3 Ab + RA, CD3 Ab + MAdCAM-1 + RA, CD3 Ab + VCAM-1 +RA, and CD3 Ab + CD28 Ab + RA, in absence or presence of TGF-β, as indicated. (**D**) Percent expression of CCR5 and (**E**) CCR9 within the CD69^+^/CD103^+^ population of 16 donors following MAdCAM-1, VCAM-1 and CD28 Ab stimulation in the presence of RA and TGF-β. (**F**) Percent expression of CCR9^+^/CCR5^-^, CCR9^-^/CCR5^+^, and CCR9^+^/CCR5^+^ cells within the CD69^+^/CD103^+^ population from 3 donors on days 5, 7, and 9 following MAdCAM-1 + RA + TGF-β stimulation. (**: P < 0.01, ****: P < 0.0001, two-tailed t test).

We began our phenotypic analysis of MAdCAM-1 + RA costimulated CD4^+^ T cells by evaluating the cell-surface expression of CD69 and CD103. Because TGF-β is known to modulate CD103 expression [41], we also included cultures with MAdCAM-1 + RA + TGF-β. Bulk CD4^+^ T cells were isolated from PBMCs and placed into wells coated with CD3 Ab and MAdCAM-1. For comparison, we included control cultures costimulated with a CD28 Ab and VCAM-Ig (VCAM-1). VCAM-1 delivers costimulatory signals through α_4_β_7_, but also through α_4_β_1_, which is expressed ubiquitously on peripheral CD4^+^ T cells [42,43]. RA was included in all cultures. Cells were initially stimulated for 4 days in the absence of TGF-β (Fig 2A). On day 4, culture media was renewed and TGF-β was added where indicated. Cells were harvested on day 7 and multicolor flow-cytometric analysis was performed. A representative result of CD69 and CD103 expression following stimulation with CD3 Ab, CD3 Ab + MAdCAM-1, CD3 Ab + TGF-β, and CD3 Ab + MAdCAM-1 + TGF-β, all in the presence of RA is shown in Fig 2B. In 21 independent donors, the addition of MAdCAM-1 + RA was sufficient to induce a low but significant number of CD69^+^/CD103^+^ cells (average 5.9%) (Fig 2C). With the addition of TGF-β, all three costimulatory ligands induced the co-expression of CD69 and CD103 on a subset of cells. Because CD69/CD103 co-expression is a hallmark of T_RM_ cells, herein we refer to these cells as T_RM_-like, recognizing, as discussed below, that this is an approximation. The capacity of MAdCAM-1 to induce the surface expression of both CD69 and CD103 agrees with the increased transcription of these genes described above. In the presence of RA, all three costimulatory ligands mediated increased expression of α_4_β_7_, both in the absence and presence of TGF-β (S4A Fig). Under certain conditions, α_4_β_7_ expression is reportedly downregulated when CD103, in the form of α_E_β_7_, appears on the cell surface [28,44]; however, we found that, in the presence of RA and TGF-β, α_4_β_7_ expression was retained on the majority of CD69^+^/CD103^+^ (i.e. CD69^+^/α_E_β_7_^+^) cells (S4B Fig). We next evaluated the expression of two chemokine receptors, CCR5 and CCR9, on MAdCAM-1-derived T_RM_-like cells. CCR9 is the gut homing chemokine receptor [45] and has been described as a marker of gut T_RM_s [46]. CCR5 is the HIV coreceptor most frequently utilized in the early stages of infection [47]. Cells were costimulated in the presence of RA + TGF-β as described above. In 16 donors, MAdCAM-1 costimulation resulted in a significantly higher level of both CCR5 and CCR9 on T_RM_-like cells relative to either VCAM-1 or CD28 Ab costimulation (Figs 2D and 2E and S5). Upregulation of CCR5 may render cells susceptible to HIV infection (see below).

We next asked whether CCR9 and CCR5 were co-expressed or appeared on different subsets of CD69^+^/CD103^+^ cells. Expression was evaluated at days 5, 7, and 9 following MAdCAM-1 + RA + TGF-β stimulation. Maximum CCR9 and CCR5 expression occurred on day 7 and 9, respectively (Fig 2F). Of note, within the time points we measured the majority of cells expressed either chemokine receptor, with only a minority of cells expressing both.

In summary, MAdCAM-1, in the absence of TGF-β, promoted the formation of a subset of cells with a CD69^+^/CD103^+^ phenotype. The frequency of these cells increased with the addition of TGF-β. CD28 Ab and VCAM-1 also induced T_RM_-like cells but only in the presence of TGF-β. In the presence of RA and TGF-β, MAdCAM-1-derived T_RM_-like cells expressed higher levels of either CCR9 or CCR5 in comparison to VCAM-1 or CD28 Ab stimulated cells.

### CD28 Ab but not MAdCAM-1 or VCAM-1 induces Tregs

**Fig 3:**
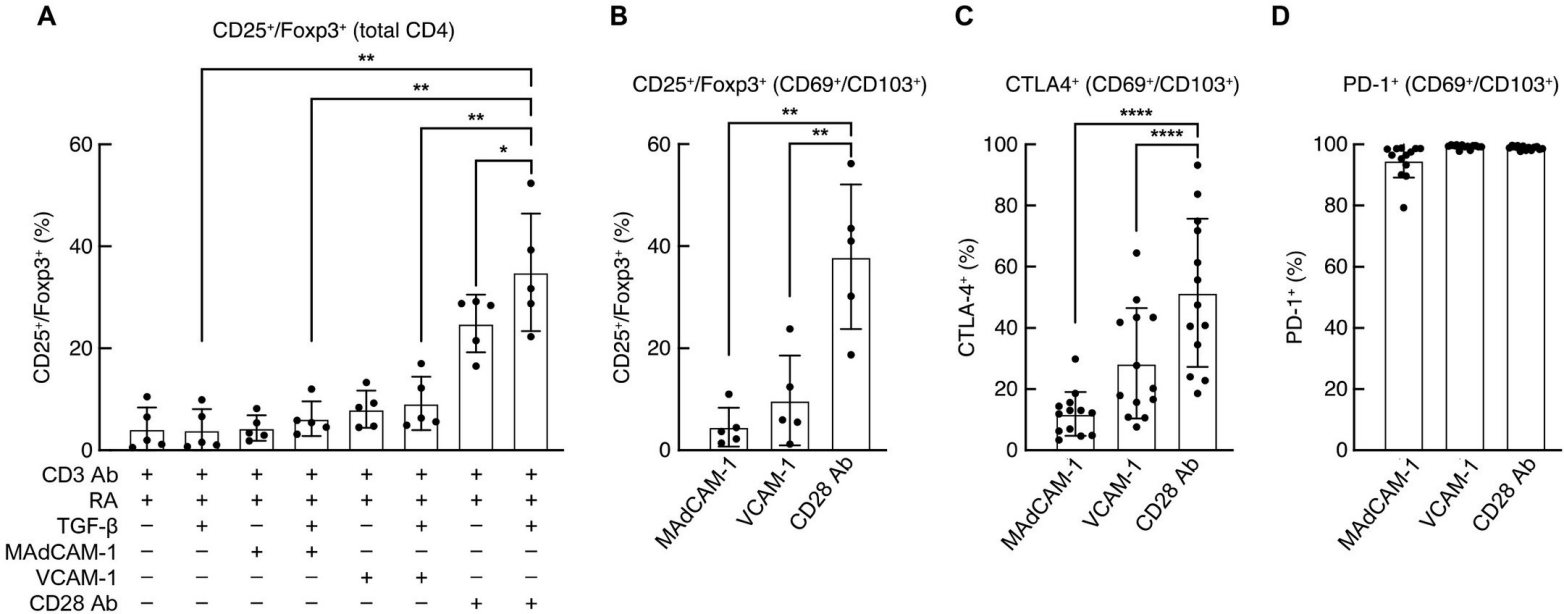
CD25/FoxP3, PD-1 and CTLA-4 expression following costimulation. Flow cytometric analysis of (**A**) CD25/FoxP3 co-expression on total CD4^+^ T cells following costimulation with MAdCAM-1, VCAM-1 and CD28 Ab in the presence of RA and TGF-β as indicated (n = 5). Expression of (**B**) CD25/FoxP3 (n = 5), (**C**) CTLA-4 (n = 13), and (**D**) PD-1 (n = 13) within the CD69^+^/CD103^+^ population following costimulation with MAdCAM-1, VCAM-1 and CD28 Ab, in the presence of RA and TGF-β (*: P < 0.05, **: P < 0.01, ****: P < 0.0001, two-tailed t test).

TGF-β and RA have been reported to work together to drive cells toward a Treg phenotype [48–51]. We asked whether MAdCAM-1 + RA + TGF-β resulted in a differentiation pattern consistent with Tregs. Cells were costimulated with MAdCAM-1, VCAM-1, or CD28 Ab as described above and stained for intracellular FoxP3 and CD25, two markers associated with Tregs (Fig 3A). However, a FoxP3^+^/CD25^+^ phenotype is not always reflective of Treg function and should be considered in that regard. As described by others, costimulation with CD28 Ab + RA yielded a ~6-fold increase in FoxP3^+^/CD25^+^ cells relative to CD3 Ab + RA. This upregulation was enhanced further by the addition of TGF-β. Surprisingly, neither MAdCAM-1 + RA nor VCAM-1 + RA, with or without TGF-β, mediated a significant increase in the frequency of FoxP3^+^/CD25^+^ cells. This pattern held true within the T_RM_-like cell subpopulation (Fig 3B). In mucosal tissues, Tregs exhibit increased FoxP3 and CTLA-4 expression when compared to circulating Tregs [21]. We found that CTLA-4 was upregulated on T_RM_-like cells induced by CD28 Ab + RA + TGF-β, and to a lesser extent on MAdCAM-1 and VCAM-1 costimulated cells (Fig 3C). Finally, we found that all three costimulatory ligands, when combined with RA and TGF-β, induced similarly high levels of PD-1 on T_RM_-like cells (Fig 3D). The failure of MAdCAM-1 and VCAM-1 to induce the expression of FoxP3 and CD25 underscores the way in which different costimulatory signals can influence cell differentiation in critical ways.

We noted above that five of six donor CD4^+^ T cells exhibited increased transcription of IL17A (Fig 1D). We followed this observation by measuring the secretion of cytokines with a multiplex cytokine array. The combination of MAdCAM-1 + RA induced the secretion of IL-17F and IFN-γ (S2 and S3 Figs). These measurements were made at time points prior to TGF-β treatment, which we carry out on day 4 following costimulation. This prompted us to address the type of cell polarization induced by MAdCAM-1 + RA following TGF-β treatment. To this end, we measured surface expression markers associated with Th17 (CCR6) and Th1 (CXCR3) on day 7 by flow cytometry. Approximately half of the CD69^+^/CD103^+^ cells expressed CXCR3. Only low levels of CCR6 were observed (S6 Fig). These data indicate IL-17 production prior to the addition of TGF-β and suggest a degree of Th1 polarization on day 7. Th polarization is highly plastic [52–54] and our findings are consistent with such plasticity.

### Role of RA and TGF-β in MAdCAM-1 generation of CD4^+^ T_RM_-like cells

**Fig 4:**
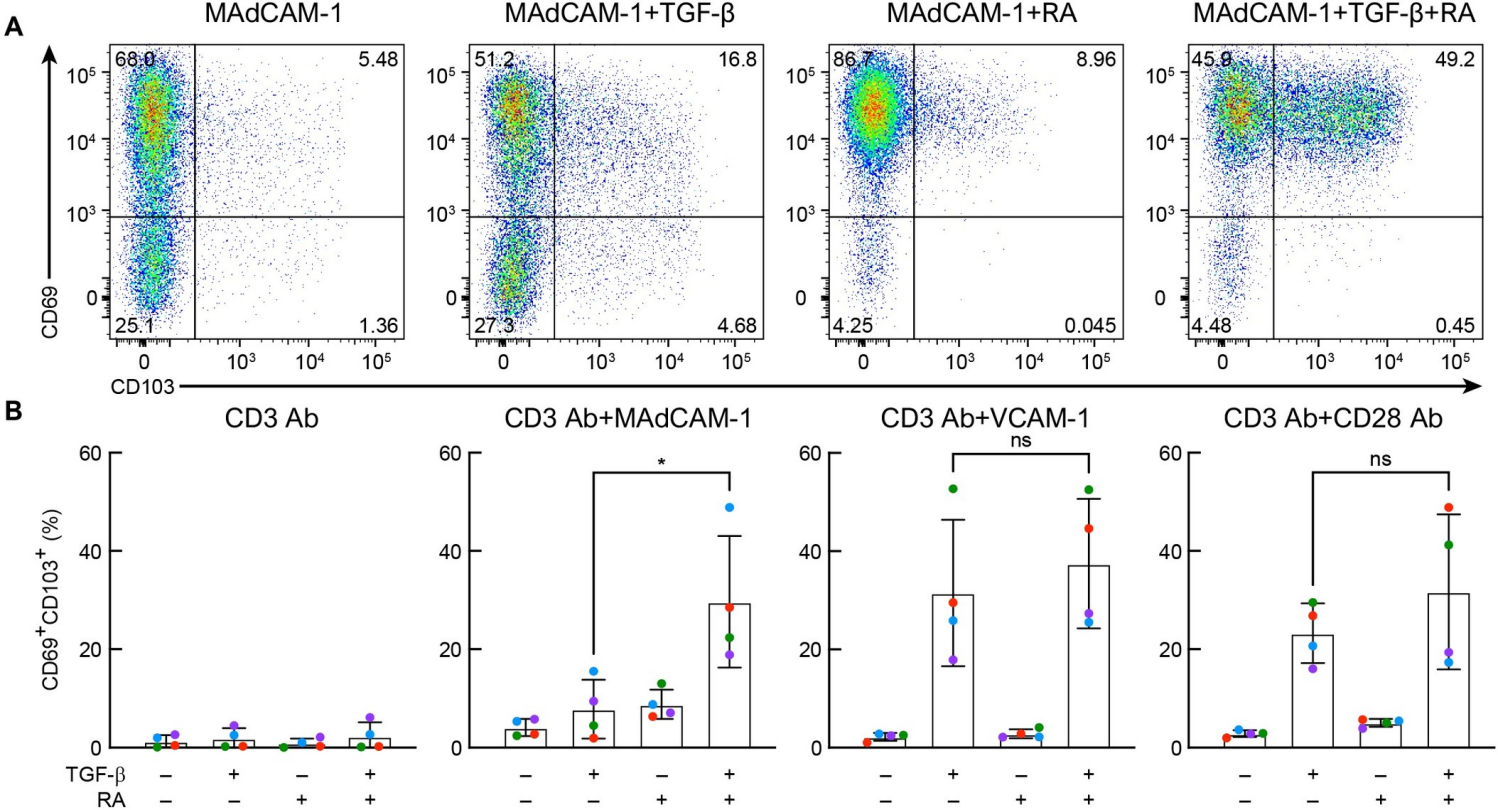
Effect of RA and TGF-β on CD69^+^/CD103^+^ expression. (**A**) Representative flow cytometry dot plot of CD69 (Y-axis) and CD103 (X-axis) on CD3 Ab + MAdCAM-1 stimulated CD4^+^ T cells in the absence or presence of RA and TGF-β, as indicated. Percent of total cells in each quadrant, as indicated. (**B**) Average percent CD69^+^/CD103^+^ cells following CD3 Ab alone, CD3 Ab + MAdCAM-1, CD3 Ab + VCAM-1 or CD3 Ab + CD28 Ab stimulation in the absence or presence of RA and TGF-β as indicated (n = 4). (*: P < 0.05, two-tailed t test).

MAdCAM-1 can signal through α_4_β_7_ but not through α_E_β_7_. RA increases the surface expression of α_4_β_7_ by upregulating integrin β_7_ (ITGB7) [45]. TGF-β upregulates integrin α_E_ (CD103) which pairs exclusively with integrin β_7_ [41]. With this dynamic in mind, we compared the impact of RA vs TGF-β on MAdCAM-1-mediated induction of CD69^+^/CD103^+^ T_RM_-like cells. Cells were costimulated and analyzed for CD69 and CD103 expression, as described above. A representative comparison of MAdCAM-1 costimulation with all four combinations of RA and TGF-β is presented (Fig 4A), along with results from four donors stimulated with each of the three costimulatory ligands (Fig 4B). CD3 Ab + MAdCAM-1 alone, or in the presence of either RA or TGF-β, induced cells presenting a CD69^+^/CD103^+^ phenotype at a low frequency (~4–9%). Combining MAdCAM-1 + RA + TGF-β increased significantly the frequency of T_RM_-like cells. These results suggest that, in the absence of RA, low levels of integrin β_7_ on the cell surface limit the formation of α_4_β_7_, which in turn limits MAdCAM-1-mediated costimulation. The combination of RA and TGF-β allows cells to respond to MAdCAM-1, while concomitantly upregulating CD103. VCAM-1 and CD28 Ab can costimulate cells independently of α_4_β_7_, and they require only TGF-β to induce T_RM_s. These observations underscore the way in which MAdCAM-1 and RA, a combination that is linked to the gut milieu, when combined with TGF-β, can promote the formation of relatively high levels of CD69^+^/CD103^+^ T_RM_-like cells. T_RM_-like cells induced by MAdCAM-1 costimulation exhibit properties distinct from those generated by CD28 Ab and VCAM-1, as will be further described below.

### Influence of RA and TGF-β on CCR5 and CCR9 expression

**Fig 5:**
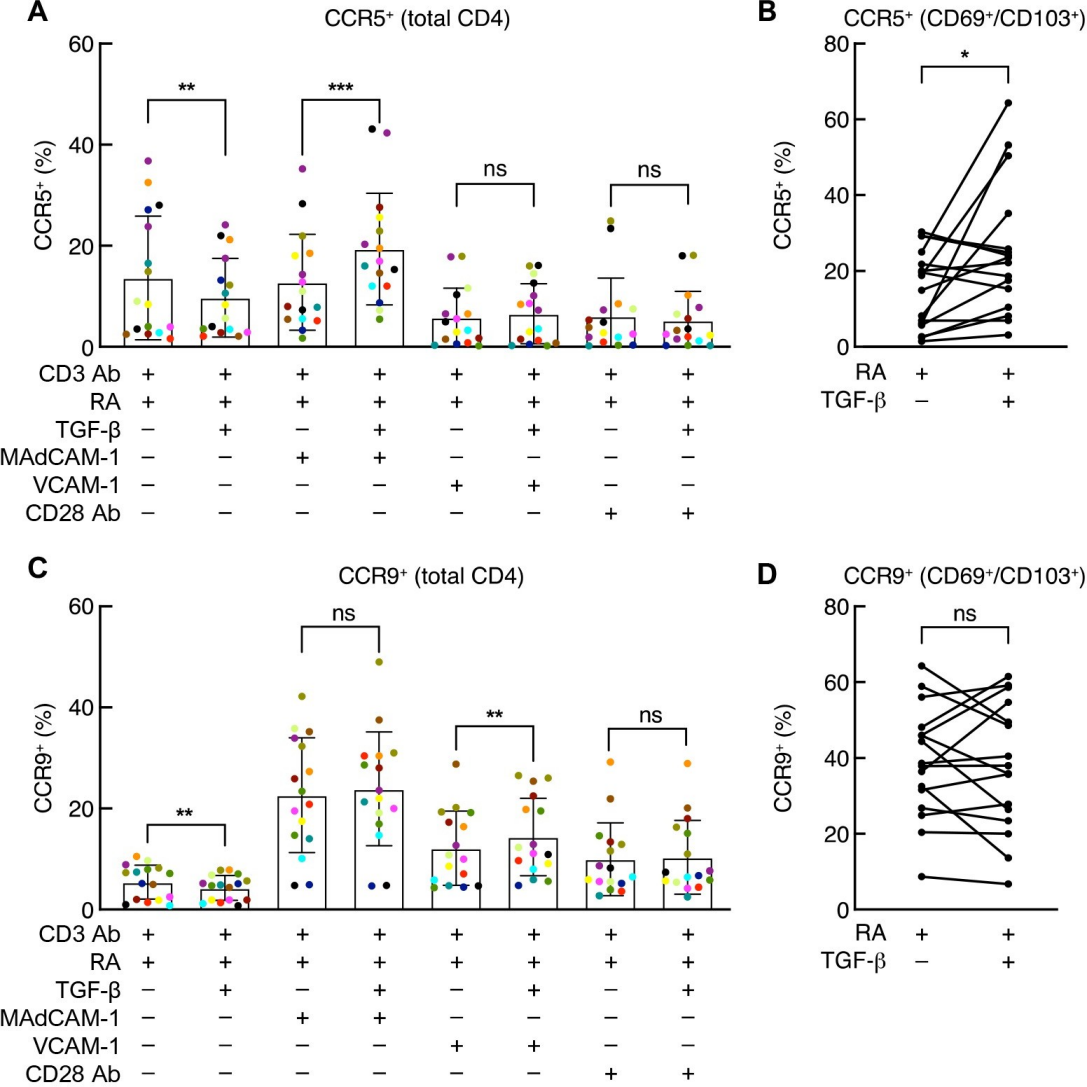
TGF-β effect on CCR5 and CCR9 expression. Flow-cytometric analysis of CCR5 and CCR9 expression on total CD4^+^ T cells (**A** and **C**) or CD69^+^/CD103^+^ CD4^+^ T cells (**B** and **D**) costimulated with MAdCAM-1 + RA, VCAM-1 + RA, or CD28 Ab + RA, in the absence or presence of TGF-β, as indicated (n = 16) (*: P < 0.05, **: P < 0.01, ***: P < 0.001, two-tailed t test).

The capacity of RA to induce the expression of both CCR9 and α_4_β_7_ underlies its role in CD4^+^ T cell trafficking to GALT [45]. Less understood is the effect of combining RA with TGF-β on CCR5 and CCR9 expression. To address this issue, CD4^+^ T cells were costimulated with MAdCAM-1 + RA, VCAM-1 + RA and CD28 Ab + RA as described above in the absence or presence of TGF-β. CCR5 and CCR9 expression in the total CD4^+^ T cell population, and within the CD69^+^/CD103^+^ population, were determined on day 7. Surprisingly, in 16 independent donors, the addition of TGF-β to cells stimulated with MAdCAM-1 + RA increased the frequency of CCR5 expressing cells by an average ~1.5-fold (Fig 5A). This increase also appeared within the CD69^+^/CD103^+^ subpopulation (Fig 5B). TGF-β did not increase CCR5 expression in VCAM-1 + RA or CD28 Ab + RA treated cells. To our knowledge, this effect of combining TGF-β with MAdCAM-1 has not been previously observed. TGF-β had little impact on the expression of CCR9 on CD4^+^ T cells for any of the three costimulatory ligands, either in the total CD4^+^ T cell population or in the MAdCAM-1 + RA treated CD69^+^/CD103^+^ subpopulation (Fig 5C and 5D). In summary, in the presence of RA, TGF-β increased the expression of CCR5 on CD4^+^ T cells, including on CD69^+^/CD103^+^ T_RM_-like cells. For CD69^+^/CD103^+^ cells, this effect was observed only in the context of MAdCAM-1 costimulation, underscoring the distinct way in which combining MAdCAM-1 with RA and TGF-β impacts the differentiation of CD4^+^ T cells.

### α_4_β_7_ antagonists inhibit the differentiation of CD4^+^ T cells into T_RM_-like cells

**Fig 6:**
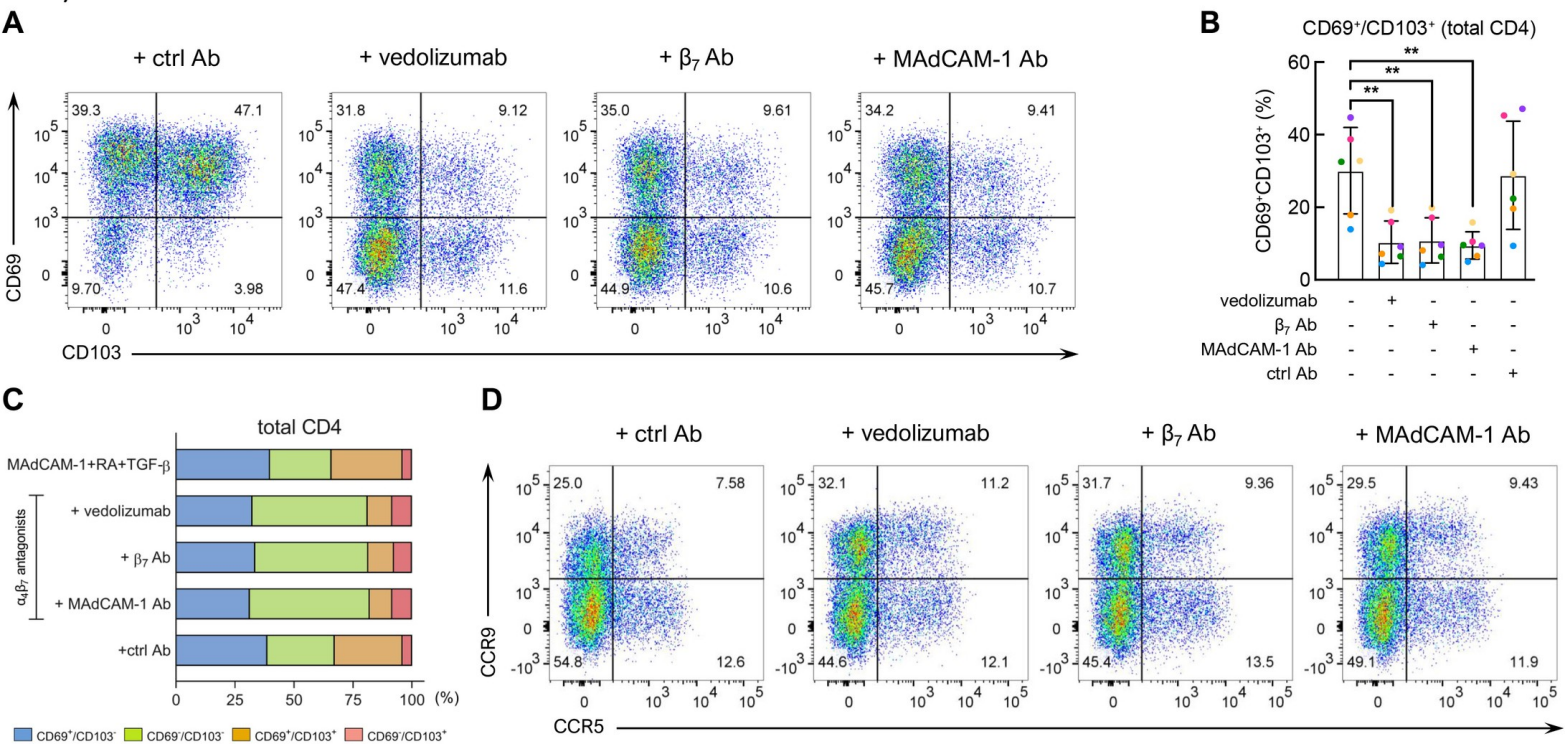
α_4_β_7_ antagonists inhibit T_RM_-like cell differentiation. (**A**) Representative flow cytometry dot plot of CD69 (Y-axis) and CD103 (X-axis) expression following costimulation with MAdCAM-1 + RA + TGF-β in the absence or presence of vedolizumab, β_7_ Ab or MAdCAM-1 Ab, as indicated. (**B**) Flow-cytometric analysis of CD69^+^/CD103^+^ CD4^+^ T cell frequency, as in panel A in 6 independent donors. Y-axis indicates the frequency of CD69^+^/CD103^+^ CD4^+^ T cells. Inclusion of α_4_β_7_ antagonists indicated below. (**: P < 0.01, two-tailed t test). (**C**) Bar graph representing the average relative frequencies of CD69^-^/CD103^+^ (red), CD69^+^/CD103^+^ (orange), CD69^-^/CD103^-^ (green), and CD69^+^/CD103^-^ (blue) cell populations in the absence or presence of α_4_β_7_ antagonists in six independent donors, as indicated. (**D**) Representative flow cytometry dot plot of CCR9 (Y-axis) and CCR5 (X-axis) expression on total CD4^+^ T cells following costimulation with MAdCAM-1 + RA + TGF-β in the absence or presence of vedolizumab, β_7_ Ab or MAdCAM-1 Ab, as indicated. Percent total cells in each quadrant, as indicated.

Vedolizumab is a humanized α_4_β_7_ mAb that interferes with MAdCAM-1 binding. In recent years it has become a front-line therapeutic in the treatment of inflammatory bowel diseases (IBD) [55]. Subsequently, related antagonists have been developed as alternative therapies. Etrolizumab, which targets integrin β_7_, is a humanized derivative of FIB504, a mouse mAb specific to integrin β_7_ [56]. Ontamalimab (PF-00547659) is a human MAdCAM-1 Ab [57,58]. Although the initial rationale for employing these mAbs in the treatment of IBD involved their potential to inhibit CD4^+^ T cell migration into GALT, recent data suggest that their mode of action may involve other mechanisms [9,59–61]. We asked whether vedolizumab along with a primatized FIB504 (β_7_ Ab) and the 314G8 (MAdCAM-1 Ab) [62], with a specificity similar to ontamalimab, could inhibit the formation of T_RM_-like cells. Cells were costimulated with MAdCAM-1 + RA as described above. On day 4, TGF-β was added with or without each of the three mAb antagonists. All three mAbs inhibited CD69^+^/CD103^+^ T_RM_-like cell differentiation in a significant way (Fig 6A and 6B). In six independent donors, vedolizumab, β_7_ Ab, and MAdCAM-1 Ab each inhibited T_RM_-like cell differentiation by ~65%, while a control mAb showed no significant inhibition. There were several ways in which these α_4_β_7_ antagonists altered cell phenotypes in unexpected ways. CD69^-^/CD103^+^ cells typically appear at a low frequency following MAdCAM-1 + RA + TGF-β stimulation. Interestingly, all three α_4_β_7_ antagonists increased the frequency of CD69^-^/CD103^+^ cells by ~2-fold (Fig 6C). Additionally, all three antagonists failed to reduce the expression of either CCR9 or CCR5 (Figs 6D and S7A and S7B). Finally, we found that MAdCAM-1 Ab did not reduce the expression of integrin β_7_ (S7C Fig).

### HIV infection in T_RM_s

**Fig 7:**
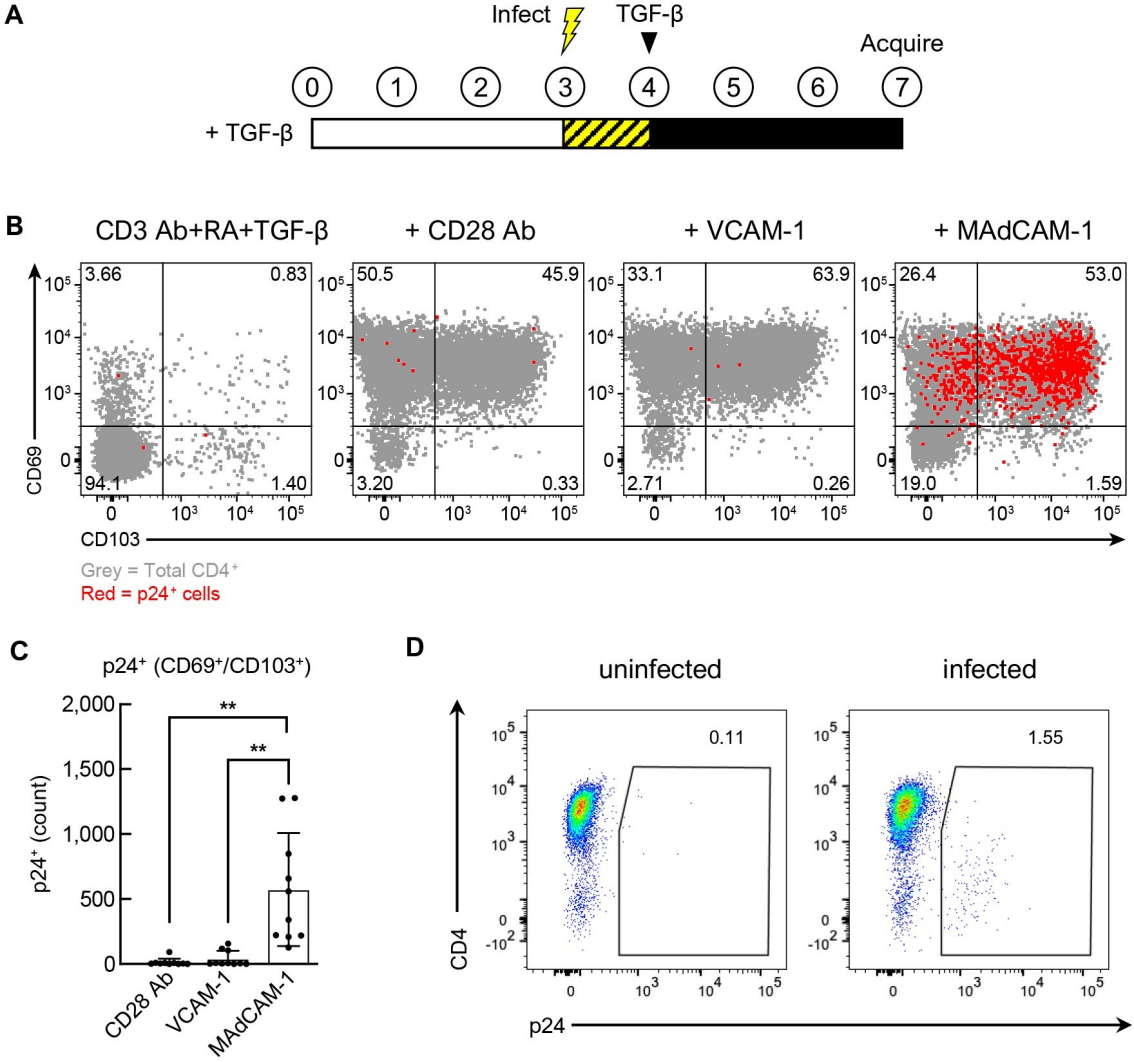
HIV infection following costimulation in the presence of RA and TGF-β. (**A**) Schematic of infection time course. (**B**) Representative flow cytometry dot plot indicating HIV infection of CD4^+^ T cells costimulated with CD28 Ab, VCAM-1 or MAdCAM-1 in the presence of RA and TGF-β (Y-axis: CD69, X-axis: CD103). p24^+^ cells shown in red. (**C**) Average number of p24^+^ cells in the CD69^+^/CD103^+^ population of CD4^+^ T cells following costimulation as in **B** (n = 10). (**D**) Representative dot plot of CD4 downregulation in p24^+^ cells. X-axis: p24, Y-axis: CD4. (**: P < 0.01, two-tailed t test).

The capacity of TGF-β, when combined with RA, to induce CCR5 on MAdCAM-1-derived T_RM_-like cells raised the possibility that MAdCAM-1+ RA costimulation might support HIV infection, while TGF-β could subsequently drive these cells further toward a T_RM_-like cell phenotype. To address this issue, we costimulated cells with MAdCAM-1 + RA + TGF-β as described above. VCAM-1 + RA + TGF-β and CD28 Ab + RA + TGF-β were also tested. Sixteen hours prior to the addition of TGF-β, cells were inoculated with an R5-tropic HIV isolate (SF162). Three days after the addition of TGF-β, cells were stained with two HIV p24 mAbs [63], along with anti-CD69 and anti-CD103 (Fig 7A). For most donors, little infection was observed in the absence of a costimulatory ligand. For a small number of donors, we observed infection in the presence of CD3 Ab alone, likely due to a preactivated state of the donor PBMCs. A representative infection of CD69^+^/CD103^+^ T_RM_ cells is provided (Fig 7B), along with results from 10 donors (Fig 7C). To ensure that intracellular p24 staining reflected infection, we employed the R5-antagonist maraviroc (S8 Fig). While CD28 Ab, VCAM-1 and MAdCAM-1 all promoted the formation of T_RM_-like cells, only MAdCAM-1 + RA costimulation consistently supported infection. As expected, CD4 was substantially downregulated on p24^+^ cells (Fig 7D). These observations are consistent with the capacity of MAdCAM-1, but not VCAM-1 or CD28 Ab, to upregulate CCR5. However, we found that MAdCAM-1 + RA costimulation also supported X4-tropic HIV infection, suggesting that factors other than CCR5 may be involved as well (S8 Fig).

## Discussion

The types of cells that contribute to formation of persistent viral reservoirs that form in the first weeks following infection (Fiebig I/II) are a subject of great interest and active investigation [63,64]. Estes and colleagues estimated that >90% of viral RNA positive cells that persist after ART therapy reside in the gut [65,66]. Although the precise identity of these cells is unknown, several studies have demonstrated that α_4_β_7_^high^ memory CD4^+^ T cells that reside in gut tissues are preferentially infected in Fiebig I/II [67–70]. It is unknown whether T_RM_s contribute in a significant way to viral reservoirs. Because T_RM_s can persist in tissues for decades without recirculating, establishing their contribution to viral reservoirs is challenging. Moreover, there is no established way to generate T_RM_s in vitro. Here we report that stimulating CD4^+^ T cells with MAdCAM-1 and RA, both of which are associated with CD4^+^ T cell trafficking to GALT, in combination with TGF-β, a cytokine that suppresses T cell proliferation, drives these cells toward a phenotype consistent with T_RM_s. These cells express CD103 and CD69, two canonical markers of T_RM_s, along with α_4_β_7_ and CCR9, two receptors that facilitate gut homing. A significant fraction of these T_RM_-like cells express CCR5. We find that MAdCAM-1 + RA costimulated cells support infection, and the subsequent addition of TGF-β drives the cells to adopt a T_RM_-like cell phenotype. In this regard, we note that TGF-β can be anti-proliferative for CD4^+^ T cells. Of note, CCR5-expressing CD69^+^/CD103^+^/CD4^+^ T_RM_s have been identified in rectal mucosa [21]. The model described herein for generating T_RM_-like cells from peripheral blood CD4^+^ T cells provides a platform to advance our understanding of the factors that drive differentiation of CD4^+^ T cells toward a T_RM_ phenotype, as well as the role that this subset of cells might play in the formation of viral reservoirs.

In previous studies, we and others reported that HIV preferentially infects α_4_β_7_-expressing CD4^+^/CD45RO^+^ T cells in vitro and in vivo [68,71]. In the course of these studies we determined that, in the presence of RA, signaling by both HIV gp120 and MAdCAM-1 activates cells in a way that supports HIV infection [1,9,11]. Both RA and MAdCAM-1 are modulated in the context of HIV and SIV infection [72,73]. These observations raise the question as to how signaling through α_4_β_7_ impacts CD4^+^ T cell differentiation. In this report we find that MAdCAM-1 provides a distinct costimulatory signal. We found that other costimulatory signals, i.e. VCAM-1 and CD28 Ab, when combined with RA and TGF-β, also generate T_RM_-like cells. However, VCAM-1 and CD28 Ab do not upregulate CCR5 to the same extent as does MAdCAM-1. Additionally, these cells do not support infection in the same manner as MAdCAM-1.

It is well established that RA upregulates CCR9 and integrin β_7_ [45]. We and others have previously noted that RA can also upregulate CCR5 [9,74–77]. In this report, we show that MAdCAM-1 + RA costimulation followed by TGF-β treatment promotes the differentiation of primary CD4^+^ T cells into CD69^+^/CD103^+^ cells that, for the most part, express either CCR9 or CCR5, with few CCR9^+^/CCR5^+^ cells. We previously reported that CCR5 colocalizes with α_4_β_7_ and CD4 on both RA-treated CD4^+^ T cells and CD4^+^ T cells isolated from colon biopsies [71]. Although we do not know whether these three receptors work together, it is intriguing to consider that HIV gp120 signals through all three [78].

Although MAdCAM-1 + RA alone was sufficient to induce low levels of CD69^+^/CD103^+^ T_RM_-like cells, addition of TGF-β increased their frequency in a significant way. We were surprised to find that TGF-β also upregulated CCR5. This effect only occurred in cells treated with RA and most prominently in those costimulated with MAdCAM-1. It is unclear how the combination of MAdCAM-1 and RA predisposes cells to respond to TGF-β in this way. In any case our findings suggest that MAdCAM-1 + RA primes cells to differentiate toward a T_RM_-like phenotype.

When RA was combined with MAdCAM-1, CD4^+^ T cell cultures secreted IL-17A and IL-17F. These cells were susceptible to infection, and when TGF-β was added, infected cells could adopt a T_RM_-like phenotype. In general, TGF-β acts as an immunosuppressive and anti-proliferative cytokine. In mice, the combination of TGF-β and RA promotes CD4^+^ T cells to differentiate toward a FoxP3^+^ Treg cell phenotype [49,51]. We can speculate that CD4^+^ T cells, in gut tissues, stimulated with MAdCAM-1 and RA, when exposed to TGF-β may promote the formation of latent reservoirs. It is unclear why MAdCAM-1 is superior to either VCAM-1 or CD28 Ab with respect to supporting infection. This observation may however help explain why gut tissues generally support very high levels of viral replication during the acute phase of infection.

Several α_4_β_7_ or MAdCAM-1 mAb antagonists have been developed to treat IBD. We previously reported that one of these, vedolizumab, inhibited MAdCAM-1-mediated proliferation [9]. Here we show that vedolizumab, anti-integrin β_7_ and a MAdCAM-1 Ab, suppressed T_RM_-like cell differentiation. These antagonists altered the differentiation program of cells in an unusual way. CD69^-^/CD103^+^ cells, which are typically present at a low frequency (average ~ 4%), increased ~2-fold in the presence of these antagonists. Surprisingly, CCR9 and CCR5 expression were not significantly changed. The MAdCAM-1 Ab antagonist did not reduce integrin β_7_ expression. Thus, although all three antagonists inhibited T_RM_-like cell differentiation, their presence resulted in a distinct differentiation pattern not otherwise observed. Further study of the way that these antagonists alter CD4^+^ T cell differentiation and trafficking may shed light on their mechanism of action in ameliorating the symptoms of IBD.

The capacity of vedolizumab, β_7_ Ab and a MAdCAM-1 Ab to suppress the differentiation of T_RM_-like cells necessarily reduced infection. Such an effect may provide insight into the way that vedolizumab treatment alters infection in vivo [60]. PET/CT imaging of SIV infected macaques treated with ART reveals viral antigen in gut tissues [79]. In a follow-up study, also using PET/CT imaging, we reported that a primatized analogue of vedolizumab reduced viral antigen in the gut without depleting CD4^+^ cells [59]. In another study, the combination of vedolizumab and a bNAb reduced the frequency of lymphoid aggregates in mesenteric lymph nodes and ileum and delayed viral rebound relative to bNAb treatment alone [80]. Mehandru and colleagues administered vedolizumab to a small number of ART-treated, HIV infected subjects co-afflicted with IBD [60]. Vedolizumab reduced the number of lymphoid aggregates in the GI tract. They noted that these aggregates are important sanctuary sites for establishing and maintaining viral reservoirs [60]. The capacity of α_4_β_7_ antagonists to reduce CD69^+^/CD103^+^ T_RM_-like cell differentiation reported here, along with the above-mentioned in vivo findings, argues for further exploration into the utility of these antagonists in targeting viral reservoirs.

Among the limitations of this study is the incomplete way our in vitro system recapitulates the environment in the gut that drives the formation and maintenance of CD4^+^ T_RM_s. This difference limits the longevity of our in vitro derived T_RM_ cells. It will be important to identify soluble factors that extend the lifespan of CD4^+^ T_RM_ cells. Prlic and colleagues report that CCR5^+^ T_RM_s isolated from mucosal tissues can express IL-17 upon restimulation [21]. We did not observe this for the in vitro derived T_RM_s following restimulation. Thus, further refinements of this system will be required to increase the similarity between in vitro T_RM_-like cells and true T_RM_s present in vivo. Nevertheless, given the challenge of isolating T_RM_s from humans, the results described herein can potentially help advance our understanding of the role of T_RM_s in HIV infection. Finally, although we argue that T_RM_s may contribute to persistent HIV reservoirs, their role in these reservoirs is speculative and remains an open question.

In conclusion, in this report we have shown that MAdCAM-1 costimulation in the presence of RA and TGF-β drives CD4^+^ T cells toward a CD69^+^/CD103^+^ phenotype that is consistent with T_RM_s. In the presence of RA and TGF-β, MAdCAM-1 was unique among the costimulatory ligands we tested in inducing CCR5. We speculate that the generation of CCR5^+^ T_RM_s may contribute to the pool of persistently infected cells in gut tissues. In this regard we note that penetrance of antiretroviral drugs in the gut can be suboptimal [66,81–83]. Antagonists such as vedolizumab, already approved for the treatment of IBD, suppress MAdCAM-1 costimulation and consequently reduce T_RM_-like cell formation. These findings will hopefully spur further research into the role of CD4^+^ T_RM_s in HIV pathogenesis.

## Materials and methods

### Human blood samples and primary cell preparation

All primary CD4^+^ T cells utilized in these studies were isolated from PBMCs collected from healthy donors through a NIH Department of Transfusion Medicine protocol that was approved by the Institutional Review Board of the National Institute of Allergy and Infectious Diseases (NIAID), National Institutes of Health. Informed consent was written and was provided to study participants. PBMCs were isolated from whole blood of healthy donors by Lymphocyte Separation Medium (MP Biomedicals, Santa Clara, CA). Purified CD4^+^ T cells were derived by negative selection (Stem Cell Technologies, Vancouver, Canada) to >95% purity, as determined by flow cytometry. To ensure that an adequate number of naïve cells were present, purified CD4^+^ T cells were prescreened by flow cytometry for expression of CD45RO. Cultures with >70% CD45RO^+^ cells were not used.

### Gene expression profiling

#### RNA extraction methods

CD4^+^ T cells from 6 donors were subjected to 8 different treatments. All six donors were processed simultaneously to limit error. Approximately 2 x 10^5^ human T cells in 100 μl of culture media were lysed with 300μl Trizol LS (Thermofisher Scientific, Waltham, MA), combined with 0.2 volumes of 1-Bromo-3-chloropropane (Sigma, St. Louis, MO), samples mixed, and centrifuged at 16,000 x *g* for 15 min at 4°C. RNA containing aqueous phase was combined with equal volume of RLT buffer with 1% Beta mercaptoethanol and extracted using AllPrep DNA/RNA 96-well system (Qiagen, Valencia, CA). An additional on-column DNase I treatment was performed during RNA extraction. RNA integrity was assessed using the Agilent 2100 Bioanalyzer using RNA 6000 Pico kit (Agilent Technologies, Santa Clara, CA).

#### NGS methods

All samples were processed simultaneously. The SMART-Seq v4 Ultra Low Input RNA Kit for Sequencing (Takara Bio, San Jose, CA) was used for cDNA synthesis with an input of 150 pg total RNA. Amplified cDNA was visualized and quantified using BioAnalyzer High Sensitivity chips (Agilent Technologies, Santa Clara, CA). Three hundred picograms of purified cDNA were brought up to 15 uL in volume and sheared on the Covaris LE220 (Covaris Inc., Woburn, MA) using the shearing parameters of PIP 180, 20% DF, 50 bursts/cycle, for 270 seconds with Y-dithering of 5 mm at 20 mm/s. Sequencing ready libraries were generated with 10 uL of sheared cDNA (200 pg) using the ThruPLEX DNA-Seq Library Preparation Kit (Takara Bio, San Jose, CA) and assessed for quality on BioAnalyzer DNA1000 chips (Agilent Technologies, Santa Clara, CA). Libraries were quantified using the Kapa Quant Kit for Illumina sequencing (Kapa Biosystems, Wilmington, MA), normalized to 4 nM, pooled equally, and prepared for sequencing on the NextSeq (Illumina, San Diego, CA) following the user manual guidelines for High Output single read 75 cycle chemistry runs.

### RNA-Seq raw data processing and differentially expressed gene selection

The reads of each sample were aligned to human genome GRCh38 by STAR2 software [84], and then followed by HTseq-Count software for read counts of each gene. As a whole group of 96 samples, DESeq2 software package [85] was used to calculate the normalized expression values of genome-wide genes. For each of our 12 treatment-control comparisons (6 different treatments by 2 two time points), we conducted two-tailed t test for p-values, as well as calculated fold changes (FC) for each gene. Differentially expressed gene lists for each comparison were selected with the criteria of p<0.05 and FC> absolute (1.50). To condense the data of two time points, final differential expressed gene lists representing each treatment were generated by selecting the more significant time point of the same treatments.

### Digital cytometry analysis

A CIBERSORT reference matrix of PBMC cell subgroups was built by combining leukocyte signature genes (LM22) [22] and single cell RNA-Seq based gene expression profiles [23] (see S1 Table). Briefly, the web app Azimuth was used to process scRNA-Seq PBMC datasets. ITGAE gene expression was used as a reference for T_RM_-like cells. A virtual gene expression profile of T_RM_-like cells was generated. Bulk RNA-Seq data was then entered into the customized T_RM_ module in order to generate ratios for CD4^+^ cell subsets including T_RM_s. For given normalized bulk RNA-Seq expression profiles, CIBERSORT [22] was used to estimate the composition ratios of each cell subgroup based on the reference matrix.

### Heatmap visualization

Heatmap of gene expression values of marker genes were generated using either R ggplot2 package or Partek Genomic Suite 7. Bar plots used for presenting the expression changes of the key genes utilized by GraphPad Prism V9 (GraphPad Software, La Jolla, CA). This data has been deposited in the GSEA data base, under accession # GSE221434. You can access this data at https://www.ncbi.nlm.nih.gov/geo/query/acc.cgi?acc=GSE221434

### Customized gene set enrichment analysis

The reference marker gene sets relevant to T_RM_, T_EM_ and T_CM_ were derived from scRNA-Seq PBMC datasets [22] using FindMarkers function provided in the Seurat 4.0 package [23]. The reference marker gene sets were also manually annotated from relevant publication [29]. In addition, some immunologically relevant pathways were also collected from MSigDB [86]. These collections served as customized reference gene signatures for the gene functional enrichment analysis using Broad Institute’s Gene Set Enrichment Analysis (GSEA) software 4.1.0. [24].

### RT-qPCR

Key biological findings from the RNA-Seq were validated by RT-qPCR. Validation analysis was performed in a subset of RNA-Seq samples. The validation sample-set consisted of day 2 samples with RA treatment in 3 donors. S4 Table lists 3 selected biologically relevant genes and one house-keeping gene for RT-qPCR validation. Primer Express software for Real-Time PCR v3.0.1 (Life science technologies, Carlsbad, CA) was used to design primer sets. Selected primers and probes spanned two conserved exons with the minimum intron length of 1kb that do not cross-hybridize with paralogous gene sequences. Probe and primer sets were ordered from Biosearch Technologies (Biosearch technologies, Novato, CA).

Template cDNAs were synthesized using SuperScript VILO cDNA synthesis kit (ThermoScientific, Waltham, MA) and were purified using the QIAquick 96 PCR Purification Kit (Qiagen, Valencia, CA). The Invitrogen Express qPCR supermix with premixed ROX (Invitrogen, Carlsbad, CA) reactions were carried out in 20 uL reactions with 1X RT-PCR buffer, 400 nM of forward and reverse primers, 120 nM of fluorescent TaqMan probe(s). The qPCR reactions were carried out at 50°C for 2 min, 95°C for 2 min, and 55 cycles of 95°C for 15 sec and 60°C for 1 minute. Data was analyzed using Applied Biosystems 7900HT Sequence Detection Systems version 2.4.1 software (Life technologies, Carlsbad, CA). Normalized NGS read count values were used for correlation with qPCR data. The correlation analysis was carried out using Prism and Spearman correlations were calculated between qPCR and NGS read count values.

### Cytokine immunoassays

Cytokine concentrations in culture supernatants from 3 independent donors were determined with a bead-based immunoassay (Luminex, Human Th17 multiplex kit (HTH17MG-14K-PX25). Culture supernatants were collected at 72 hrs (MAdCAM-1) or 96 hrs (cV2). Data collection was carried out with a Luminex 200 xMAP instrument following the manufacturer’s instructions.

### Costimulation assays and tissue culture reagents

MAdCAM-1-Ig and VCAM-Ig were obtained from R&D Systems (Minneapolis, MN). CD28 Ab was purchased from Invitrogen (Carlsbad, CA). Costimulatory ligands were biotinylated per the manufacturer’s instructions using a LYNX Rapid Plus Biotin (Type 2) Antibody Conjugation Kit (Bio-Rad, Hercules CA). Costimulation assays were carried out as previously described [11] with several modifications. 96-well flat bottom cell culture-treated plates (Corning, Corning, NY) were first pre-coated with 50 ng of CD3 Ab (clone OKT3) (eBioscience, San Diego, CA) at 4°C for 2 hours, followed by 200 ng of NeutrAvidin (Invitrogen, Carlsbad, CA) at 4°C overnight in 100 μl HBS. 200 ng of biotinylated costimulatory ligand was then added for 1 hour at 37°C. 200,000 purified CD4^+^ T cells were then added to coated wells and cultured in complete RPMI 1640 medium with 2% L-glutamine-penicillin-streptomycin (Gibco Laboratories, Gaithersburg, MD) and 10% FBS (Gibco Laboratories, Gaithersburg, MD) (1 x 10^6^ cells/ml) at 37°C, 5% CO_2_. In some cultures, 10 nM all-trans retinoic acid (Sigma-Aldrich, St. Louis MO) was included. On day 4, cultures were replaced with fresh complete media. In some cultures, TGF-β (1 ng/ml) was also added on day 4. On day 7, cells were harvested and analyzed by flow cytometry using FlowJo software. In some experiments, 2 μg/ml of α_4_β_7_ mAb antagonists (vedolizumab, β_7_ Ab clone FIB504, MAdCAM-1 Ab clone 314G8) or a control mAb were added on day 4 along with TGF-β.

### Antibodies and flow cytometry

Antibody staining, carried out in 2% FBS in 1X PBS, employed standard protocols. mAbs utilized for flow cytometry are listed in S5 Table. For intracellular FoxP3 staining, cells were first stained for surface markers and then permeabilized using a FoxP3 staining buffer (eBioscience, San Diego, CA). Data was collected on a FACSCanto II (BD Biosciences, San Diego, CA) and analyzed using FlowJo. Statistical significance was determined with Prism. Two-tailed t tests were applied, and p values reported.

### Viral infection assay

A molecular clone of HIV SF162 (R5-tropic, subtype B) (accession number EU123924) was utilized in all infections. This stock was produced by transient transfection of 293T cells and then briefly passaged through PBMCs. For infection, 0.2 μl of 0.29 ng/μl (p24) was added on day 3 following MAdCAM-1, VCAM-1, or CD28 Ab costimulation. After 18 hrs, cells were rinsed 3X and replaced with fresh media. On day 7 post stimulation, cells were stained with Live/Dead Aqua (Invitrogen, Carlsbad, CA), fixed and permeabilized (Cytofix/Cytoperm, BD Biosciences, San Diego, CA) and stained with two intracellular anti p24 Gag antigen mAbs [63]. Cells were analyzed by flow cytometry and infection was determined by the percentage of cells double positive for p24 Gag antigen.

## Supporting information

S1 Fig Validation of RNA-Seq results by RT-qPCR and TF modulation. RT-qPCR was performed on RNA derived from CD4^+^ T cells costimulated with CD28 Ab, MAdCAM-1 and cV2, all in the presence of RA (n = 3). Primers and probes specific to CCR7 (left), SELL (middle) and S1PR1 (right) we employed. Values were normalized to the housekeeping gene TAF1D, and relative units (RU) reported. Error bars indicate standard deviation. (**B**) The T_RM_ lineage signal translation pathway following MAdCAM-1 + RA treatment is shown. TF genes in the pathway are depicted illustrated as ovals, with a four-color scheme of red, pink, green, and white, which respectively represent up-regulation (> 1.5 fold), moderate up-regulation (1.25–1.5 fold), down-regulation (< -1.5 fold), and no significant changes. Relevant surface receptors are depicted as rectangles.
(TIF)

S2 Fig Cytokine secretion in response to MAdCAM-1 + RA costimulation. Bead based immunoassay of cytokines in culture supernatants following CD3 Ab + MAdCAM-1 (white bar) or CD3 Ab + MAdCAM-1 + RA (black bar) costimulation of CD4^+^ T cells. Average cytokine concentration (pg/ml) from 3 independent donors. Error bars indicate standard deviation. Only the analytes that yielded signals above the limit of detection are shown.
(TIF)

S3 Fig Cytokine secretion in response to cV2 + RA costimulation. Bead based immunoassay of cytokines in culture supernatants following CD3 Ab + cV2 (white bar) or CD3 Ab + cV2 + RA (black bar) costimulation of CD4^+^ T cells. Average cytokine concentration (pg/ml) from 3 independent donors. Error bars indicate standard deviation. Only the analytes that yielded signals above the limit of detection are shown.
(TIF)

S4 Fig Co-expression of α_4_β_7_ and α_E_β_7_ on CD4^+^ T_RM_s in the presence of TGF-β. (**A**) Flow-cytometric analysis of α_4_β_7_ expression on total CD4^+^ T cells without any stimulation or with CD3 Ab + RA, CD3 Ab + MAdCAM-1 + RA, CD3 Ab + VCAM-1 + RA, or CD3 Ab + CD28 Ab + RA in the absence or presence of TGF-β as indicated. (**B**) α_4_β_7_ expression on CD69^+^/α_E_β_7_^+^ CD4^+^ T cells following CD3 Ab + MAdCAM-1, CD3 Ab + VCAM-1, or CD3 Ab + CD28 Ab, as indicated (n = 6).
(TIF)

S5 Fig Expression of CCR5 on MAdCAM-1 + RA stimulated cells. Representative flow cytometry dot plot of CCR5 expression within the CD69^+^/CD103^+^ population are shown. Cells stimulated with MAdCAM-1 (left), VCAM-1 (middle), and CD28 Ab (right) are shown. Y-axis: FSC-A, X-axis: CCR5.
(TIF)

S6 Fig CXCR3 and CCR6 expression on MAdCAM-1 costimulated cells. (**A**) Representative flow cytometric dot plot of MAdCAM-1 + RA + TGF-β treated cells. CD69^+^/CD103^+^ cells (left) were stained with CXCR3 (Y-axis) and CCR6 (X-axis). (**B**) CXCR3 (left) and CCR6 (right) expression in 5 independent donors.
(TIF)

S7 Fig CCR9, CCR5, and integrin β_7_ expression in the presence of α_4_β_7_ antagonists. Flow-cytometric analysis of (**A**) CCR9 (n = 5), (**B**) CCR5 (n = 5), (**C**) integrin β_7_ (n = 3) in the total CD4^+^ T cell population following MAdCAM-1 + RA + TGF-β costimulation in the absence or presence of vedolizumab, β_7_ Ab, or MAdCAM-1 Ab, as indicated.
(TIF)

S8 Fig Intracellular p24 staining of R5 and X4-tropic isolates of HIV in the presence of Marviroc. (**A**) Representative dot plot of an R5 tropic isolate (SF162) (upper row) and an X4-tropic isolate (NL4-3) (lower row) alone (left) or in the presence of maraviroc (middle) or T20 (right) following MAdCAM-1 + RA + TGF-β treatment. Cells shown are CD69^+^/CD103^+^. (**B**) p24 staining, as in A for 3 donors for SF162 (left) and NL4-3 (right).
(TIF)

S1 Table CIBERSORT Reference module of CD4^+^ cell subtypes following MAdCAM-1 + RA costimulation. (DOCX)

S2 Table GSEA analysis results of CD3 Ab + MAdCAM-1 + RA and CD3 Ab + CD28 Ab + RA treatment groups. (DOCX)

S3 Table T_RM_ knowledge database. (DOCX)

S4 Table RT-qPCR oligo sequences. (DOCX)

S5 Table Flow cytometry antibodies. (DOCX)
