# Supplementary figures and images for: MAdCAM-1 costimulation in the presence of retinoic acid and TGF-β promotes HIV infection and differentiation of CD4+ T cells into CCR5+ TRM-like cells
Source: PLoS Pathog. 2023 Mar 10;19(3):e1011209. doi: 10.1371/journal.ppat.1011209 (PMC10032498; doi:10.1371/journal.ppat.1011209)

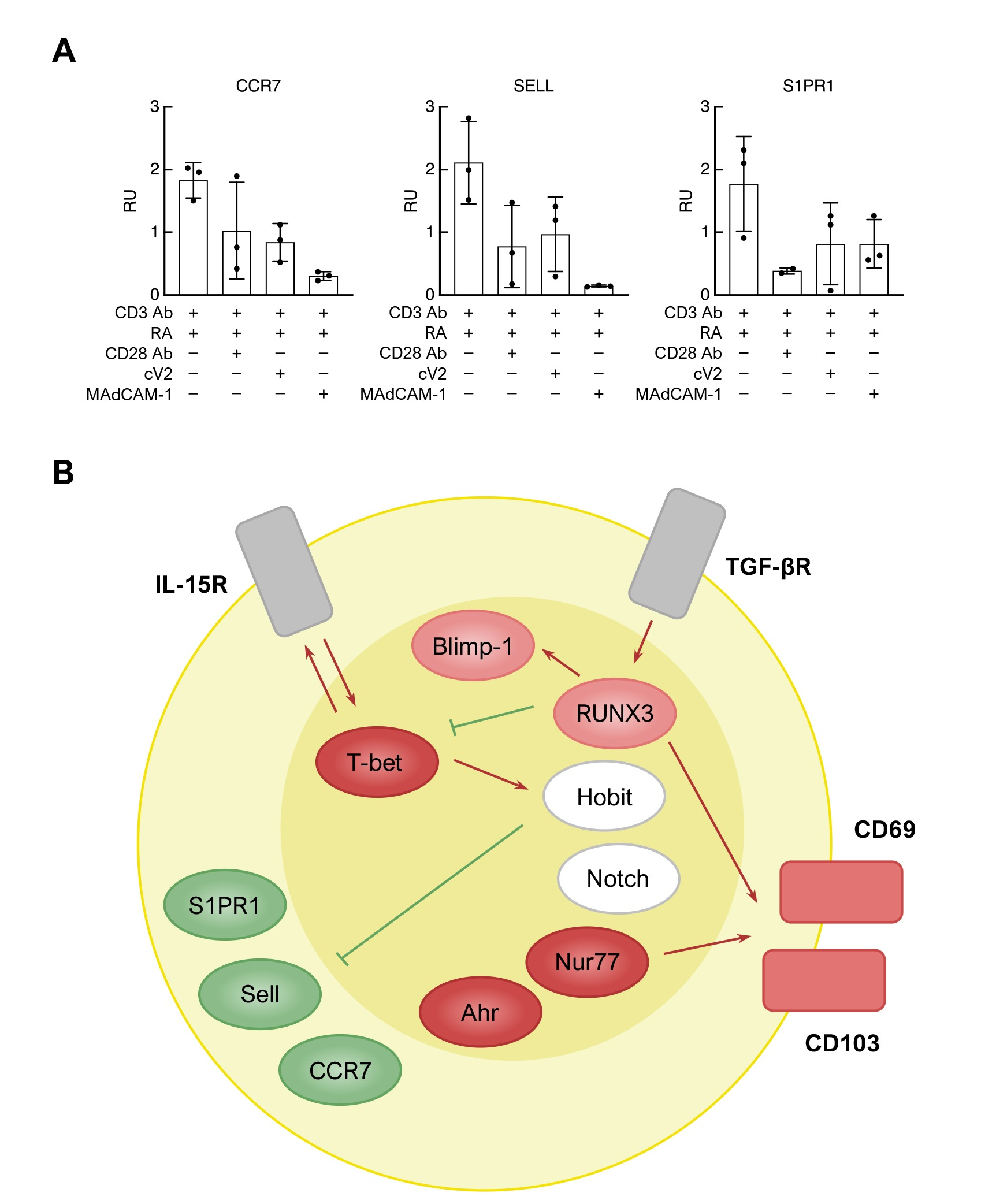

Supplement: S1 Fig — RT-qPCR was performed on RNA derived from CD4+ T cells costimulated with CD28 Ab, MAdCAM-1 and cV2, all in the presence of RA (n = 3). Primers and probes specific to CCR7 (left), SELL (middle) and S1PR1 (right) we employed. Values were normalized to the housekeeping gene TAF1D, and relative units (RU) reported. Error bars indicate standard deviation. (B) The TRM lineage signal translation pathway following MAdCAM-1 + RA treatment is shown. TF genes in the pathway are depicted illustrated as ovals, with a four-color scheme of red, pink, green, and white, which respectively represent up-regulation (> 1.5 fold), moderate up-regulation (1.25–1.5 fold), down-regulation (< -1.5 fold), and no significant changes. Relevant surface receptors are depicted as rectangles. (TIF) [file ppat.1011209.s001.tif]

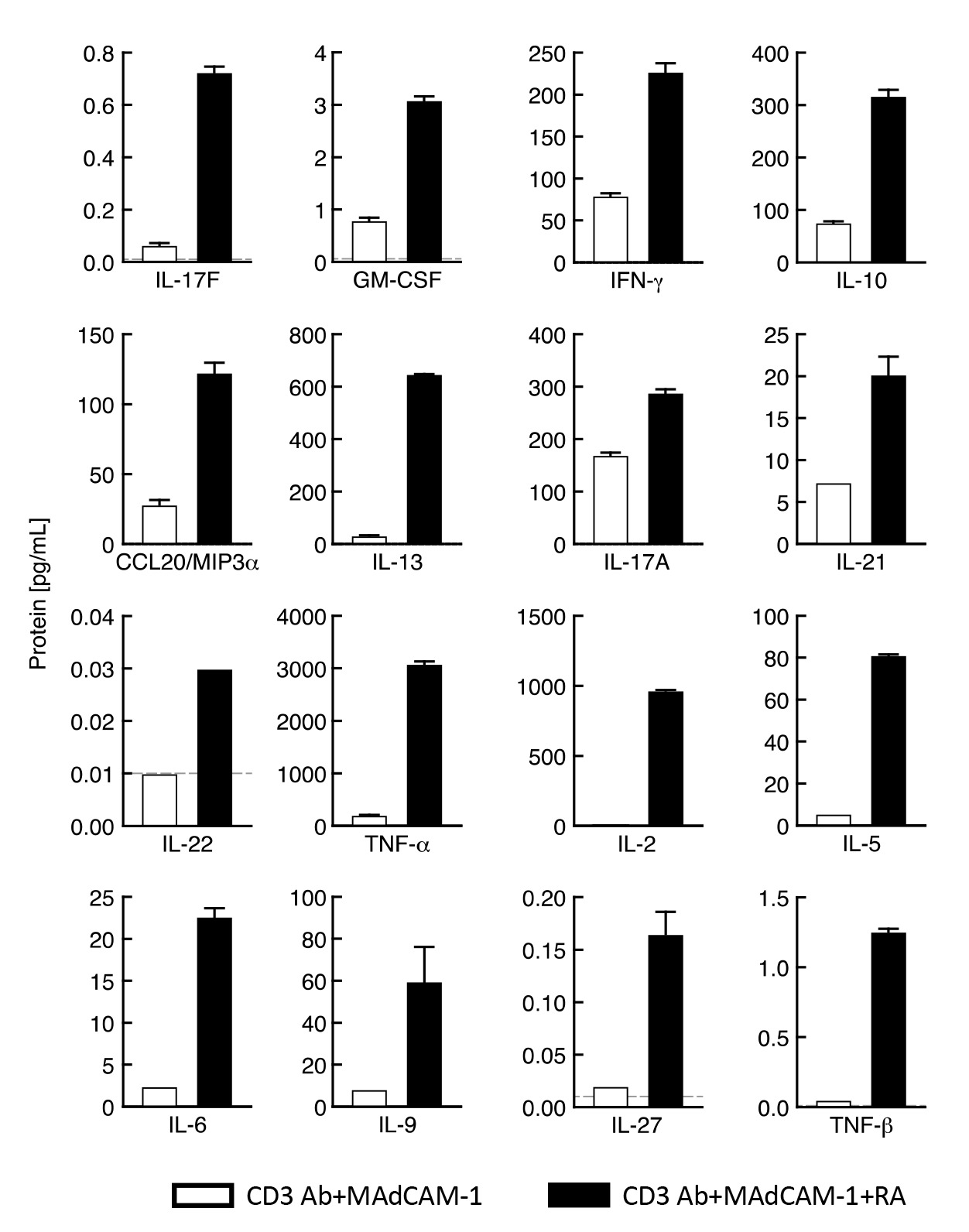

Supplement: S2 Fig — Bead based immunoassay of cytokines in culture supernatants following CD3 Ab + MAdCAM-1 (white bar) or CD3 Ab + MAdCAM-1 + RA (black bar) costimulation of CD4+ T cells. Average cytokine concentration (pg/ml) from 3 independent donors. Error bars indicate standard deviation. Only the analytes that yielded signals above the limit of detection are shown. (TIF) [file ppat.1011209.s002.tif]

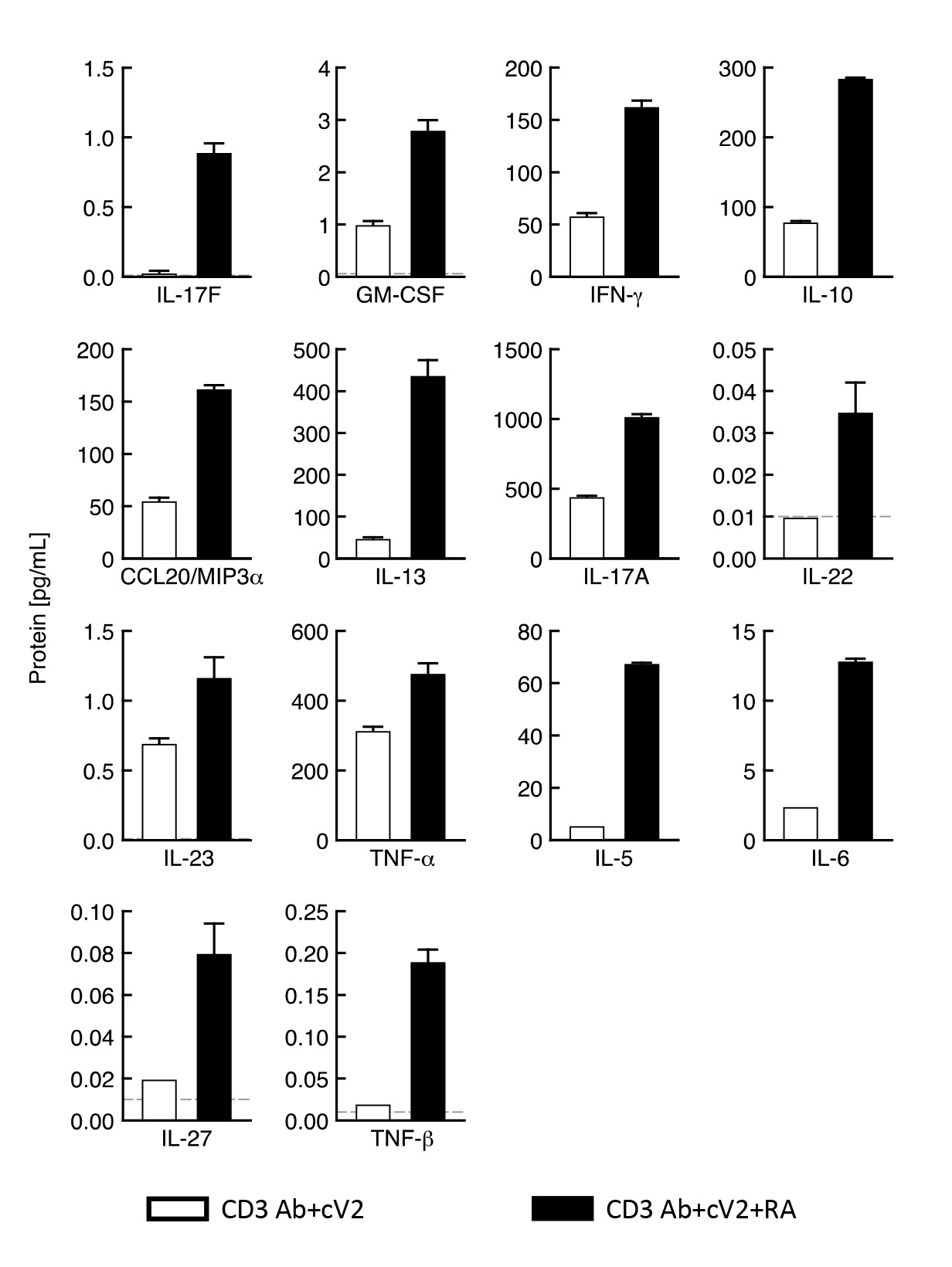

Supplement: S3 Fig — Bead based immunoassay of cytokines in culture supernatants following CD3 Ab + cV2 (white bar) or CD3 Ab + cV2 + RA (black bar) costimulation of CD4+ T cells. Average cytokine concentration (pg/ml) from 3 independent donors. Error bars indicate standard deviation. Only the analytes that yielded signals above the limit of detection are shown. (TIF) [file ppat.1011209.s003.tif]

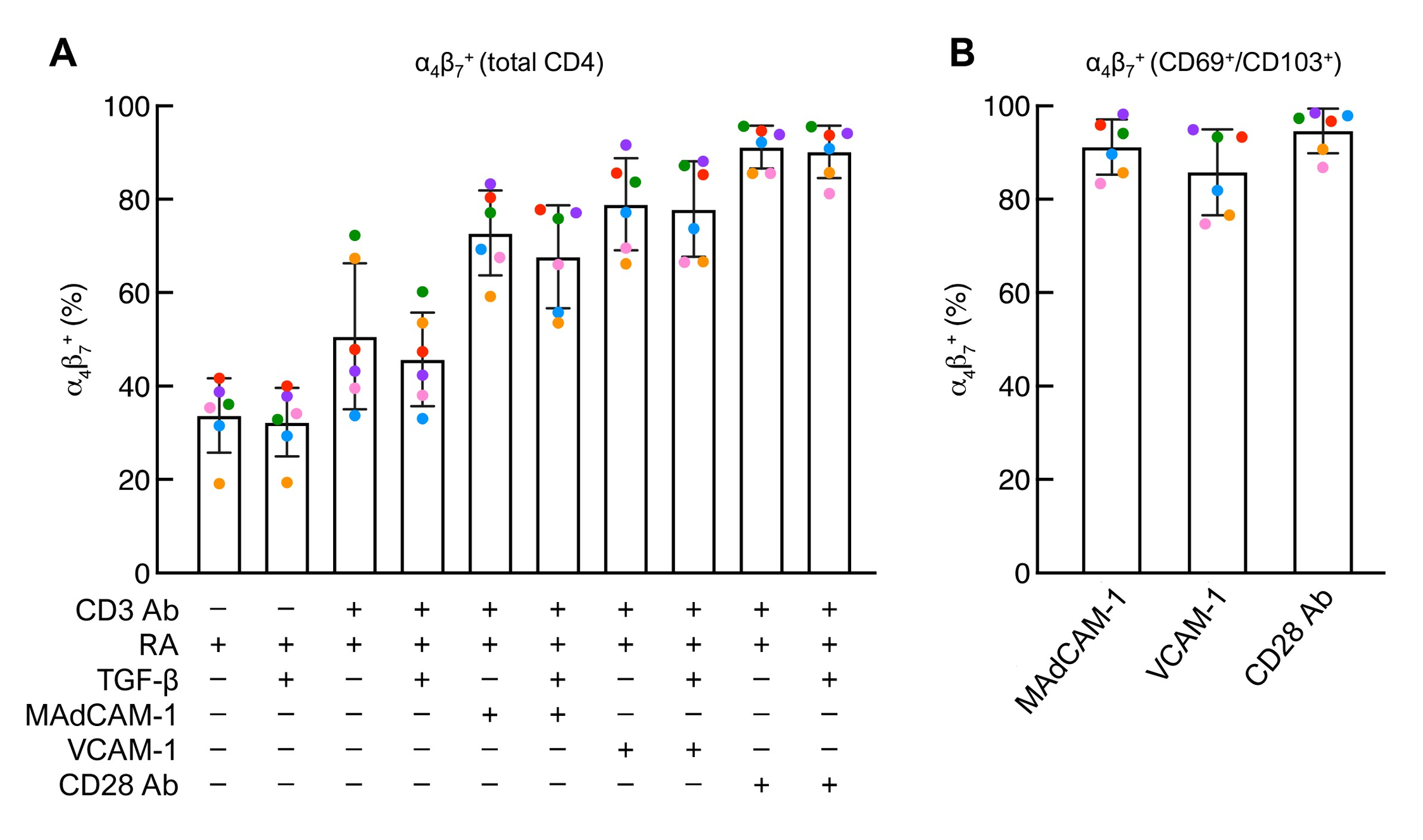

Supplement: S4 Fig — (A) Flow-cytometric analysis of α4β7 expression on total CD4+ T cells without any stimulation or with CD3 Ab + RA, CD3 Ab + MAdCAM-1 + RA, CD3 Ab + VCAM-1 + RA, or CD3 Ab + CD28 Ab + RA in the absence or presence of TGF-β as indicated. (B) α4β7 expression on CD69+/αEβ7+ CD4+ T cells following CD3 Ab + MAdCAM-1, CD3 Ab + VCAM-1, or CD3 Ab + CD28 Ab, as indicated (n = 6). (TIF) [file ppat.1011209.s004.tif]

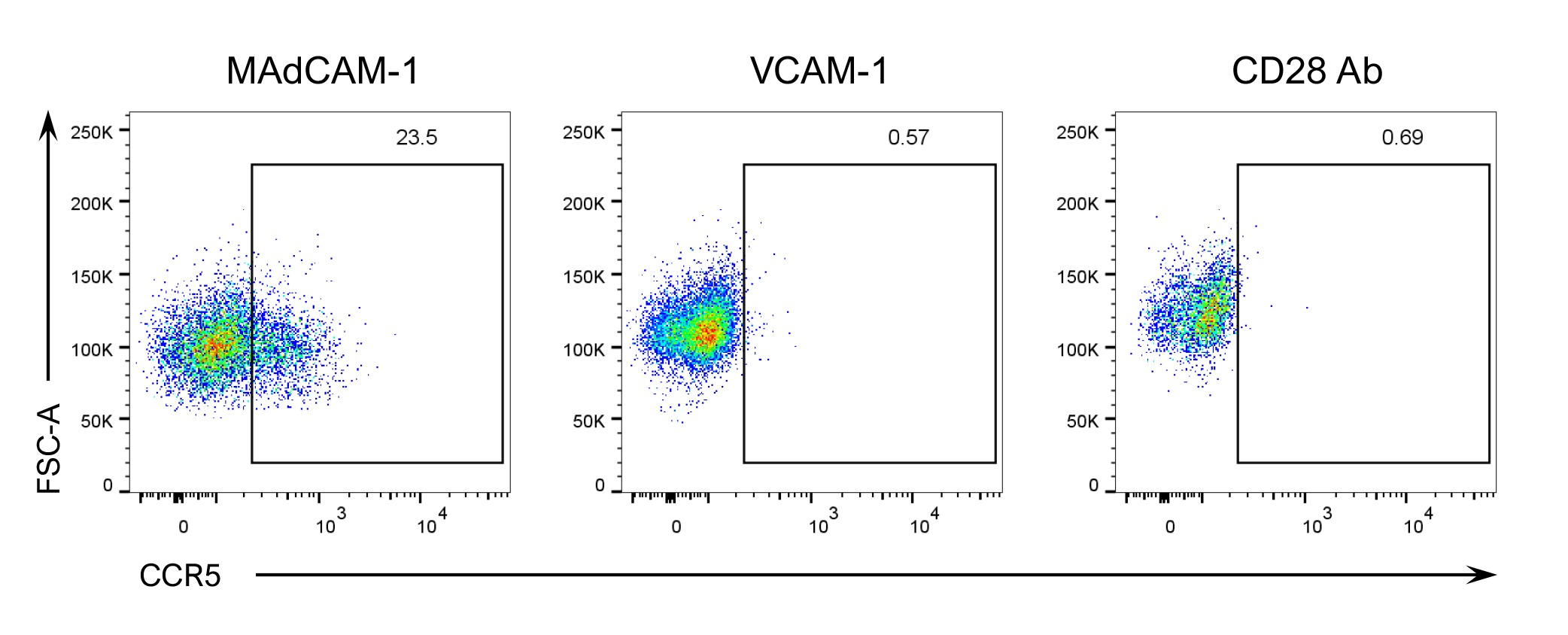

Supplement: S5 Fig — Representative flow cytometry dot plot of CCR5 expression within the CD69+/CD103+ population are shown. Cells stimulated with MAdCAM-1 (left), VCAM-1 (middle), and CD28 Ab (right) are shown. Y-axis: FSC-A, X-axis: CCR5. (TIF) [file ppat.1011209.s005.tif]

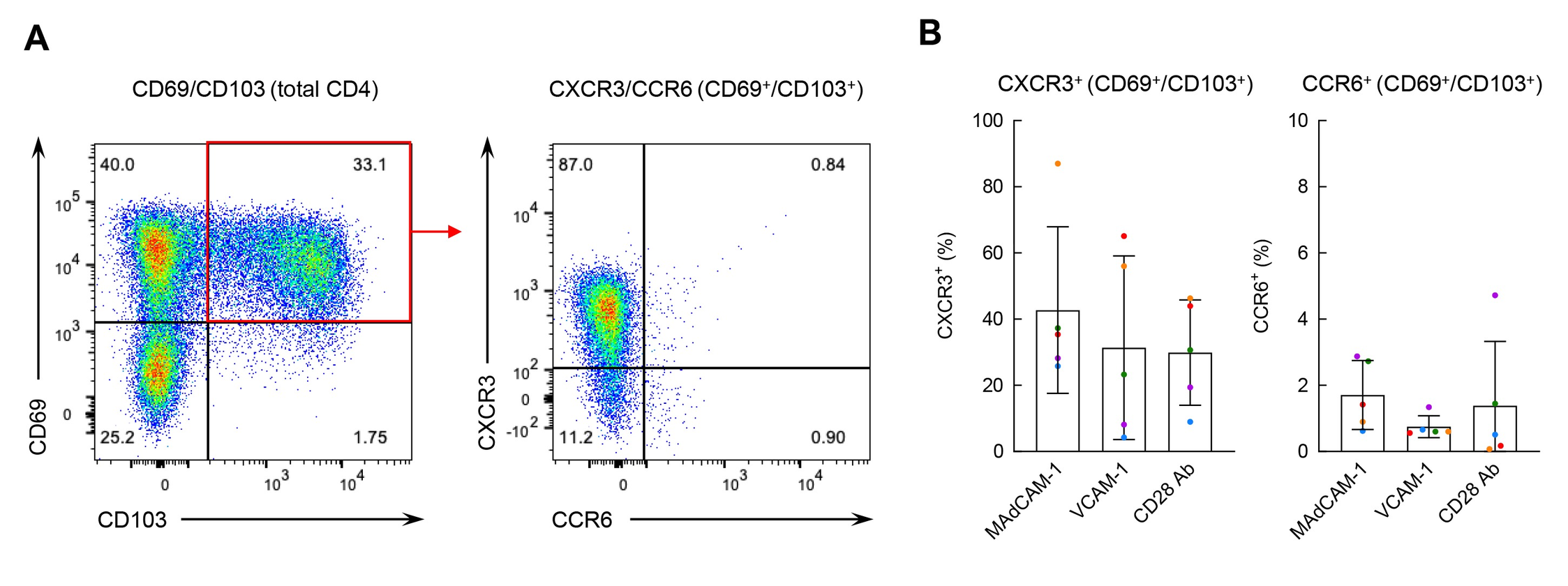

Supplement: S6 Fig — (A) Representative flow cytometric dot plot of MAdCAM-1 + RA + TGF-β treated cells. CD69+/CD103+ cells (left) were stained with CXCR3 (Y-axis) and CCR6 (X-axis). (B) CXCR3 (left) and CCR6 (right) expression in 5 independent donors. (TIF) [file ppat.1011209.s006.tif]

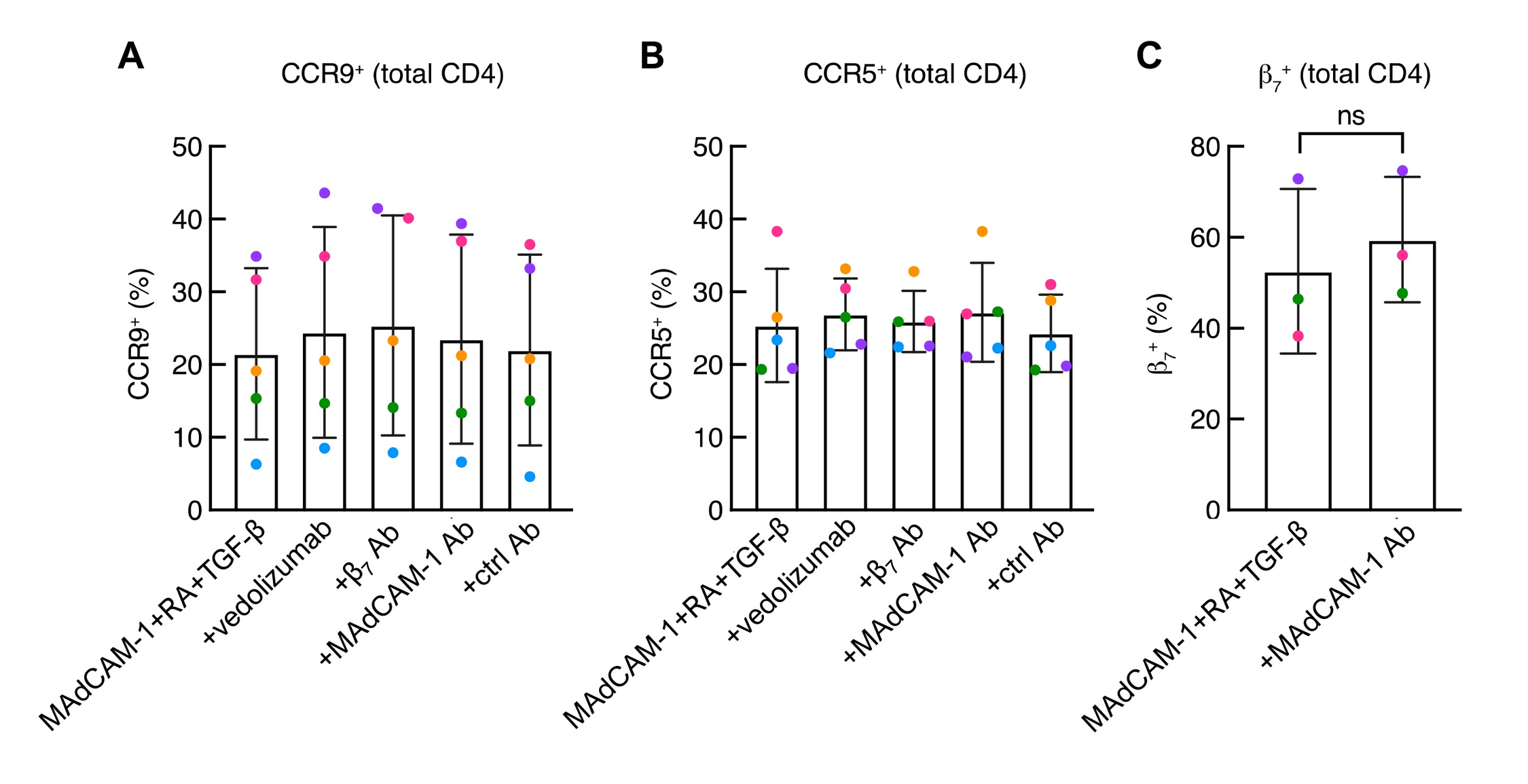

Supplement: S7 Fig — Flow-cytometric analysis of (A) CCR9 (n = 5), (B) CCR5 (n = 5), (C) integrin β7 (n = 3) in the total CD4+ T cell population following MAdCAM-1 + RA + TGF-β costimulation in the absence or presence of vedolizumab, β7 Ab, or MAdCAM-1 Ab, as indicated. (TIF) [file ppat.1011209.s007.tif]

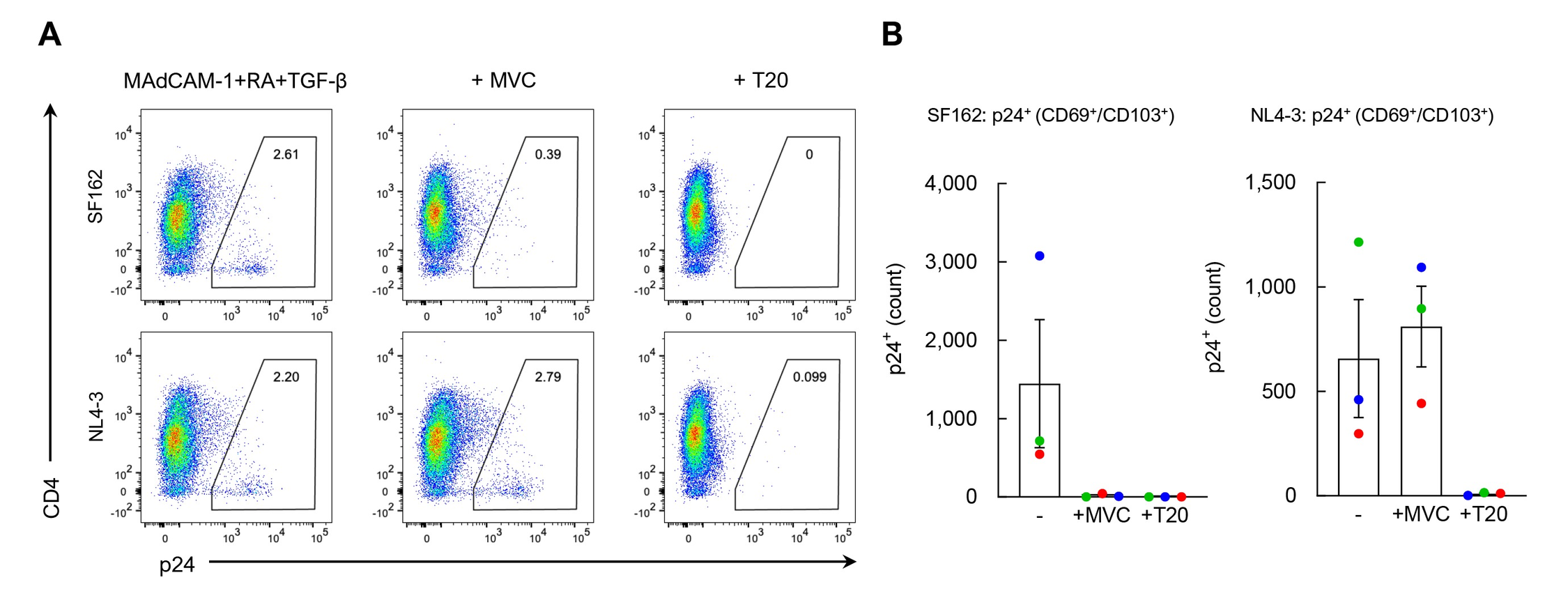

Supplement: S8 Fig — (A) Representative dot plot of an R5 tropic isolate (SF162) (upper row) and an X4-tropic isolate (NL4-3) (lower row) alone (left) or in the presence of maraviroc (middle) or T20 (right) following MAdCAM-1 + RA + TGF-β treatment. Cells shown are CD69+/CD103+. (B) p24 staining, as in A for 3 donors for SF162 (left) and NL4-3 (right). (TIF) [file ppat.1011209.s008.tif]
