# Supplementary material for: MAdCAM-1 costimulation in the presence of retinoic acid and TGF-β promotes HIV infection and differentiation of CD4+ T cells into CCR5+ TRM-like cells
Source: PLoS Pathog. 2023 Mar 10;19(3):e1011209. doi: 10.1371/journal.ppat.1011209 (PMC10032498; doi:10.1371/journal.ppat.1011209)
Supplement: S1 Table — (DOCX) [file ppat.1011209.s009.docx]

**S1 Table. CIBERSORT Reference module of CD4^+^ cell subtypes following MAdCAM-1 + RA costimulation**

| **Gene** | **CTL** | **Naïve** | **Proliferating** | **T_EM_** | **T_RM_** | **Treg** | **T_CM_** | **MAIT** |
| --- | --- | --- | --- | --- | --- | --- | --- | --- |
| ABCB4 | 0.1 | 0 | 0 | 0 | 0 | 0 | 0 | 0.2 |
| ABCB9 | 0.4 | 0.6 | 1.4 | 0.4 | 0.6 | 0.4 | 0.5 | 0.2 |
| ACAP1 | 106.3 | 86.7 | 48.8 | 100 | 88.6 | 86.3 | 98.1 | 97.7 |
| ACHE | 0 | 0 | 0 | 0 | 0 | 0 | 0 | 0 |
| ACP5 | 3.8 | 5.5 | 10.6 | 5.2 | 6.5 | 7.1 | 6 | 2.7 |
| ADAM28 | 0.2 | 0.2 | 0 | 0.4 | 0 | 0.3 | 0.2 | 0.1 |
| ADAMDEC1 | 0 | 0 | 0 | 0 | 0 | 0 | 0 | 0.1 |
| ADRB2 | 32.4 | 0.3 | 0 | 8 | 2.1 | 0.4 | 1.8 | 30.5 |
| AHR | 1.5 | 1.2 | 1.5 | 3.2 | 4.7 | 2 | 3.2 | 1.5 |
| AIF1 | 61 | 118.1 | 43.9 | 46.8 | 46.8 | 57 | 43.6 | 46.1 |
| AIM2 | 5.1 | 0.5 | 8.1 | 4.6 | 5.7 | 1.4 | 4.2 | 3.5 |
| ALOX15 | 0 | 0 | 0 | 0 | 0 | 0 | 0 | 0 |
| ALOX5 | 1.8 | 1.3 | 0 | 2.5 | 4.1 | 1.1 | 3.3 | 1.8 |
| ANGPT4 | 0 | 0 | 0 | 0 | 0 | 0 | 0 | 0 |
| ANKRD55 | 0 | 9.4 | 5.5 | 0.3 | 0 | 3 | 3.2 | 0.1 |
| APOBEC3A | 4.7 | 4 | 2.4 | 4.7 | 4.4 | 2.9 | 3.7 | 4.1 |
| APOBEC3G | 105.7 | 11.8 | 94.7 | 47.3 | 23.3 | 14.2 | 27 | 71.4 |
| APOL3 | 9.9 | 8.7 | 15.2 | 13.1 | 11.7 | 7.5 | 11.7 | 17.4 |
| APOL6 | 10.6 | 5.5 | 7.7 | 8.4 | 7.5 | 6.1 | 7.2 | 8.4 |
| AQP9 | 0.6 | 0.5 | 0.6 | 0.6 | 1.4 | 0.6 | 0.5 | 0.5 |
| ARHGAP22 | 0.1 | 0.1 | 0 | 0.1 | 0 | 0 | 0.1 | 0.1 |
| ARRB1 | 4.1 | 2.6 | 1.6 | 2.5 | 1.9 | 2.5 | 2.2 | 3.2 |
| ASGR1 | 2.1 | 1.5 | 1.1 | 1.7 | 0 | 1 | 1.7 | 1.5 |
| ASGR2 | 0.5 | 0.6 | 2.2 | 0.4 | 0 | 0.3 | 0.6 | 0.5 |
| ATP8B4 | 0.1 | 0.1 | 0.9 | 0.1 | 0 | 0.1 | 0.1 | 0.1 |
| AZU1 | 0.1 | 0.1 | 0 | 0.5 | 0 | 0.3 | 0.2 | 0.5 |
| BACH2 | 0.8 | 5.5 | 0.9 | 2.6 | 4.1 | 2.6 | 2.7 | 1.6 |
| BANK1 | 0.8 | 0.6 | 0.5 | 0.9 | 0.6 | 0.6 | 0.6 | 1.4 |
| BARX2 | 0 | 0 | 0 | 0 | 0 | 0 | 0 | 0 |
| BATF | 59.7 | 14.1 | 69.3 | 26 | 26.9 | 32.8 | 26.8 | 24.1 |
| BCL11B | 19 | 35.3 | 12.2 | 24.9 | 26.3 | 25.8 | 28.6 | 24 |
| BCL2 | 14.3 | 21.6 | 3.7 | 26.7 | 32.3 | 27 | 29.9 | 26.1 |
| BCL2A1 | 46 | 28.3 | 5.5 | 39.1 | 35.8 | 29.7 | 33.4 | 29.7 |
| BCL6 | 3.1 | 1 | 0.5 | 2.6 | 1.7 | 1.6 | 1.9 | 6 |
| BCL7A | 1.7 | 2.4 | 3.3 | 1.1 | 1.1 | 0.5 | 1.8 | 0.4 |
| BEND5 | 0.4 | 4.7 | 1.3 | 0.3 | 0 | 2.5 | 0.8 | 0.2 |
| BFSP1 | 3 | 0.1 | 2.4 | 0.5 | 1 | 0 | 0.6 | 0.2 |
| BHLHE41 | 0 | 0 | 0 | 0.1 | 0 | 0 | 0 | 0 |
| BIRC3 | 10.5 | 18.1 | 5 | 23.6 | 33.6 | 51.7 | 32.3 | 10 |
| BLK | 0.5 | 0.6 | 0.7 | 1.1 | 2.5 | 0.5 | 1.2 | 16.2 |
| BMP2K | 1.6 | 1.8 | 3.2 | 1 | 2.8 | 1.1 | 1 | 1 |
| BPI | 0.1 | 0 | 0 | 0 | 0 | 0.1 | 0 | 0 |
| BRAF | 18.1 | 14.1 | 9.4 | 16.8 | 20.1 | 13 | 16.5 | 18 |
| BRSK2 | 0.1 | 0.1 | 0 | 0.1 | 0 | 0.1 | 0.1 | 0 |
| BST1 | 1.6 | 1.3 | 1.2 | 1.6 | 0 | 1 | 1.3 | 1.4 |
| BTNL8 | 0 | 0 | 0 | 0 | 0 | 0 | 0 | 0 |
| C11orf80 | 1.5 | 1.3 | 7.4 | 2.3 | 1.8 | 2.2 | 2.3 | 1.9 |
| C1orf54 | 2.6 | 5.1 | 6.8 | 8.4 | 16.6 | 4 | 8.5 | 3.5 |
| C3AR1 | 4.2 | 0.5 | 0.9 | 3 | 2.2 | 0.5 | 1.8 | 4.8 |
| C5AR1 | 3.1 | 1.8 | 2.2 | 2.4 | 5.4 | 2.2 | 1.7 | 1.7 |
| CA8 | 0 | 0 | 0 | 0 | 0 | 0.1 | 0 | 0 |
| CAMP | 0.2 | 0.1 | 0 | 0.1 | 0 | 0 | 0.1 | 0.1 |
| CASP5 | 0.1 | 0.1 | 0 | 0.3 | 0 | 0.2 | 0.1 | 0.4 |
| CCDC102B | 2.5 | 3.3 | 1.8 | 1.9 | 1.5 | 2.4 | 2.4 | 1.6 |
| CCL18 | 0 | 0 | 0 | 0 | 0 | 0 | 0 | 0 |
| CCL19 | 0 | 0 | 0 | 0 | 0 | 0 | 0 | 0 |
| CCL2 | 0.3 | 0 | 0 | 0.1 | 0 | 0 | 0.1 | 0 |
| CCL20 | 4 | 2.5 | 2 | 6.9 | 15 | 1.9 | 6 | 11 |
| CCL22 | 0 | 0 | 0 | 0 | 0 | 0 | 0 | 0 |
| CCL23 | 0.2 | 0 | 0 | 0 | 0 | 0.1 | 0 | 0 |
| CCL3 | 90.7 | 42.4 | 23.7 | 51 | 47.6 | 33.9 | 38 | 55.2 |
| CCL4 | 652.6 | 50.7 | 38.5 | 84.7 | 44.1 | 37.2 | 48.4 | 187.5 |
| CCL5 | 3442.8 | 59.6 | 239.1 | 1095.9 | 1270.3 | 55.4 | 139.3 | 2027.5 |
| CCL7 | 0 | 0 | 0 | 0 | 0 | 0 | 0 | 0 |
| CCL8 | 0 | 0 | 0 | 0 | 0 | 0 | 0 | 0 |
| CCND2 | 18.1 | 11.3 | 20.1 | 21.1 | 23.4 | 12 | 22.1 | 22.2 |
| CCR10 | 1 | 0.5 | 8.3 | 1.6 | 0 | 1.4 | 2.7 | 0.6 |
| CCR2 | 1.4 | 0.2 | 4.5 | 6 | 1.6 | 0.6 | 2.9 | 9 |
| CCR3 | 0.1 | 0 | 0.9 | 0 | 0 | 0.1 | 0.1 | 0.1 |
| CCR4 | 0.4 | 0.6 | 8.3 | 2.7 | 1.3 | 5.7 | 10.6 | 0.6 |
| CCR5 | 2.5 | 0.1 | 7.1 | 2 | 0.6 | 0.1 | 0.4 | 4.9 |
| CCR6 | 1.2 | 0.3 | 3.5 | 15.5 | 25 | 2.6 | 15.2 | 22.1 |
| CCR7 | 1.9 | 145.9 | 22.2 | 18 | 15.6 | 73.5 | 53.3 | 4.2 |
| CD160 | 5.2 | 1.1 | 8.8 | 1.1 | 2.7 | 1.1 | 1 | 14.7 |
| CD180 | 0.1 | 0.1 | 0 | 0.1 | 0.8 | 0.1 | 0.1 | 0.1 |
| CD19 | 0 | 0.2 | 0 | 0.2 | 0 | 0.3 | 0.2 | 0.2 |
| CD1A | 0 | 0 | 0 | 0 | 0 | 0.1 | 0 | 0 |
| CD1B | 0 | 0 | 0 | 0 | 0 | 0 | 0 | 0 |
| CD1C | 0.1 | 0.2 | 0 | 0.3 | 0.8 | 0.2 | 0.2 | 0.3 |
| CD1D | 0.4 | 0.3 | 0.5 | 0.4 | 0.6 | 0.4 | 0.4 | 0.3 |
| CD1E | 0 | 0 | 0 | 0 | 0 | 0 | 0 | 0.1 |
| CD2 | 662.6 | 190.1 | 381.1 | 398.1 | 334.1 | 215 | 327.7 | 287.7 |
| CD209 | 0 | 0 | 0 | 0 | 0 | 0 | 0 | 0 |
| CD22 | 0.3 | 0.3 | 0.5 | 0.3 | 0 | 0.3 | 0.3 | 0.2 |
| CD244 | 5.2 | 1.1 | 0 | 0.8 | 2.6 | 0.3 | 0.6 | 5.5 |
| CD247 | 219.1 | 108.9 | 153.6 | 104.4 | 91.8 | 120.9 | 103.4 | 126.5 |
| CD27 | 5.9 | 151.8 | 191.6 | 99 | 65.6 | 182.8 | 128.8 | 90.8 |
| CD28 | 2.6 | 14.3 | 55.3 | 22.4 | 17.3 | 14.6 | 23.2 | 17.1 |
| CD300A | 36.6 | 2.2 | 11.9 | 12.3 | 13.4 | 1.4 | 7.3 | 12.8 |
| CD33 | 1.3 | 1.2 | 1.2 | 1.7 | 0 | 0.8 | 1.3 | 1.4 |
| CD37 | 400.7 | 309 | 64.9 | 304.6 | 307 | 337.6 | 312.4 | 324.1 |
| CD38 | 2.2 | 7.5 | 97 | 1.4 | 0 | 1.4 | 2.8 | 1 |
| CD3D | 1444.4 | 885.9 | 1445.8 | 916 | 718.6 | 955 | 929.1 | 1098.7 |
| CD3E | 574.4 | 375.6 | 366.3 | 414.6 | 405.1 | 371.5 | 380.5 | 492.1 |
| CD3G | 786.6 | 421.2 | 587.8 | 413.7 | 494.6 | 432.2 | 409.1 | 389.5 |
| CD4 | 50.2 | 28.3 | 39.5 | 38.5 | 34.1 | 25.8 | 36.3 | 5.3 |
| CD40 | 2 | 1.9 | 0 | 1.4 | 0 | 1.8 | 1.4 | 1.3 |
| CD40LG | 49.1 | 28.5 | 15 | 49.4 | 51.9 | 8.7 | 46.4 | 36 |
| CD44 | 56.1 | 42 | 28.9 | 52.6 | 49.2 | 50.7 | 52 | 53.4 |
| CD5 | 31.5 | 13.7 | 44.8 | 16.2 | 16.8 | 13.1 | 21.5 | 8.5 |
| CD6 | 57 | 29.3 | 66.6 | 38.3 | 41.9 | 17.8 | 38.1 | 27.9 |
| CD68 | 7.2 | 3.8 | 5.6 | 4.5 | 3.4 | 3.4 | 3.6 | 4.9 |
| CD69 | 136.7 | 117.8 | 50.3 | 319 | 319.2 | 131.9 | 253.7 | 514.4 |
| CD7 | 57.4 | 195 | 111.3 | 110.6 | 123.5 | 123.7 | 116.9 | 210.3 |
| CD70 | 3.4 | 0.4 | 30.1 | 5.6 | 2.3 | 2.3 | 3.1 | 6.9 |
| CD72 | 2.3 | 0.6 | 3.3 | 1.4 | 1.4 | 1.1 | 1 | 2.9 |
| CD79A | 2.2 | 3.8 | 2.7 | 2.7 | 1.7 | 5.6 | 4.3 | 2.6 |
| CD79B | 15.4 | 13.2 | 32.7 | 22.2 | 20 | 16.6 | 23.9 | 26.2 |
| CD80 | 0 | 0 | 0 | 0.2 | 0.8 | 0.1 | 0.2 | 0.1 |
| CD86 | 0.6 | 0.5 | 0.6 | 0.6 | 0.8 | 0.2 | 0.6 | 0.5 |
| CD8A | 8.6 | 1.8 | 60.3 | 2.6 | 4.8 | 1.8 | 2 | 93 |
| CD8B | 3.1 | 4.5 | 73.2 | 2.9 | 2.9 | 4.9 | 2.9 | 35.6 |
| CD96 | 33.4 | 21.2 | 19.3 | 34.7 | 54.9 | 15.5 | 27.5 | 39.5 |
| CDA | 2.6 | 2.7 | 0 | 2.5 | 0 | 1.7 | 2.8 | 3.3 |
| CDC25A | 0 | 0 | 5.1 | 0 | 0 | 0 | 0 | 0 |
| CDH1 | 0 | 0 | 0 | 0 | 0 | 0 | 0 | 0 |
| CDK6 | 3 | 3.8 | 2.1 | 4.2 | 5.4 | 1.6 | 4.2 | 4.5 |
| CEACAM3 | 0.2 | 0.1 | 0 | 0.1 | 0 | 0.3 | 0.2 | 0 |
| CEMP1 | 0 | 0 | 0 | 0 | 0 | 0 | 0.1 | 0 |
| CFP | 4.2 | 10.1 | 4 | 7.5 | 9.9 | 7.4 | 9.5 | 4.1 |
| CHI3L1 | 0 | 0 | 0 | 0 | 0 | 0 | 0 | 0 |
| CHI3L2 | 4.9 | 22.6 | 11.5 | 3.4 | 1.4 | 2 | 3.7 | 2.6 |
| CHN2 | 1 | 0.2 | 0 | 0.7 | 1.2 | 0.1 | 0.4 | 1.6 |
| CHST7 | 2.7 | 0.9 | 8.9 | 2.5 | 8.2 | 4.3 | 3.3 | 2.7 |
| CLC | 0.2 | 0.3 | 0 | 0.1 | 0 | 0.3 | 0.4 | 0 |
| CLEC10A | 0.7 | 0.5 | 0 | 0.5 | 0 | 0.4 | 0.5 | 0.6 |
| CLEC2D | 90.4 | 54.7 | 56.6 | 64 | 50.1 | 46.8 | 55.3 | 49.7 |
| CLEC4A | 3 | 1.9 | 1.3 | 2.5 | 3.3 | 1.7 | 2.2 | 2.6 |
| CLEC7A | 3.6 | 3.1 | 3.3 | 4 | 6.5 | 2.9 | 3.1 | 3.7 |
| CLIC2 | 0.2 | 0.1 | 0.6 | 0.1 | 0 | 0.1 | 0.1 | 0 |
| COL8A2 | 0 | 0 | 0 | 0 | 0 | 0 | 0 | 0 |
| COLQ | 2.5 | 0.2 | 0.6 | 3.5 | 3.4 | 0.2 | 1.5 | 12.3 |
| CPA3 | 0 | 0 | 0 | 0 | 0 | 0 | 0 | 0 |
| CR2 | 0.1 | 0.5 | 0 | 0.2 | 0 | 0 | 0.4 | 0.2 |
| CREB5 | 0.4 | 0.2 | 0 | 0.3 | 0.2 | 0.1 | 0.2 | 0.2 |
| CRTAM | 2.2 | 2.3 | 5 | 2.1 | 0 | 0.9 | 0.4 | 4.8 |
| CRYBB1 | 0 | 0 | 0 | 0 | 0 | 0 | 0 | 0.1 |
| CSF1 | 0.2 | 0.1 | 1.7 | 1.8 | 1.4 | 0.9 | 1.6 | 0.9 |
| CSF2 | 0 | 0 | 0 | 0 | 0 | 0 | 0 | 0.1 |
| CSF3R | 3.9 | 3 | 2.2 | 3.5 | 1.4 | 3.4 | 2.9 | 3.4 |
| CST7 | 996.3 | 13.4 | 83.3 | 183.2 | 106.8 | 33.3 | 35.7 | 624.6 |
| CTLA4 | 8.7 | 3.3 | 63.6 | 7.3 | 2.1 | 30.6 | 12.4 | 1.1 |
| CTSG | 0.7 | 0 | 0 | 0 | 0 | 0 | 0 | 0 |
| CTSW | 149 | 15.4 | 42.5 | 64.5 | 39.1 | 11.1 | 31.1 | 323.7 |
| CX3CR1 | 55.1 | 0.8 | 13.9 | 2.9 | 2 | 0.6 | 1.1 | 1.4 |
| CXCL10 | 0.1 | 0.4 | 0 | 0.3 | 0 | 0.2 | 0.3 | 0.3 |
| CXCL11 | 0 | 0 | 0 | 0 | 0 | 0 | 0 | 0 |
| CXCL16 | 0.8 | 0.9 | 0.7 | 1.3 | 0 | 0.5 | 1.1 | 1.4 |
| CXCL3 | 1.9 | 1.1 | 1.5 | 1.6 | 0 | 0.9 | 1 | 1.3 |
| CXCL5 | 0.1 | 0.1 | 0 | 0.1 | 0 | 0 | 0.1 | 0.1 |
| CXCL9 | 0 | 0 | 0 | 0 | 0 | 0 | 0 | 0 |
| CXCR3 | 15.9 | 0.5 | 107.9 | 18.4 | 18.1 | 3.9 | 11 | 4.4 |
| CXCR5 | 0.7 | 0.9 | 1.2 | 0.8 | 1.5 | 2.1 | 2.9 | 0.7 |
| CXCR6 | 3 | 0.1 | 11.5 | 4.9 | 1.1 | 0.4 | 0.9 | 25.2 |
| CXorf57 | 0.8 | 0.7 | 1.8 | 0.9 | 0.5 | 0.8 | 1.2 | 0.6 |
| CYP27A1 | 0.3 | 0.3 | 1.5 | 0.3 | 0 | 0.1 | 0.2 | 0.4 |
| CYP27B1 | 0 | 0 | 1.4 | 0.1 | 0 | 0 | 0.1 | 0.2 |
| DACH1 | 0 | 0.2 | 0 | 0 | 0.4 | 0 | 0 | 0 |
| DAPK2 | 3.8 | 0.1 | 0 | 0.8 | 6.4 | 0.1 | 0.3 | 1.1 |
| DENND5B | 0 | 0 | 0 | 0 | 0 | 0 | 0 | 0 |
| DEPDC5 | 1.8 | 1.4 | 2.6 | 1.5 | 1.2 | 1.4 | 1.6 | 1.7 |
| DGKA | 12.7 | 48.9 | 32.3 | 36.7 | 49.6 | 45.6 | 45.4 | 20.4 |
| DHRS11 | 1.7 | 1.7 | 1.1 | 1.7 | 0 | 1.4 | 1.7 | 1.8 |
| DHX58 | 2.7 | 3 | 4 | 3 | 5.7 | 2.2 | 2.9 | 3 |
| DPEP2 | 8.9 | 15.5 | 1 | 13.6 | 13.6 | 13.5 | 14.8 | 7.9 |
| DPP4 | 1.4 | 8 | 7.1 | 20.4 | 22.3 | 4.8 | 12.4 | 27.1 |
| DSC1 | 0 | 1 | 0.4 | 0 | 0 | 0 | 0.1 | 0 |
| DUSP2 | 279.4 | 25.4 | 60.3 | 314.1 | 111.7 | 41.8 | 78.1 | 1126 |
| EAF2 | 5.8 | 7.4 | 13.5 | 8 | 8.3 | 7.6 | 8 | 9.6 |
| EBI3 | 0 | 0 | 1.5 | 0 | 0 | 0 | 0 | 0 |
| EFNA5 | 0.3 | 0 | 0 | 0 | 0 | 0 | 0 | 0 |
| EGR2 | 0.4 | 0.2 | 0.6 | 0.3 | 0 | 0.2 | 0.2 | 0.2 |
| ELANE | 0 | 0 | 0 | 0.1 | 0 | 0.1 | 0 | 0.2 |
| ELOVL7 | 0 | 0 | 0 | 0 | 0 | 0.1 | 0 | 0 |
| EOMES | 13.4 | 0.5 | 4.4 | 8.5 | 0.8 | 0.4 | 0.8 | 24.1 |
| EPB41 | 35.9 | 38.1 | 45.5 | 40.9 | 40.9 | 44.4 | 38.4 | 32.6 |
| EPHA1 | 0 | 1.7 | 0 | 0.4 | 0.6 | 1.1 | 1.3 | 0.1 |
| EPN2 | 1.1 | 1 | 1.1 | 1.6 | 1.3 | 1.1 | 1.2 | 0.8 |
| ETS1 | 114.7 | 129.5 | 140.9 | 131.8 | 135 | 132.8 | 123 | 123.2 |
| ETV3 | 9.4 | 4.2 | 5.2 | 11.2 | 13.7 | 4.4 | 7.9 | 16 |
| FAM124B | 0 | 0 | 0.7 | 0.2 | 0 | 0.3 | 0.2 | 0 |
| FAM174B | 0 | 0.6 | 0.7 | 0.2 | 0 | 0.6 | 0.4 | 0.1 |
| FAS | 9.9 | 1.3 | 26.3 | 10.8 | 12.6 | 7 | 10.2 | 8.4 |
| FASLG | 4.8 | 0.1 | 0 | 0.5 | 0 | 0 | 0.3 | 3.3 |
| FBXL8 | 3.4 | 2.9 | 7.5 | 11.2 | 14.5 | 2.2 | 13.6 | 2.7 |
| FCER1A | 0.1 | 0.6 | 0 | 0.8 | 0 | 0.5 | 0.6 | 0.8 |
| FCER2 | 0.3 | 0.3 | 0 | 0.3 | 0 | 0.2 | 0.3 | 0 |
| FCGR2B | 0.7 | 0.3 | 0 | 0.5 | 0 | 0.2 | 0.4 | 0.3 |
| FCGR3B | 0.1 | 0 | 0 | 0.1 | 0 | 0 | 0 | 0 |
| FCN1 | 50.6 | 28.4 | 52.2 | 33.8 | 23 | 30.9 | 26.8 | 37.2 |
| FCRL2 | 0.1 | 0.1 | 0 | 0.2 | 0 | 0.5 | 0.2 | 0.1 |
| FES | 0.6 | 0.6 | 0.6 | 1 | 1.5 | 0.7 | 0.8 | 0.9 |
| FFAR2 | 1.3 | 0.8 | 0 | 1 | 2.1 | 0.5 | 0.9 | 1.6 |
| FLT3LG | 36.4 | 65.3 | 36.5 | 70.4 | 81.5 | 45.3 | 75.1 | 63.7 |
| FLVCR2 | 0.3 | 0.2 | 0 | 0.2 | 0 | 0.2 | 0.2 | 0.2 |
| FOSB | 3.5 | 1.9 | 0 | 4.4 | 3.9 | 2.5 | 3.5 | 5.7 |
| FOXP3 | 0.2 | 0.4 | 13 | 0.8 | 0.9 | 33.6 | 1.1 | 0.3 |
| FPR1 | 2.8 | 2.6 | 2.6 | 3.3 | 1.6 | 2.2 | 2.6 | 3.6 |
| FPR2 | 0.4 | 0.2 | 0 | 0.5 | 0 | 0.2 | 0.3 | 0.2 |
| FPR3 | 0 | 0 | 0 | 0 | 0 | 0 | 0 | 0 |
| FRMD4A | 0.1 | 0.1 | 0.3 | 0.1 | 0 | 0.1 | 0.1 | 0.1 |
| FRMD8 | 6.5 | 3.3 | 6 | 4.8 | 5.1 | 3.9 | 4.4 | 5.4 |
| FZD2 | 0.4 | 0.1 | 0 | 0 | 0 | 0.1 | 0.1 | 0.2 |
| FZD3 | 0.9 | 1.4 | 0.4 | 1.3 | 0.5 | 2.2 | 1.6 | 1.5 |
| GAL3ST4 | 0.1 | 2.2 | 0.7 | 0.4 | 0 | 1.5 | 0.9 | 0.1 |
| GFI1 | 7.9 | 0.8 | 3.1 | 3.1 | 5.3 | 1.1 | 1.3 | 4.2 |
| GGT5 | 0 | 0 | 0 | 0 | 0 | 0 | 0 | 0 |
| GIPR | 0.7 | 0.4 | 0 | 0.4 | 0 | 0.8 | 0.3 | 0.8 |
| GNG7 | 0.4 | 1.4 | 0 | 0.8 | 1 | 1 | 1.2 | 1 |
| GNLY | 5286.3 | 60.4 | 62.6 | 252 | 57.6 | 59.5 | 122.5 | 1218.4 |
| GPC4 | 0 | 0 | 0 | 0 | 0 | 0 | 0 | 0 |
| GPR171 | 21.9 | 14.3 | 18.2 | 36.3 | 64.8 | 11.8 | 27.6 | 76.1 |
| GPR18 | 4.2 | 8.2 | 10.4 | 4.8 | 7.6 | 4.1 | 5.5 | 8.3 |
| GPR183 | 19.4 | 61.6 | 70.4 | 130 | 88.9 | 53 | 154.6 | 49.8 |
| GPR19 | 0.1 | 0.2 | 5.9 | 0.3 | 1.2 | 1.2 | 0.5 | 0.2 |
| GPR25 | 0.1 | 0 | 4.7 | 0 | 0 | 0 | 0.1 | 0.1 |
| GPR65 | 99.2 | 15.9 | 12.8 | 70.6 | 65.1 | 22.2 | 45.4 | 174.5 |
| GRAP2 | 35.7 | 30.1 | 48.8 | 28 | 28.6 | 17.6 | 28.6 | 30.9 |
| GYPE | 0 | 0.2 | 2 | 0.1 | 0 | 0.1 | 0.1 | 0 |
| GZMA | 1506.7 | 19.6 | 362.1 | 539.6 | 105.9 | 19.8 | 84.2 | 1206.7 |
| GZMB | 655.7 | 11.3 | 48.2 | 16.6 | 9.1 | 8.8 | 11.8 | 15.1 |
| GZMH | 1559.8 | 8 | 97.1 | 31.7 | 0 | 7.1 | 9.4 | 23.4 |
| GZMK | 45.8 | 5.8 | 300.9 | 617.2 | 36.3 | 19.4 | 29.2 | 1144 |
| GZMM | 544.1 | 110.7 | 110.6 | 180.1 | 140.3 | 66.7 | 94.5 | 320.6 |
| HAL | 0.1 | 0.1 | 0 | 0.2 | 0 | 0.1 | 0.2 | 0 |
| HAVCR2 | 1.7 | 1 | 6.7 | 1.3 | 0 | 0.5 | 1 | 0.7 |
| HCK | 2.4 | 2.1 | 3.3 | 2.1 | 3 | 1.2 | 1.7 | 1.8 |
| HDC | 0.1 | 0 | 0.7 | 0 | 0 | 0 | 0 | 0 |
| HESX1 | 0.3 | 0.1 | 0 | 0.1 | 0 | 0.1 | 0.1 | 0 |
| HHEX | 2.9 | 0.8 | 0 | 1.1 | 2.4 | 0.9 | 1 | 1.1 |
| HIC1 | 0.3 | 0.3 | 2.2 | 2 | 4 | 0.2 | 0.7 | 1.2 |
| HIF1A | 13.6 | 14.7 | 11.1 | 14 | 12.5 | 9.7 | 13 | 12.5 |
| HK3 | 0.6 | 0.6 | 0 | 0.5 | 1.4 | 0.7 | 0.6 | 0.7 |
| HLA-DOB | 1.3 | 0.8 | 2.5 | 1.1 | 0 | 0.6 | 0.8 | 0.7 |
| HLA-DQA1 | 13 | 3.4 | 5.6 | 3.7 | 2.7 | 5.3 | 3.8 | 3.5 |
| HLA-DRA | 135.3 | 56.5 | 112.6 | 69.8 | 67.5 | 71.5 | 53.9 | 80.9 |
| HNMT | 0.9 | 0.7 | 0.5 | 0.6 | 0.6 | 0.5 | 0.6 | 0.7 |
| HOXA1 | 0.3 | 0.1 | 0 | 0.1 | 0 | 0.2 | 0.1 | 0.2 |
| HPSE | 1.8 | 1.8 | 9.6 | 2.6 | 0 | 2.7 | 2.7 | 1.9 |
| HRH1 | 0 | 0 | 0 | 0 | 0 | 0 | 0 | 0 |
| HSPA6 | 0.5 | 0.1 | 0 | 0.3 | 0 | 0.1 | 0.2 | 0.3 |
| HTR2B | 0 | 0 | 0.8 | 0.1 | 0.9 | 0 | 0 | 0 |
| ICA1 | 0.7 | 0.6 | 21.7 | 1 | 3.5 | 12.1 | 2.9 | 0.7 |
| ICOS | 4.7 | 8.7 | 45.5 | 8.8 | 8.1 | 9.1 | 13.3 | 2.7 |
| IDO1 | 0 | 0 | 0 | 0.1 | 0 | 0 | 0 | 0 |
| IFI44L | 3 | 4.4 | 2.6 | 9.3 | 5.1 | 2.3 | 9.6 | 6.9 |
| IFITM3 | 30.1 | 35.3 | 20.5 | 30.7 | 31.5 | 25.3 | 28.1 | 39.9 |
| IFNG | 17.5 | 0.2 | 25.6 | 5.5 | 3.5 | 0.5 | 2.7 | 0.7 |
| IGSF6 | 11.2 | 6.8 | 13.4 | 8.5 | 10.3 | 6.9 | 7.4 | 9.1 |
| IL10 | 0.2 | 0 | 4.2 | 0.5 | 0 | 0.2 | 0.1 | 0.2 |
| IL12B | 0 | 0 | 0 | 0 | 0 | 0 | 0 | 0 |
| IL12RB2 | 0.2 | 0 | 1.7 | 0.8 | 0.5 | 0.4 | 0.2 | 1.1 |
| IL15 | 3.2 | 0.7 | 0.9 | 5.1 | 5.4 | 0.6 | 2.7 | 6.3 |
| IL18R1 | 9.7 | 0.9 | 12.7 | 13.4 | 7.8 | 2.7 | 4.1 | 18.7 |
| IL18RAP | 11 | 0.3 | 3.2 | 6.7 | 2.4 | 0.3 | 0.8 | 22 |
| IL1A | 0.4 | 0.2 | 0 | 0.3 | 0 | 0.1 | 0.2 | 0.1 |
| IL1B | 67.2 | 33.7 | 13.8 | 40.4 | 46.6 | 28.3 | 29.1 | 41 |
| IL1RL1 | 0 | 0 | 0.7 | 0.4 | 0.8 | 0 | 0 | 0.6 |
| IL2 | 0.2 | 0.1 | 2.1 | 0.8 | 21.1 | 0 | 1.4 | 0.2 |
| IL26 | 0.1 | 0 | 4.9 | 0.3 | 0 | 0 | 0 | 0.3 |
| IL2RA | 0.7 | 1.2 | 7.5 | 7 | 10.1 | 38.7 | 12.6 | 1 |
| IL2RB | 12.9 | 1.6 | 18.3 | 10 | 6.8 | 11.2 | 5.8 | 20.8 |
| IL4 | 0 | 0 | 0 | 0 | 0 | 0 | 0 | 0 |
| IL4R | 7.6 | 13.2 | 1.4 | 8.2 | 10.4 | 6.6 | 11.6 | 5.6 |
| IL5RA | 1.2 | 0 | 0 | 0.4 | 0 | 0 | 0.3 | 0.1 |
| IL7 | 2.7 | 0.1 | 1.7 | 0.4 | 0 | 0.8 | 0.3 | 0.1 |
| IL7R | 964.6 | 833.5 | 132.6 | 1855 | 2012.8 | 356.9 | 1369.3 | 2101.5 |
| INPP4B | 8.3 | 21.8 | 10.9 | 28.8 | 27.2 | 19.5 | 36.6 | 9.8 |
| IRF4 | 0.6 | 1.3 | 6.5 | 0.8 | 1.6 | 1.4 | 0.7 | 0.2 |
| IRF8 | 2.8 | 1.8 | 0.6 | 1.9 | 1.6 | 1.5 | 1.8 | 2 |
| ITGA1 | 0.1 | 0.6 | 1.4 | 2.8 | 17.2 | 0 | 0.9 | 0.3 |
| ITGAE | 11 | 8 | 37.1 | 8.3 | 14.8 | 7.8 | 7.5 | 9.6 |
| ITGAL | 56.4 | 10.2 | 27.2 | 23.2 | 17.9 | 11.2 | 17.8 | 23.3 |
| ITGAV | 1.2 | 0.4 | 0.7 | 0.8 | 1.2 | 0.5 | 0.7 | 0.9 |
| ITGB1 | 324.2 | 14.1 | 173.9 | 87.8 | 17.5 | 87 | 172.7 | 40.4 |
| ITK | 19.8 | 23.3 | 10.7 | 16 | 23.8 | 18.9 | 20.2 | 8.2 |
| KCNA3 | 2.1 | 2.7 | 3.6 | 3.9 | 5.7 | 1.8 | 3.3 | 2.1 |
| KCNG2 | 0 | 0 | 0 | 0 | 0 | 0 | 0 | 0 |
| KIR2DL1 | 1.2 | 0.1 | 0 | 0 | 0 | 0.1 | 0 | 0 |
| KIR2DL4 | 0.1 | 0 | 0 | 0 | 0 | 0 | 0 | 0 |
| KIR3DL2 | 0.7 | 0.3 | 1.8 | 0.3 | 1.1 | 0.2 | 0.3 | 0.1 |
| KLF2 | 951.2 | 676.2 | 395.3 | 932.6 | 988.7 | 729.9 | 843.8 | 580.7 |
| KLF3 | 57.1 | 28.8 | 33.5 | 37.9 | 40.8 | 34 | 32.8 | 38.9 |
| KLRB1 | 632.4 | 43 | 215.4 | 1458.2 | 1470.5 | 56.3 | 633.5 | 4560.8 |
| KLRC3 | 0.1 | 0.1 | 0 | 0.1 | 0 | 0.2 | 0.1 | 0.2 |
| KLRC4 | 1.2 | 0.6 | 1.9 | 0.7 | 0 | 1.1 | 0.6 | 2 |
| KLRD1 | 49.3 | 2.3 | 11.1 | 3.1 | 4.6 | 2 | 2.5 | 35.7 |
| KLRF1 | 25.5 | 2.7 | 20.8 | 3.3 | 1.7 | 1.6 | 2.7 | 56.1 |
| KLRG1 | 114.4 | 4.4 | 18.3 | 67 | 16.5 | 5.7 | 24 | 197 |
| KLRK1 | 1.1 | 0.1 | 0 | 0.1 | 0 | 0.2 | 0.1 | 2.4 |
| KYNU | 2.8 | 0.8 | 0 | 1.1 | 1.3 | 0.4 | 1 | 0.9 |
| LAG3 | 13.2 | 1.3 | 17.3 | 9 | 10.6 | 1.2 | 4.2 | 28.5 |
| LAIR2 | 61.7 | 2 | 34.3 | 9.8 | 3 | 61.2 | 9.4 | 1.8 |
| LAMP3 | 0 | 0.2 | 0.5 | 0.4 | 0 | 0.3 | 0.4 | 0.3 |
| LAT | 100.1 | 83.6 | 186.7 | 78.3 | 69.1 | 72.9 | 89.8 | 72.1 |
| LCK | 131.3 | 99.7 | 211.6 | 105.3 | 103.9 | 102.6 | 103 | 109.5 |
| LEF1 | 6.7 | 104 | 65.2 | 19.4 | 16.6 | 94.4 | 51 | 3.3 |
| LHCGR | 0 | 0 | 0 | 0 | 0 | 0 | 0 | 0 |
| LILRA2 | 1.4 | 1.1 | 0 | 1.1 | 0 | 0.8 | 1.1 | 1.2 |
| LILRA4 | 0.3 | 0.3 | 0 | 0.3 | 1.2 | 0.2 | 0.3 | 0.1 |
| LILRB2 | 2.6 | 1.9 | 1.8 | 2 | 2.2 | 1.6 | 1.9 | 2.1 |
| LIME1 | 1.1 | 0.4 | 1.4 | 0.7 | 1.8 | 0.7 | 0.6 | 0.9 |
| LITAF | 169.4 | 59.5 | 57.6 | 75.7 | 34.6 | 62.7 | 61.4 | 135.7 |
| LST1 | 99.4 | 72 | 57.3 | 112.8 | 112.6 | 68.4 | 98.5 | 250.2 |
| LTA | 4.7 | 7.6 | 3.7 | 9.2 | 3 | 7 | 10.5 | 10.8 |
| LTB | 396.3 | 1805.5 | 403.2 | 2453.5 | 2463.6 | 1644.3 | 2780.8 | 2332.5 |
| LTC4S | 7.9 | 2.7 | 0 | 14.5 | 15.8 | 3.2 | 14.8 | 22.9 |
| LY86 | 6.3 | 6.2 | 2 | 4.8 | 7.2 | 4.3 | 4.1 | 5 |
| LY9 | 23.9 | 21.6 | 6.3 | 22.2 | 18.1 | 16 | 22.8 | 26.3 |
| MAK | 0.1 | 0.1 | 0.5 | 0.1 | 0 | 0.1 | 0.1 | 0.1 |
| MAN1A1 | 13 | 1.5 | 3.8 | 6.7 | 8.2 | 1.8 | 3.6 | 13.3 |
| MANEA | 2 | 1.1 | 4.9 | 1.6 | 1.9 | 1.5 | 1.6 | 1.8 |
| MAP3K13 | 6.8 | 6.7 | 5.8 | 6.8 | 4.8 | 4.8 | 6.6 | 9.1 |
| MAP4K1 | 15.6 | 5.1 | 36.6 | 10.8 | 13 | 6.5 | 10.1 | 12.2 |
| MAP4K2 | 8.8 | 12.1 | 18.7 | 13.2 | 10.7 | 13.2 | 14.2 | 7.7 |
| MAP9 | 2.3 | 1.6 | 1.2 | 2.4 | 4.3 | 1.7 | 2.3 | 3.3 |
| MARCO | 0.7 | 0.4 | 0 | 0.4 | 0 | 0.3 | 0.3 | 0.6 |
| MAST1 | 0 | 0 | 0 | 0 | 0 | 0 | 0 | 0.5 |
| MBTPS1 | 14.5 | 15.4 | 7.5 | 15.4 | 11.2 | 14.7 | 15.5 | 17.6 |
| MEFV | 0.4 | 0.3 | 0 | 0.3 | 0 | 0.3 | 0.3 | 0.4 |
| MGAM | 0.1 | 0 | 0 | 0 | 0 | 0 | 0 | 0 |
| MICAL3 | 0.5 | 0.3 | 1.5 | 1.2 | 0 | 0.7 | 1.1 | 1.1 |
| MKI67 | 0.2 | 0.1 | 150.5 | 0.1 | 0.3 | 0 | 0.3 | 0.1 |
| MMP25 | 0.2 | 0.2 | 0 | 0.7 | 0.6 | 0.1 | 0.4 | 0.5 |
| MMP9 | 0 | 0 | 0 | 0.1 | 0 | 0 | 0.1 | 0.1 |
| MNDA | 24.8 | 12.9 | 17.6 | 15.5 | 16.5 | 13.8 | 12.8 | 20.3 |
| MS4A1 | 2.2 | 1.2 | 0.5 | 3.2 | 1.2 | 1.3 | 1.7 | 2.9 |
| MS4A2 | 0 | 0 | 0 | 0 | 0 | 0 | 0 | 0 |
| MS4A3 | 0 | 0 | 0 | 0 | 0 | 0 | 0 | 0 |
| MS4A6A | 7.8 | 5.6 | 5.6 | 6.4 | 5.5 | 7.8 | 8.7 | 8.2 |
| MSC | 6.2 | 0.1 | 5.9 | 0.8 | 3.1 | 0.4 | 0.9 | 1.1 |
| MXD1 | 4.8 | 2.4 | 2.8 | 3 | 3.4 | 2.6 | 2.9 | 4.9 |
| MYB | 0 | 1.1 | 8 | 0.2 | 0.6 | 1.4 | 0.3 | 0.1 |
| NAALADL1 | 2.5 | 3.4 | 1.9 | 3.7 | 4.7 | 5.2 | 3.6 | 3 |
| NAB1 | 1.9 | 1.1 | 4.2 | 1.7 | 2.4 | 0.9 | 2 | 1.6 |
| NCF2 | 6.4 | 4.2 | 2.1 | 5.5 | 3.5 | 3.3 | 4.8 | 4.8 |
| NCR3 | 18.2 | 5.5 | 12.4 | 27.8 | 20.9 | 17.6 | 13 | 366 |
| NFE2 | 1.4 | 0.6 | 0 | 0.9 | 0 | 0.8 | 0.5 | 0.7 |
| NFKB2 | 6.1 | 6.5 | 2.2 | 10.5 | 4.8 | 7 | 10.6 | 11.3 |
| NIPSNAP3B | 0.1 | 0.2 | 0 | 0.3 | 2.4 | 0.1 | 0.1 | 0.1 |
| NKG7 | 5577 | 62.3 | 386.3 | 301.1 | 132.6 | 58.9 | 74.8 | 2250 |
| NLRP3 | 2.1 | 1.2 | 1.2 | 1.8 | 2.4 | 0.7 | 1.6 | 2 |
| NOD2 | 0.9 | 0.3 | 0 | 1 | 0 | 0.8 | 0.8 | 0.3 |
| NOTCH1 | 1.4 | 1.2 | 1.9 | 1.1 | 0.9 | 1.4 | 1 | 1 |
| NOTCH2 | 1.9 | 1.3 | 0.5 | 1.6 | 0.9 | 1.1 | 1.5 | 1.7 |
| NPAS1 | 0 | 0.1 | 0 | 0 | 0 | 0 | 0.1 | 0.1 |
| NPL | 3.7 | 1.9 | 0 | 2.3 | 2.7 | 2.2 | 2.1 | 2.7 |
| NR4A1 | 1.9 | 1.7 | 0.6 | 1.9 | 0.8 | 1.7 | 1.9 | 2.3 |
| NR4A2 | 7.4 | 1.5 | 2 | 19.3 | 6.6 | 3.4 | 11.6 | 42.3 |
| NR4A3 | 0.1 | 0 | 0 | 0.2 | 0 | 0 | 0.1 | 0.6 |
| NTRK1 | 0 | 0 | 0 | 0 | 0 | 0.1 | 0 | 0.1 |
| OSM | 4.7 | 1.8 | 0.9 | 11.3 | 7.9 | 2.2 | 13.5 | 26.2 |
| P2RX1 | 0.3 | 0.3 | 0.6 | 0.5 | 1.5 | 0.5 | 0.4 | 0.3 |
| P2RX5 | 3.6 | 2.8 | 14.1 | 7.3 | 5.8 | 3.6 | 4.1 | 6.8 |
| P2RY10 | 14.4 | 16.5 | 25 | 24.2 | 26 | 25.4 | 26.3 | 10.6 |
| P2RY13 | 0.7 | 0.4 | 0 | 0.5 | 0 | 0.4 | 0.4 | 0.5 |
| P2RY14 | 0.1 | 0.2 | 0 | 0.7 | 1.6 | 0.3 | 0.5 | 8.8 |
| P2RY2 | 0 | 0 | 0 | 0 | 0 | 0 | 0.1 | 0.1 |
| PADI4 | 1 | 3.7 | 0.8 | 0.4 | 0 | 3.1 | 0.7 | 0.4 |
| PASK | 1.4 | 9.7 | 3.8 | 6.3 | 3.3 | 7.2 | 19.9 | 1.8 |
| PBXIP1 | 50.8 | 37.4 | 41.5 | 64.9 | 59.6 | 52.4 | 60.4 | 66.9 |
| PDCD1 | 5.1 | 0.2 | 3.3 | 4.4 | 3 | 1 | 3.1 | 5 |
| PDCD1LG2 | 0.1 | 0 | 1.4 | 0.2 | 0 | 0.1 | 0.1 | 0 |
| PDE6C | 0 | 0 | 0 | 0 | 0 | 0 | 0 | 0 |
| PDK1 | 2.4 | 11.1 | 3.8 | 2.7 | 3.2 | 9.2 | 3.9 | 2.4 |
| PIK3IP1 | 46.7 | 133 | 16.7 | 63.4 | 68.5 | 120.8 | 78.7 | 44.2 |
| PKD2L2 | 1.4 | 0.7 | 3.1 | 0.9 | 2.9 | 0.9 | 0.6 | 0.6 |
| PLA1A | 0.1 | 0 | 0 | 0 | 0 | 0 | 0.1 | 0 |
| PLA2G7 | 0.3 | 0.3 | 0 | 0.3 | 0 | 0.4 | 0.2 | 0.4 |
| PLCH2 | 0.5 | 0.4 | 0.4 | 0.7 | 1.3 | 0.9 | 0.7 | 1 |
| PLEKHF1 | 26.8 | 4.7 | 12.9 | 8.8 | 3.6 | 3.7 | 6.7 | 20.4 |
| PLEKHG3 | 12.3 | 0.2 | 3.9 | 4.8 | 2.9 | 0.6 | 3.6 | 6.3 |
| PMCH | 0 | 0 | 0 | 0 | 0 | 0.1 | 0 | 0 |
| PNOC | 0.6 | 0.3 | 0 | 0.4 | 0 | 0.1 | 0.3 | 0.3 |
| PPBP | 20.5 | 9.6 | 2.4 | 12.4 | 12 | 12.5 | 9.5 | 10.6 |
| PPFIBP1 | 0.2 | 0.5 | 0 | 0.4 | 0.3 | 0.5 | 0.5 | 0.7 |
| PRDM1 | 39.5 | 2.5 | 17.4 | 37.4 | 45.1 | 13.7 | 25 | 52.2 |
| PRF1 | 185.7 | 3 | 29.9 | 19.6 | 10 | 6.1 | 5.5 | 137.5 |
| PRG2 | 0 | 0 | 0 | 0 | 0 | 0 | 0 | 0 |
| PTGDR | 22.1 | 0.2 | 2.3 | 6.8 | 2.2 | 0.3 | 1.2 | 16.3 |
| PTGER2 | 33 | 5 | 11.8 | 49.6 | 38.4 | 23.7 | 36.8 | 45.2 |
| PTGIR | 0.7 | 4.7 | 0.8 | 0.6 | 0 | 4.3 | 0.9 | 0.6 |
| PTPRCAP | 1.7 | 1.1 | 0 | 1.7 | 2.3 | 0.6 | 1.3 | 3.5 |
| PTPRG | 0 | 0 | 0 | 0 | 0 | 0 | 0 | 0 |
| PVRIG | 0.5 | 0.3 | 2.2 | 0.4 | 1.3 | 0.2 | 0.4 | 0.8 |
| QPCT | 0.5 | 0.4 | 0 | 0.7 | 0 | 0.3 | 0.5 | 0.4 |
| RAB27B | 7.6 | 2.2 | 3.9 | 3.5 | 0 | 0.6 | 2.3 | 1.7 |
| RALGPS2 | 0.5 | 4.3 | 0.3 | 2.1 | 5.1 | 1.8 | 3.1 | 2.9 |
| RASA3 | 24 | 21 | 13.6 | 19.4 | 27.7 | 21 | 21.8 | 16.8 |
| RASGRP2 | 47.7 | 53.6 | 70.1 | 44.2 | 60.5 | 50.3 | 50.3 | 33.6 |
| RASGRP3 | 0.5 | 0.3 | 0.7 | 0.4 | 0 | 0.3 | 0.5 | 0.3 |
| RASSF4 | 3.5 | 1.1 | 0 | 1.1 | 1.7 | 1.3 | 1.2 | 1.7 |
| RBPJ | 27.7 | 23.5 | 25.5 | 35.7 | 35.6 | 25.4 | 31.5 | 29.9 |
| RCAN3 | 12.6 | 166.6 | 62.8 | 123.8 | 124.4 | 167.7 | 146.4 | 73 |
| REL | 67.2 | 19.3 | 16.6 | 40.9 | 45.7 | 34.4 | 33.9 | 58.8 |
| RENBP | 2.7 | 3.8 | 2.3 | 1.8 | 1.4 | 2.9 | 2.6 | 2.1 |
| REPS2 | 0 | 0.1 | 0 | 0.1 | 0 | 0 | 0.1 | 0 |
| RGS1 | 9.6 | 5.3 | 61.3 | 21.1 | 25.6 | 40.4 | 15.5 | 8.7 |
| RGS13 | 0.2 | 0 | 0 | 0 | 0 | 0.1 | 0 | 0.1 |
| RGS16 | 0 | 0.2 | 0.7 | 0.3 | 3.5 | 0.2 | 1 | 0.3 |
| RGS2 | 33.6 | 10.5 | 1.3 | 44.7 | 42.4 | 16.8 | 25.9 | 113.3 |
| RNASE2 | 1.5 | 1 | 0 | 1.3 | 2.9 | 0.4 | 1.3 | 0.9 |
| RNASE6 | 4.9 | 3.3 | 4.9 | 4.7 | 2 | 1.9 | 5.6 | 3.3 |
| RRP12 | 0.7 | 0.8 | 1.9 | 1 | 0.5 | 0.8 | 0.8 | 0.9 |
| RRP9 | 5.2 | 5.1 | 12.3 | 5.3 | 8.2 | 4.8 | 5.3 | 6.4 |
| RSAD2 | 1.1 | 1.6 | 0.5 | 1.8 | 0.6 | 0.6 | 1.8 | 2 |
| RYR1 | 0.1 | 0 | 0.2 | 0.1 | 0.1 | 0.1 | 0.1 | 0.1 |
| S100A12 | 47.6 | 30.9 | 14.6 | 36.1 | 27 | 31.6 | 30 | 42.8 |
| S1PR1 | 20.9 | 14 | 16.4 | 15.5 | 13.9 | 9.8 | 17.9 | 10.2 |
| S1PR5 | 44.8 | 0.5 | 0.8 | 4.7 | 1 | 0.4 | 0.6 | 31 |
| SAMSN1 | 66.3 | 16.9 | 21.9 | 55.9 | 51.5 | 28.5 | 41.8 | 19.4 |
| SCN9A | 0 | 0.1 | 0 | 0 | 0 | 0 | 0 | 0 |
| SEC31B | 2.4 | 2 | 1.5 | 2.2 | 0.5 | 2.3 | 2.5 | 2.7 |
| SELL | 91.6 | 190.6 | 184.8 | 44.1 | 5.2 | 181 | 105.3 | 20.8 |
| SERGEF | 10 | 9.7 | 11.5 | 10.7 | 8.5 | 10.3 | 11.1 | 9.4 |
| SH2D1A | 31.7 | 16.6 | 120.6 | 28.4 | 13.5 | 13 | 20.7 | 49.2 |
| SIGLEC1 | 0.1 | 0.1 | 0.3 | 0.1 | 0 | 0.1 | 0.1 | 0.1 |
| SIK1 | 0.2 | 0.2 | 0.4 | 0.5 | 0 | 0.2 | 0.4 | 2.4 |
| SIRPG | 1.7 | 19 | 72.3 | 18.6 | 28.3 | 22 | 22.8 | 15.1 |
| SIT1 | 49 | 21.1 | 123.6 | 39.2 | 27.3 | 26.7 | 38 | 29.9 |
| SKAP1 | 161.9 | 94.6 | 162.8 | 122.1 | 103.3 | 121.4 | 114.2 | 118.9 |
| SKIL | 3.7 | 3.8 | 2 | 4.1 | 3 | 2 | 3 | 8.6 |
| SLAMF1 | 21.8 | 5.1 | 27.6 | 24 | 15.9 | 7.8 | 16.5 | 28.2 |
| SLAMF6 | 19.6 | 6.3 | 32.2 | 11.4 | 8.5 | 9.2 | 11.4 | 14.8 |
| SLAMF8 | 0.6 | 0 | 1.6 | 0.2 | 0.6 | 0 | 0 | 0.1 |
| SLC12A8 | 0 | 0 | 0 | 0 | 0 | 0 | 0 | 0 |
| SLC15A3 | 1 | 1.1 | 0.8 | 0.9 | 0 | 0.4 | 0.7 | 0.9 |
| SLC2A6 | 2 | 1.2 | 0.7 | 1.3 | 0.8 | 1 | 1.3 | 1.8 |
| SLCO5A1 | 0 | 0 | 0 | 0 | 0 | 0 | 0 | 0 |
| SMPD3 | 0.4 | 0.3 | 0.3 | 0.3 | 0.4 | 1.4 | 0.3 | 0.3 |
| SMPDL3B | 0 | 0 | 0 | 0.1 | 0 | 0 | 0 | 0.1 |
| SOCS1 | 16.2 | 15.2 | 2.8 | 27.4 | 33 | 11.6 | 29.5 | 32.8 |
| SP1 | 3 | 2.8 | 5 | 3.3 | 4.4 | 2.9 | 3.5 | 3.1 |
| SP140 | 24.1 | 9.5 | 42.5 | 25.3 | 29.3 | 11.9 | 20.7 | 21.7 |
| SPAG4 | 0.2 | 0.1 | 0 | 0.3 | 0 | 0.2 | 0.3 | 0.4 |
| SPIB | 0.9 | 0.6 | 0 | 0.5 | 0 | 0.4 | 0.6 | 0.9 |
| SPOCK2 | 50.9 | 34.2 | 61.2 | 52.9 | 53.7 | 40.1 | 51.8 | 92 |
| ST3GAL6 | 0.3 | 0.2 | 0 | 0.4 | 0 | 0 | 0.2 | 0 |
| ST6GALNAC4 | 2.6 | 3.9 | 5.1 | 4.6 | 8.8 | 5 | 4.2 | 7.1 |
| ST8SIA1 | 0.4 | 0.2 | 2 | 1 | 1.1 | 1.4 | 1.5 | 0.1 |
| STAP1 | 0.2 | 4 | 0 | 1.8 | 1.4 | 1 | 1.8 | 1.1 |
| STAT3 | 28.2 | 25.4 | 8.7 | 24.5 | 23.4 | 24.7 | 27.7 | 25.6 |
| STEAP4 | 0.2 | 0.2 | 0 | 0.3 | 0 | 0.2 | 0.2 | 0.2 |
| STXBP6 | 0.4 | 0 | 0 | 0.1 | 0 | 0 | 0 | 0 |
| TBX21 | 43 | 0.7 | 7.4 | 7.3 | 2.5 | 0.9 | 2.3 | 13.2 |
| TCF3 | 4.8 | 5 | 7.4 | 4 | 4.8 | 4.3 | 4.4 | 5.2 |
| TCF7 | 33.4 | 170.2 | 74.7 | 94.9 | 129.3 | 92.5 | 120.7 | 64.6 |
| TCL1A | 2.5 | 1.2 | 0 | 1.3 | 0 | 1.4 | 1.3 | 1.4 |
| TEC | 0.4 | 1 | 1 | 0.9 | 0.6 | 0.7 | 0.7 | 0.9 |
| TEP1 | 1.2 | 0.6 | 1.4 | 0.9 | 1 | 0.8 | 1 | 1 |
| TGFB1 | 133.7 | 32.5 | 60.2 | 70.9 | 62.2 | 37.6 | 58.3 | 105.9 |
| TIA1 | 10.8 | 8.7 | 10.4 | 9.3 | 8.7 | 7.9 | 9.7 | 10.3 |
| TLR1 | 2.2 | 2.1 | 1.8 | 1.7 | 2.2 | 1.2 | 2.6 | 1.8 |
| TLR2 | 0.9 | 0.9 | 1 | 1 | 1.2 | 0.7 | 0.9 | 0.8 |
| TLR7 | 0.1 | 0.1 | 0 | 0.1 | 0 | 0.1 | 0.1 | 0.1 |
| TLR8 | 0.2 | 0.2 | 0 | 0.3 | 1 | 0.2 | 0.2 | 0.2 |
| TMEM123 | 48.4 | 88.2 | 52.9 | 80.4 | 104.4 | 89 | 94.3 | 59.7 |
| TMEM156 | 13.3 | 9.1 | 16.5 | 15.6 | 39.4 | 9.9 | 21.7 | 12.9 |
| TNF | 8.3 | 2.1 | 3.1 | 7.6 | 6.3 | 3.1 | 6 | 20.9 |
| TNFAIP6 | 0.3 | 0.3 | 0 | 0.3 | 1.5 | 0.3 | 0.3 | 0.5 |
| TNFRSF10C | 0 | 0.1 | 0 | 0 | 0 | 0.1 | 0.1 | 0 |
| TNFRSF11A | 0 | 0 | 0 | 0.1 | 0 | 0.1 | 0.4 | 0.1 |
| TNFRSF13B | 0.3 | 0.3 | 0 | 0.3 | 0 | 0.3 | 0.3 | 0.5 |
| TNFRSF17 | 0.7 | 0.4 | 0 | 0.6 | 0 | 0.5 | 0.4 | 0.3 |
| TNFRSF4 | 6.8 | 2.9 | 24.1 | 46 | 37.5 | 24 | 71.4 | 7.6 |
| TNFSF14 | 13 | 0.7 | 0 | 10 | 2.9 | 2 | 4.8 | 27.5 |
| TNIP3 | 0.7 | 0.3 | 4.3 | 0.6 | 0.9 | 0.1 | 0.2 | 0.9 |
| TPSAB1 | 0 | 0 | 0 | 0 | 0 | 0 | 0 | 0 |
| TRAF4 | 2 | 0.9 | 5.3 | 2.4 | 1.5 | 1.7 | 2.2 | 3.8 |
| TRAT1 | 49.8 | 91.3 | 99.6 | 86.2 | 113.5 | 53.3 | 99.6 | 57.1 |
| TREM1 | 1 | 1.3 | 1.9 | 1.8 | 2.3 | 0.5 | 1.2 | 1.2 |
| TREM2 | 0 | 0 | 0 | 0 | 0 | 0 | 0 | 0 |
| TREML2 | 0.1 | 0.1 | 0 | 0.1 | 0 | 0 | 0.1 | 0 |
| TRIB2 | 6.6 | 8.3 | 31.7 | 6.7 | 7.2 | 9 | 10.9 | 5 |
| TRPM4 | 0 | 0 | 0 | 0 | 0.5 | 0.1 | 0.1 | 0 |
| TSHR | 0 | 0 | 1.6 | 0 | 0 | 0.2 | 0.1 | 0 |
| TTC38 | 11.7 | 0.7 | 1.3 | 1.4 | 4.9 | 1 | 1 | 2 |
| TXK | 1.6 | 37.9 | 12.4 | 15.8 | 16 | 32 | 21.6 | 26.2 |
| UBASH3A | 6.3 | 9.9 | 12.5 | 8.9 | 8.5 | 8.4 | 10.6 | 6.4 |
| UGT2B17 | 0 | 0 | 0 | 0 | 0 | 0 | 0 | 0 |
| UPK3A | 0.6 | 0.4 | 0 | 0.5 | 0 | 0.2 | 0.3 | 0.6 |
| USP33 | 17.4 | 12.4 | 11.9 | 13.6 | 15.5 | 10.6 | 12.9 | 15.4 |
| VCAM1 | 0 | 0 | 0.6 | 0 | 0 | 0 | 0 | 0 |
| VILL | 3.1 | 3.3 | 1.9 | 3.9 | 6.1 | 2.9 | 3.4 | 3.4 |
| VNN1 | 0.3 | 0.1 | 0 | 0 | 0 | 0 | 0.1 | 0 |
| VNN2 | 3 | 4.9 | 1.7 | 2.3 | 1.1 | 4.7 | 2.4 | 2.3 |
| VNN3 | 0.3 | 0.2 | 0.9 | 0.1 | 0 | 0.3 | 0.1 | 0.1 |
| VPREB3 | 2.3 | 1.6 | 0 | 2 | 0 | 1 | 1.7 | 1.3 |
| VPS37B | 13.9 | 5.2 | 3.1 | 11.9 | 7.7 | 5.8 | 8.5 | 19 |
| WNT5B | 0 | 0 | 0 | 0 | 0 | 0 | 0 | 0 |
| WNT7A | 1.6 | 4.4 | 0 | 7 | 2.5 | 1.6 | 9.4 | 0.4 |
| XCL1 | 6.6 | 0.3 | 0 | 7.3 | 1.7 | 0.9 | 0.8 | 2.3 |
| ZAP70 | 52.7 | 24.4 | 66 | 33.7 | 33.1 | 22.1 | 32.2 | 43.3 |
| ZBP1 | 24.3 | 5.1 | 7.9 | 14.5 | 12.5 | 7.7 | 12.1 | 9.8 |
| ZBTB10 | 1.9 | 2.6 | 0.6 | 3.7 | 3.5 | 2.6 | 4.2 | 2.9 |
| ZBTB32 | 0.1 | 0.2 | 15.8 | 0.1 | 0 | 0.4 | 0.4 | 0.1 |
| ZFP36L2 | 401 | 237.3 | 78.5 | 555.2 | 455.4 | 182.3 | 395 | 864.4 |
| ZNF135 | 0.1 | 0.6 | 0.6 | 0.3 | 1.4 | 0.4 | 0.2 | 0.1 |
| ZNF165 | 0.3 | 0.2 | 0 | 0.2 | 0 | 0.1 | 0.3 | 0.2 |
| ZNF222 | 1.8 | 1.3 | 4.2 | 1.5 | 2.6 | 1.5 | 1.5 | 3.2 |
| ZNF286A | 0.8 | 0.5 | 0.6 | 0.6 | 0.4 | 0.6 | 0.5 | 0.5 |
| ZNF324 | 3.3 | 3.5 | 4 | 3.3 | 2.1 | 3.4 | 3.3 | 3.5 |
| ZNF442 | 0.1 | 0.3 | 0.6 | 0.1 | 0 | 0.1 | 0.2 | 0.1 |
| ZNF683 | 61.5 | 0.8 | 21.9 | 1.8 | 0 | 0.5 | 1.1 | 1 |

***** Expression values were adopted from integrative PBMC scRNA-Seq gene expression profiles for each cell subtype (1)

**Reference**

1. Stuart T, Butler A, Hoffman P, Hafemeister C, Papalexi E, Mauck WM, 3rd, et al. Comprehensive Integration of Single-Cell Data. Cell. 2019;177(7):1888-902 e21.
