# Supplementary material for: MAdCAM-1 costimulation in the presence of retinoic acid and TGF-β promotes HIV infection and differentiation of CD4+ T cells into CCR5+ TRM-like cells
Source: PLoS Pathog. 2023 Mar 10;19(3):e1011209. doi: 10.1371/journal.ppat.1011209 (PMC10032498; doi:10.1371/journal.ppat.1011209)
Supplement: S2 Table — (DOCX) [file ppat.1011209.s010.docx]

**S2 Table. GSEA analysis results of CD3 Ab + MAdCAM-1 + RA and CD3 Ab + CD28 Ab + RA treatment groups**

|  | MAdCAM-1 + RA | | | CD28 Ab + RA | | |
| --- | --- | --- | --- | --- | --- | --- |
| Biological Term | NES | NOM p-val | FDR q-val | NES | NOM p-val | FDR q-val |
| TRM_AMSEN | 1.64 | 0.000 | 0.022 | 1.34 | 0.051 | 0.088 |
| GSE14308_TH17_VS_NAIVE_CD4_TCELL_UP | 1.83 | 0.000 | 0.007 | 1.10 | 0.071 | 0.256 |
| BIOCARTA_VIP_PATHWAY | 1.90 | 0.000 | 0.004 | 1.25 | 0.178 | 0.126 |
| PD1_TCELL | 1.90 | 0.000 | 0.004 | 1.17 | 0.011 | 0.180 |
| KEGG_TGFB_SIGNALING | 1.84 | 0.001 | 0.007 | 1.35 | 0.065 | 0.085 |
| BIOCARTA_CSK_PATHWAY | 1.53 | 0.017 | 0.036 | 0.86 | 0.643 | 0.934 |
| TEM_AMSEN | 1.24 | 0.204 | 0.120 | 2.54 | 0.000 | 0.000 |
| TCM_AMSEN | 1.39 | 0.167 | 0.067 | 2.35 | 0.000 | 0.000 |
| BIOCARTA_TH1TH2_PATHWAY | 0.00 | 1.000 | 1.000 | 2.10 | 0.000 | 0.000 |
| BIOCARTA_IFNG_PATHWAY | 1.01 | 0.418 | 0.418 | 2.07 | 0.000 | 0.000 |
| TH_DIFFERENTIATION | 0.85 | 0.646 | 0.892 | 1.78 | 0.011 | 0.011 |
| TCELL_EXHAUSTION | 1.04 | 0.382 | 0.359 | 1.76 | 0.012 | 0.012 |
| BIOCARTA_IL22BP_PATHWAY | 1.07 | 0.370 | 0.302 | 1.64 | 0.011 | 0.019 |
| BIOCARTA_EGF_PATHWAY | 1.25 | 0.088 | 0.114 | 1.35 | 0.017 | 0.085 |
| BIOCARTA_TCR_PATHWAY | 1.19 | 0.093 | 0.151 | 1.28 | 0.002 | 0.112 |
| BIOCARTA_CYTOKINE_PATHWAY | 1.82 | 0.030 | 0.007 | 2.09 | 0.000 | 0.000 |
| BIOCARTA_CTLA4_PATHWAY | 1.64 | 0.030 | 0.022 | 1.84 | 0.000 | 0.010 |
| TRM_FITZPATRICK | 1.82 | 0.000 | 0.010 | 1.31 | 0.040 | 0.095 |

***** Nominal p-value or FDR values were in green background if their values are less than 0.05, otherwise, in orange background.

FDR: false discovery rate, NOM p-val: nominal p-val, NES: normalized enrichment score
