# Supplementary material for: MAdCAM-1 costimulation in the presence of retinoic acid and TGF-β promotes HIV infection and differentiation of CD4+ T cells into CCR5+ TRM-like cells
Source: PLoS Pathog. 2023 Mar 10;19(3):e1011209. doi: 10.1371/journal.ppat.1011209 (PMC10032498; doi:10.1371/journal.ppat.1011209)
Supplement: S3 Table — (DOCX) [file ppat.1011209.s011.docx]

**S3 Table. T_RM_ knowledge database**

| **Gene** | **Confidence*** | **Regulation** | **Papers** | **PubMed ID** |
| --- | --- | --- | --- | --- |
| S1PR1 | high | downregulated (-) | Behr et al., 2018 [1], Mami-Chouaib et al. [2], 2018, MacKay et al., 2013 [3], Milner & Goldrath, 2018 [4] ​, Steinbach et al., 2018 [5], Kumar et al., 2017 [6], Kiniry et al., 2018 [7], Kurd et al., 2020 [8] | 30131803, 30180905, 24162776, 29621697, 30555489, 28930685, 29139476, 32414833 |
| CCR7 | high | downregulated (-) | Behr et al., 2018 [1], Mami-Chouaib et al., 2018 [2], MacKay et al., 2015 [9], Milner & Goldrath, 2018​ [4], Yang et al., 2020 [10], Steinbach et al., 2018 [5], Kurd et al., 2020 [8] | 30131803, 30180905, 26682984, 29621697, 32229539, 30555489, 32414833 |
| KLF2 | high | downregulated (-) | Behr et al., 2018 [1], Milner & Goldrath, 2018​ [4], Kumar et al., 2017 [6], Kurd et al., 2020 [8], Schenkel & Masopust, 2014 [11] | 30131803, 29621697, 28930685, 32414833, 25526304 |
| SELL (CD62L) | high | downregulated (-) | MacKay et al., 2015 [9], Steinbach et al., 2018 [5], Kumar et al., 2017 [6], Kurd et al., 2020 [8] | 26682984, 30555489, 28930685, 32414833 |
| TBX21 (T-bet) | high | downregulated (-) | Behr et al., 2018 [1], Mami-Chouaib et al., 2018 [2], MacKay et al., 2015 [9], Milner & Goldrath, 2018 [4] ​, Kiniry et al., 2018 [7] | 30131803, 30180905, 26682984, 29621697, 29139476 |
| MKI67 | high | downregulated (-) | Mami-Chouaib et al., 2018 [2], Kumar et al., 2017 [6], Szabo et al., 2019 [12] | 30180905, 28930685, 30952804 |
| EOMES | high | downregulated (-) | MacKay et al., 2013, Schenkel & Masopust, 2014 [11], Parga-Vidal et al., 2021, Behr et al., 2018 [1], Hombrink et al., 2016 [13] | 24162776, 25526304, 34417257, 30131803, 27776108 |
| KLF3 | high | downregulated (-) | Kumar et al., 2017 [6], Anthony et al., 2021 [14], Chen et al., 2021 [15], Kok et al., 2020 [16] | 28930685, 34143731, 34440912, 32728699 |
| CX3CR1 | high | downregulated (-) | Steinbach et al., 2018 [5], Kumar et al., 2017 [6], Gerlach et al., 2016 [17], Batista et al., 2020 [18] | 30555489, 28930685, 27939671, 33172841 |
| NOTCH1 | high | upregulated (+) | Behr et al., 2018 [1], Mami-Chouaib et al., 2018 [2], Milner & Goldrath, 2018​ [4], | 30131803, 30180905, 29621697 |
| TGFB1 | high | upregulated (+) | Behr et al., 2018 [1], Mami-Chouaib et al., 2018 [2], MacKay et al., 2015 [9] | 30131803, 30180905, 26682984 |
| CD38 | high | upregulated (+) | Yang et al., 2020 [10], Kumar et al., 2017 [6], Szabo et al., 2019 [12], Yuan et al., 2021 [19] | 32229539, 28930685, 30952804, 34707601 |
| CD69 | high | upregulated (+) | Behr et al., 2018 [1], Mami-Chouaib et al., 2018 [2], MacKay et al., 2015 [9], Milner & Goldrath, 2018 [4] ​, Yang et al., 2020 [10], Steinbach et al., 2018 [5], Kumar et al., 2017 [6], Kiniry et al., 2018 [7], Kurd et al., 2020 [8], Walsh et al., 2019 | 30131803, 30180905, 26682984, 29621697, 32229539, 30555489, 28930685, 29139476, 32414833, 31243092 |
| ITGAE (CD103) | high | upregulated (+) | Behr et al., 2018 [1], Mami-Chouaib et al., 2018 [2], MacKay et al., 2015 [9], Milner & Goldrath, 2018​ [4], Yang et al., 2020 [10], Steinbach et al., 2018 [5], Kumar et al., 2017 [6], Kiniry et al., 2018 [7], Kurd et al., 2020 [8], Walsh et al., 2019 [20] | 30131803, 30180905, 26682984, 29621697, 32229539, 30555489, 28930685, 29139476, 32414833, 31243092 |
| AHR | high | upregulated (+) | Behr et al., 2018 [1], Mami-Chouaib et al., 2018 [2], Milner & Goldrath, 2018​ [4], Wu et al., 2018 [21] | 30131803, 30180905, 29621697, 30283442 |
| GZMB | high | upregulated (+) | Behr et al., 2018, [1] Mami-Chouaib et al., 2018 [2], Milner & Goldrath, 2018​ [4], Steinbach et al. 2018, [5], Schenkel & Masopust, 2014 [11] | 30131803, 30180905, 29621697, 30555489, 25526304 |
| IL10 | high | upregulated (+) | Kumar et al., 2017 [6], Yuan et al., 2021[19], Thompson et al., 2019[22], Topham et al., 2019[23] | 28930685, 34707601, 31365858, 31635290 |
| IL7 | high | upregulated (+) | Behr et al., 2018 [1], Milner & Goldrath, 2018​[4], Yeon et al., 2017[24] | 30131803, 29621697, 28894184 |
| IRF4 | high | upregulated (+) | Milner & Goldrath, 2018​, [4] Harberts et al., 2021[25], Barros et al., 2021[26], Hombrink et al., 2016 [13] | 29621697, 33859042, 34608235, 27776108 |
| NR4A1 (Nurr77) | high | upregulated (+) | Behr et al., 2018 [1], MacKay et al., 2013[3], Milner & Goldrath, 2018​ [4] | 30131803, 24162776, 29621697 |
| PRDM1 (Blimp-1) | high | upregulated (+) | Behr et al., 2018 [1], Milner & Goldrath, 2018​, [4] Chen et al., 2021[15] | 30131803, 29621697, 34440912 |
| Runx3 | high | upregulated (+) | Behr et al., 2018 [1], Mami-Chouaib et al., 2018 [2], Milner & Goldrath, 2018 [4] ​, Chen et al., 2021[15] | 30131803, 30180905, 29621697, 34440912 |
| ZNF683 (Hobit) | high | upregulated (+) | Behr et al., 2018 [1], Mami-Chouaib et al., 2018[2], Milner & Goldrath, 2018 [4] ​, Chen et al., 2021[15] | 30131803, 30180905, 29621697, 34440912 |
| CTLA4 | high | upregulated (+) | MacKay et al., 2013[3], Milner & Goldrath, 2018​ [4], Szabo et al., 2019[12] | 24162776, 29621697, 30952804 |
| CXCR3 | high | upregulated (+) | Mami-Chouaib et al., 2018[2], MacKay et al., 2015 [9], Muthuswamy et al., 2021[27] | 30180905, 26682984, 34607898 |
| CXCR6 | high | upregulated (+) | MacKay et al., 2015[9], Yang et al., 2020[10], Kumar et al., 2017 [6], Wein et al., 2019[28], Karaki et al., 2021[29] | 26682984, 32229539, 28930685, 31558615, 33692218 |
| HAVCR2 (TIM-3) | high | upregulated (+) | Mami-Chouaib et al., 2018[2], Yang et al., 2020[10], McCully et al., 2018[30] | 30180905, 32229539, 29427415 |
| IFNG | high | upregulated (+) | Behr et al., 2018[1], Mami-Chouaib et al., 2018[2], Milner & Goldrath, 2018​[4], Yang et al., 2020[10], Kumar et al., 2017[6], Corgnac et al., 2018[31] | 30131803, 30180905, 29621697, 32229539, 28930685, 30158938 |
| IL17A | high | upregulated (+) | Steinbach et al., 2018[5], Kumar et al., 2017[6], Kurihara et al., 2019[32] | 30555489, 28930685, 31300254 |
| ITGA1 | high | upregulated (+) | Behr et al., 2018[1], Mami-Chouaib et al., 2018[2], Yang et al., 2020[10], Steinbach et al., 2018[5], Kumar et al., 2017[6], MacKay et al., 2013[3] | 30131803, 30180905, 32229539, 30555489, 28930685, 24162776 |
| KLRG1 | high | upregulated (+) | MacKay et al., 2015[9], Milner & Goldrath, 2018[4]​, Steinbach et al., 2018[5] | 26682984, 29621697, 30555489 |
| PDCD1 | high | upregulated (+) | Behr et al., 2018, Mami-Chouaib et al., 2018, Milner & Goldrath, 2018​, Yang et al., 2020, Kumar et al., 2017 | 30131803, 30180905, 29621697, 32229539, 28930685 |
| TNF | high | upregulated (+) | Behr et al., 2018[1], Mami-Chouaib et al., 2018[2], Yang et al., 2020[10] | 30131803, 30180905, 32229539 |
| CD44 | high | upregulated (+) | Steinbach et al, 2018[5], Topham & Reilly, 2018[23], Menares et al., 2019[33] | 30555489, 29632527, 31562311 |
| IL7R | high | upregulated (+) | Steinbach et al., 2018[5], Schenkel & Masopust, 2014[11], Fitzpatrick et al., 2021[34], Clarke et al., 2019[35] | 30555489, 25526304, 33472060, 31227543 |
| IL2 | high | upregulated (+) | Steinbach et al., 2018[5], Kumar et al., 2017[6], Hondowicz et al., 2018[36], Pallett et al., 2017[37] | 30555489, 28930685, 28948612, 28526759 |
| CCR5 | high | upregulated (+) | Mami-Chouaib et al., 2018[2], Yang et al., 2020[10], Woodward Davis et al., 2019[38], Takamura, 2018[39] | 30180905, 32229539, 31801887, 29904388 |
| CD101 | high | upregulated (+) | Steinbach et al., 2018[5], Kumar et al., 2017[6], Ogongo et al., 2019[40], Yang et al., 2020[10] | 30555489, 28930685, 31130965, 32229539 |
| IL2RB (CD122) | high | upregulated (+) | MacKay et al., 2015[9], Milner & Goldrath, 2018​[4], Schenkel & Masopust, 2014[11], Weisberg et al., 2019[41] | 26682984, 29621697, 25526304, 31851923 |
| GZMA | high | upregulated (+) | Corgnac et al., 2018[31], Hashimoto et al., 2019[42], Koda et al., 2021[43] | 30158938, 31719197, 34294714 |
| IL2RA (CD25) | high | upregulated (+) | Behr et al., 2018[1], Wakim et al., 2012[44], Fitzpatrick et al., 2021[34], Szabo et al., 2019[12] | 30131803, 22922816, 33472060, 30952804 |
| IL15 | high | upregulated (+) | Mami-Chouaib et al., 2018[2], MacKay et al., 2015[9], Milner & Goldrath, 2018​[4] | 30180905, 26682984, 29621697 |
| CXCL16 | high | upregulated (+) | Wein et al., 2019[28], Muthuswamy et al., 2021[27], Vella et al., 2021[45], Zheng & Wakim, 2021[46] | 31558615, 34607898, 34362825, 34671115 |
| ICOS | high | upregulated (+) | MacKay et al., 2013[3], Peng et al., 2021[47], Wu et al., 2018[48], Mami-Chouaib et al., 2018[2] | 24162776, 34552595, 30283442, 30180905 |
| CCR4 | high | upregulated (+) | Shin & Iwasaki, 2013[49], Klicznik et al., 2019[50], Casciano et al., 2020 [51], McCully et al., 2018 [30] | 23947354, 31278120, 32318062, 29427415 |
| ELOVL7 | low | downregulated (-) | MacKay et al., 2013[3] | 24162776 |
| TLR1 | low | downregulated (-) | MacKay et al., 2013[3] | 24162776 |
| NOTCH2 | low | upregulated (+) | Hombrink et al., 2016 [13] | 27776108 |
| ADGRE5 | low | upregulated (+) | Mami-Chouaib et al., 2018[2] | 30180905 |
| BCL2 | low | upregulated (+) | Steinbach et al., 2018 [5] | 30555489 |
| CD244 | low | upregulated (+) | MacKay et al., 2013[3] | 24162776 |
| CDH1 | low | upregulated (+) | MacKay et al., 2013[3] | 24162776 |
| CHN2 | low | upregulated (+) | MacKay et al., 2013[3] | 24162776 |
| CRTAM | low | upregulated (+) | Kumar et al., 2017 [6] | 28930685 |
| HPGDS | low | upregulated (+) | MacKay et al., 2013[3] | 24162776 |
| INPP4B | low | upregulated (+) | MacKay et al., 2013[3] | 24162776 |
| ITGAL | low | upregulated (+) | Behr et al., 2018 [1] | 30131803 |
| ITGAV | low | upregulated (+) | Mami-Chouaib et al., 2018[2] | 30180905 |
| LITAF | low | upregulated (+) | MacKay et al., 2013[3] | 24162776 |
| NR4A2 | low | upregulated (+) | MacKay et al., 2013[3] | 24162776 |
| QPCT | low | upregulated (+) | MacKay et al., 2013[3] | 24162776 |
| SIK1 | low | upregulated (+) | MacKay et al., 2013[3] | 24162776 |
| SKIL | low | upregulated (+) | MacKay et al., 2013[3] | 24162776 |
| TMEM123 | low | upregulated (+) | MacKay et al., 2013[3] | 24162776 |
| VCAM1 | low | upregulated (+) | Mami-Chouaib et al., 2018[2] | 30180905 |
| XCL1 | low | upregulated (+) | MacKay et al., 2013[3] | 24162776 |
| BATF | low | upregulated (+) | Mami-Chouaib et al., 2018[2] | 30180905 |
| BCL6 | low | upregulated (+) | Milner & Goldrath, 2018​[2] | 29621697 |
| CXCL10 | low | upregulated (+) | Behr et al., 2018 [1] | 30131803 |
| CXCL9 | low | upregulated (+) | Behr et al., 2018 [1] | 30131803 |
| EGR2 | low | upregulated (+) | Milner & Goldrath, 2018​[4] | 29621697 |
| HIF1A | low | upregulated (+) | Mami-Chouaib et al., 2018[2] | 30180905 |
| RGS1 | low | upregulated (+) | Mami-Chouaib et al., 2018[2] | 30180905 |
| RGS2 | low | upregulated (+) | Mami-Chouaib et al., 2018[2] | 30180905 |
| STAT3 | low | upregulated (+) | Milner & Goldrath, 2018​ [4] | 29621697 |
| TIA1 | low | upregulated (+) | Mami-Chouaib et al., 2018[2] | 30180905 |
| TGFBR3L | low | upregulated (+) | Kumar et al., 2017 [6] | 28930685 |
| NFKB2 | low | upregulated (+) | Hombrink et al., 2016[13] | 27776108 |
| Foxo3 | low | upregulated (+) | Milner & Goldrath, 2018​[4] | 30131803 |
| IFITM3 | low | upregulated (+) | Behr et al., 2018 [1] | 30131803 |
| NAB1 | low | upregulated (+) | Mami-Chouaib et al., 2018[2] | 30180905 |
| RBPJ | low | upregulated (+) | Mami-Chouaib et al., 2018[2] | 30180905 |
| CCL2 | low | upregulated (+) | Behr et al., 2018 [1] | 30131803 |
| CCR8 | low | upregulated (+) | McCully et al., 2018 [30] | 29427415 |
| VPS37B | low | upregulated (+) | MacKay et al., 2013 [3] | 24162776, 28930685 |
| CMAHP | medium | downregulated (-) | MacKay et al., 2013 [3], Kumar et al., 2017[6] | 24162776, 28930685 |
| RASGRP2 | medium | downregulated (-) | MacKay et al., 2013 [3], Kumar et al., 2017[6] | 24162776, 28930685 |
| RIPOR2 | medium | downregulated (-) | MacKay et al., 2013 [3], Kumar et al., 2017[6] | 24162776, 28930685 |
| SLAMF6 | medium | downregulated (-) | MacKay et al., 2013 [3], Kumar et al., 2017[6] | 24162776, 28930685 |
| USP33 | medium | downregulated (-) | MacKay et al., 2013 [3], Kumar et al., 2017[6] | 32268106, 30131803 |
| TCF7 | medium | downregulated (-) | Wu et al., 2020[21], Behr et al., 2018 [1] | 24162776 |
| CXCL13 | medium | upregulated (+) | Clarke et al., 2019 [35], Kumar et al., 2017 [6] | 31227543, 28930685 |
| CCR6 | medium | upregulated (+) | Mami-Chouaib et al., 2018[2], Cheuk et al., 2017[52] | 30180905, 28214226 |
| HLA-DRA | medium | upregulated (+) | Mami-Chouaib et al., 2018[2], Yang et al., 2020 [10] | 30180905, 32229539 |
| ITGB1 | medium | upregulated (+) | Nizard, 2017 [53], Karaki et al., 2021 [29] | 28537262, 33692218 |
| CCL3 | medium | upregulated (+) | Behr et al., 2018 [1], Mami-Chouaib et al., 2018[2] | 30131803, 30180905 |
| CCL4 | medium | upregulated (+) | Behr et al., 2018 [1], Mami-Chouaib et al., 2018[2] | 30131803, 30180905 |
| CCL5 | medium | upregulated (+) | Behr et al., 2018 [1], Mami-Chouaib et al., 2018[2] | 30131803, 30180905 |
| DUSP6 | medium | upregulated (+) | Kumar et al., 2017 [6], Fitzpatrick et al., 2021 [34] | 28930685, 33472060 |

*Importance of genes is designated as high if cited as associated with T_RM_ cells in 3 or more publications, medium, in 2 publications, and low, in 1 publication.

**References**

1. Behr, F.M., et al., *Armed and Ready: Transcriptional Regulation of Tissue-Resident Memory CD8 T Cells.* Front Immunol, 2018. **9**: p. 1770.

2. Mami-Chouaib, F., et al., *Resident memory T cells, critical components in tumor immunology.* J Immunother Cancer, 2018. **6**(1): p. 87.

3. Mackay, L.K., et al., *The developmental pathway for CD103(+)CD8+ tissue-resident memory T cells of skin.* Nat Immunol, 2013. **14**(12): p. 1294-301.

4. Milner, J.J. and A.W. Goldrath, *Transcriptional programming of tissue-resident memory CD8(+) T cells.* Curr Opin Immunol, 2018. **51**: p. 162-169.

5. Steinbach, K., I. Vincenti, and D. Merkler, *Resident-Memory T Cells in Tissue-Restricted Immune Responses: For Better or Worse?* Front Immunol, 2018. **9**: p. 2827.

6. Kumar, B.V., et al., *Human Tissue-Resident Memory T Cells Are Defined by Core Transcriptional and Functional Signatures in Lymphoid and Mucosal Sites.* Cell Rep, 2017. **20**(12): p. 2921-2934.

7. Kiniry, B.E., et al., *Detection of HIV-1-specific gastrointestinal tissue resident CD8(+) T-cells in chronic infection.* Mucosal Immunol, 2018. **11**(3): p. 909-920.

8. Kurd, N.S., et al., *Early precursors and molecular determinants of tissue-resident memory CD8(+) T lymphocytes revealed by single-cell RNA sequencing.* Sci Immunol, 2020. **5**(47).

9. Mackay, L.K., et al., *T-box Transcription Factors Combine with the Cytokines TGF-beta and IL-15 to Control Tissue-Resident Memory T Cell Fate.* Immunity, 2015. **43**(6): p. 1101-11.

10. Yang, Q., et al., *Cutting Edge: Characterization of Human Tissue-Resident Memory T Cells at Different Infection Sites in Patients with Tuberculosis.* J Immunol, 2020. **204**(9): p. 2331-2336.

11. Schenkel, J.M. and D. Masopust, *Tissue-resident memory T cells.* Immunity, 2014. **41**(6): p. 886-97.

12. Szabo, P.A., M. Miron, and D.L. Farber, *Location, location, location: Tissue resident memory T cells in mice and humans.* Sci Immunol, 2019. **4**(34).

13. Hombrink, P., et al., *Programs for the persistence, vigilance and control of human CD8(+) lung-resident memory T cells.* Nat Immunol, 2016. **17**(12): p. 1467-1478.

14. Anthony, S.M., et al., *Protective function and durability of mouse lymph node-resident memory CD8(+) T cells.* Elife, 2021. **10**.

15. Chen, Y., et al., *Single-Cell Transcriptomics Reveals Core Regulatory Programs That Determine the Heterogeneity of Circulating and Tissue-Resident Memory CD8(+) T Cells.* Cells, 2021. **10**(8).

16. Kok, L., et al., *A committed tissue-resident memory T cell precursor within the circulating CD8+ effector T cell pool.* J Exp Med, 2020. **217**(10).

17. Gerlach, C., et al., *The Chemokine Receptor CX3CR1 Defines Three Antigen-Experienced CD8 T Cell Subsets with Distinct Roles in Immune Surveillance and Homeostasis.* Immunity, 2016. **45**(6): p. 1270-1284.

18. Batista, N.V., et al., *T Cell-Intrinsic CX3CR1 Marks the Most Differentiated Effector CD4(+) T Cells, but Is Largely Dispensable for CD4(+) T Cell Responses during Chronic Viral Infection.* Immunohorizons, 2020. **4**(11): p. 701-712.

19. Yuan, R., et al., *The Roles of Tissue-Resident Memory T Cells in Lung Diseases.* Front Immunol, 2021. **12**: p. 710375.

20. Walsh, D.A., et al., *The Functional Requirement for CD69 in Establishment of Resident Memory CD8(+) T Cells Varies with Tissue Location.* J Immunol, 2019. **203**(4): p. 946-955.

21. Wu, J., et al., *T Cell Factor 1 Suppresses CD103+ Lung Tissue-Resident Memory T Cell Development.* Cell Rep, 2020. **31**(1): p. 107484.

22. Thompson, E.A., et al., *Monocytes Acquire the Ability to Prime Tissue-Resident T Cells via IL-10-Mediated TGF-beta Release.* Cell Rep, 2019. **28**(5): p. 1127-1135 e4.

23. Topham, D.J. and E.C. Reilly, *Tissue-Resident Memory CD8(+) T Cells: From Phenotype to Function.* Front Immunol, 2018. **9**: p. 515.

24. Yeon, S.M., et al., *IL-7 plays a critical role for the homeostasis of allergen-specific memory CD4 T cells in the lung and airways.* Sci Rep, 2017. **7**(1): p. 11155.

25. Harberts, A., et al., *Interferon regulatory factor 4 controls effector functions of CD8(+) memory T cells.* Proc Natl Acad Sci U S A, 2021. **118**(16).

26. Barros, L., C. Ferreira, and M. Veldhoen, *The fellowship of regulatory and tissue-resident memory cells.* Mucosal Immunol, 2022. **15**(1): p. 64-73.

27. Muthuswamy, R., et al., *CXCR6 by increasing retention of memory CD8(+) T cells in the ovarian tumor microenvironment promotes immunosurveillance and control of ovarian cancer.* J Immunother Cancer, 2021. **9**(10).

28. Wein, A.N., et al., *CXCR6 regulates localization of tissue-resident memory CD8 T cells to the airways.* J Exp Med, 2019. **216**(12): p. 2748-2762.

29. Karaki, S., et al., *CXCR6 deficiency impairs cancer vaccine efficacy and CD8(+) resident memory T-cell recruitment in head and neck and lung tumors.* J Immunother Cancer, 2021. **9**(3).

30. McCully, M.L., et al., *CCR8 Expression Defines Tissue-Resident Memory T Cells in Human Skin.* J Immunol, 2018. **200**(5): p. 1639-1650.

31. Corgnac, S., et al., *The Emerging Role of CD8(+) Tissue Resident Memory T (T(RM)) Cells in Antitumor Immunity: A Unique Functional Contribution of the CD103 Integrin.* Front Immunol, 2018. **9**: p. 1904.

32. Kurihara, K., et al., *Significance of IL-17A-producing CD8(+)CD103(+) skin resident memory T cells in psoriasis lesion and their possible relationship to clinical course.* J Dermatol Sci, 2019. **95**(1): p. 21-27.

33. Menares, E., et al., *Tissue-resident memory CD8(+) T cells amplify anti-tumor immunity by triggering antigen spreading through dendritic cells.* Nat Commun, 2019. **10**(1): p. 4401.

34. FitzPatrick, M.E.B., et al., *Human intestinal tissue-resident memory T cells comprise transcriptionally and functionally distinct subsets.* Cell Rep, 2021. **34**(3): p. 108661.

35. Clarke, J., et al., *Single-cell transcriptomic analysis of tissue-resident memory T cells in human lung cancer.* J Exp Med, 2019. **216**(9): p. 2128-2149.

36. Hondowicz, B.D., et al., *IL-2 is required for the generation of viral-specific CD4(+) Th1 tissue-resident memory cells and B cells are essential for maintenance in the lung.* Eur J Immunol, 2018. **48**(1): p. 80-86.

37. Pallett, L.J., et al., *IL-2(high) tissue-resident T cells in the human liver: Sentinels for hepatotropic infection.* J Exp Med, 2017. **214**(6): p. 1567-1580.

38. Woodward Davis, A.S., et al., *The human tissue-resident CCR5(+) T cell compartment maintains protective and functional properties during inflammation.* Sci Transl Med, 2019. **11**(521).

39. Takamura, S., *Niches for the Long-Term Maintenance of Tissue-Resident Memory T Cells.* Front Immunol, 2018. **9**: p. 1214.

40. Ogongo, P., J.Z. Porterfield, and A. Leslie, *Lung Tissue Resident Memory T-Cells in the Immune Response to Mycobacterium tuberculosis.* Front Immunol, 2019. **10**: p. 992.

41. Weisberg, S.P., et al., *Tissue-Resident Memory T Cells Mediate Immune Homeostasis in the Human Pancreas through the PD-1/PD-L1 Pathway.* Cell Rep, 2019. **29**(12): p. 3916-3932 e5.

42. Hashimoto, K., et al., *Single-cell transcriptomics reveals expansion of cytotoxic CD4 T cells in supercentenarians.* Proc Natl Acad Sci U S A, 2019. **116**(48): p. 24242-24251.

43. Koda, Y., et al., *CD8(+) tissue-resident memory T cells promote liver fibrosis resolution by inducing apoptosis of hepatic stellate cells.* Nat Commun, 2021. **12**(1): p. 4474.

44. Wakim, L.M., et al., *The molecular signature of tissue resident memory CD8 T cells isolated from the brain.* J Immunol, 2012. **189**(7): p. 3462-71.

45. Vella, J.L., et al., *Dendritic cells maintain anti-tumor immunity by positioning CD8 skin-resident memory T cells.* Life Sci Alliance, 2021. **4**(10).

46. Zheng, M.Z.M. and L.M. Wakim, *Tissue resident memory T cells in the respiratory tract.* Mucosal Immunol, 2022. **15**(3): p. 379-388.

47. Peng, T., et al., *Tissue-Resident-Memory CD8(+) T Cells Bridge Innate Immune Responses in Neighboring Epithelial Cells to Control Human Genital Herpes.* Front Immunol, 2021. **12**: p. 735643.

48. Wu, X., et al., *CD8(+) Resident Memory T Cells and Viral Infection.* Front Immunol, 2018. **9**: p. 2093.

49. Shin, H. and A. Iwasaki, *Tissue-resident memory T cells.* Immunol Rev, 2013. **255**(1): p. 165-81.

50. Klicznik, M.M., et al., *Human CD4(+)CD103(+) cutaneous resident memory T cells are found in the circulation of healthy individuals.* Sci Immunol, 2019. **4**(37).

51. Casciano, F., et al., *CCR4(+) Skin-Tropic Phenotype as a Feature of Central Memory CD8(+) T Cells in Healthy Subjects and Psoriasis Patients.* Front Immunol, 2020. **11**: p. 529.

52. Cheuk, S., et al., *CD49a Expression Defines Tissue-Resident CD8(+) T Cells Poised for Cytotoxic Function in Human Skin.* Immunity, 2017. **46**(2): p. 287-300.

53. Nizard, M., et al., *Induction of resident memory T cells enhances the efficacy of cancer vaccine.* Nat Commun, 2017. **8**: p. 15221.
