# Supplementary material for: MAdCAM-1 costimulation in the presence of retinoic acid and TGF-β promotes HIV infection and differentiation of CD4+ T cells into CCR5+ TRM-like cells
Source: PLoS Pathog. 2023 Mar 10;19(3):e1011209. doi: 10.1371/journal.ppat.1011209 (PMC10032498; doi:10.1371/journal.ppat.1011209)
Supplement: S4 Table — (DOCX) [file ppat.1011209.s012.docx]

**S4 Table. RT-qPCR oligo sequences**

| **Gene** | **ERGO/GenBank#** | **Type** | **Oligo Name** | **5'-3' sequence** |
| --- | --- | --- | --- | --- |
| CCR7 | NM_001838.4 | Fwd | CCR7-38NM1838F | TGGCTCTCCTTGTCATTTTCC |
|  |  | Rev | CCR7-166NM1838R | AGTTCCGCACGTCCTTCTT |
|  |  | FAM | CCR7-90NM1838FAM | CGATTACATCGGAGACAACACCACAGT |
| SELL | NM_000655.5 | Fwd | SELL-914NM655F | CATCTGGAATCTGGTCAAATCCT |
|  |  | Rev | SELL-1059NM655R | CCAAATGATAAATGCCAACCC |
|  |  | FAM | SELL-997NM655FAM | CCCCTCTTCATTCCAGTGGCAGTCA |
| S1PR1 | NM_001400.5 | Fwd | S1PR1-1555NM1400F | AGTGTGTGCACTTCTGCTTCTTT |
|  |  | Rev | S1PR1-1687NM1400R | ACAACCCCAGCTCTGATAACTC |
|  |  | FAM | S1PR1-1605NM1400FAM | ACCCTCCCTTCCCTTCATACCCCT |
| TAF1D | NM_024116.4 | Fwd | TAF1D-34NM24116F | GTGACATCTGATGCTGTGGAACTT |
|  |  | Rev | TAF1D-193NM24116R | ATGAATCACTTGCGTGAACACTT |
|  |  | CFG | TAF1D-116NM24116CFG | ACTCACCTAAAGGGGAGAAAAGAAACCCCA |
