# Supplementary material for: MAdCAM-1 costimulation in the presence of retinoic acid and TGF-β promotes HIV infection and differentiation of CD4+ T cells into CCR5+ TRM-like cells
Source: PLoS Pathog. 2023 Mar 10;19(3):e1011209. doi: 10.1371/journal.ppat.1011209 (PMC10032498; doi:10.1371/journal.ppat.1011209)
Supplement: S5 Table — (DOCX) [file ppat.1011209.s013.docx]

**S5 Table. Flow cytometry antibodies**

| **Antibody** | **Clone** | **Fluorescence** | **Source** |
| --- | --- | --- | --- |
| anti CD69 | FN50 | APC or APC-H7 | BD |
| anti CD103 | Ber-ACT8 | PE-Cy7 or PE | Biolegend |
| anti CCR5 | J418F1 | BV421 | Biolegend |
| anti CCR9 | L053E8 | PE or PE-Cy7 | Biolegend |
| anti CTLA-4 | BNI3 | BV421 | BD |
| anti PD-1 | EH12.2H7 | APC-Cy7 | Biolegend |
| anti ⍺_4_β_7_ | primatized ACT-1 | Alexa Fluor 488 or 647 | Aftab Ansari |
| anti β_7_ | FIB27 | FITC | Biolegend |
| anti CD45RO | UCHL1 | APC-H7 or FITC | BD |
| anti Foxp3 | 236A/E7 | Alexa Fluor 647 | BD |
| anti CD25 | M-A251 | FITC | BD |
| anti p24 | KC57 | FITC | Beckman Coulter |
| anti p24 | 28B7 | APC | MediMabs |
| anti CD4 | AF700 | RPA-T4 | biolegend |
| anti CXCR3 | APC-Cy7 | G025H7 | biolegend |
| anti CCR6 | PE | L053E8 | biolegend |
